# The dynamic and stress-adaptive signaling hub of 14-3-3: emerging mechanisms of regulation and context-dependent protein–protein interactions

**DOI:** 10.1038/s41388-018-0348-3

**Published:** 2018-06-18

**Authors:** KL Pennington, TY Chan, MP Torres, JL Andersen

**Affiliations:** 1 Department of Chemistry and Biochemistry, Brigham Young University, Provo, UT USA; 2 School of Biological Sciences, Georgia Institute of Technology, Atlanta, GA USA

## Abstract

14-3-3 proteins are a family of structurally similar phospho-binding proteins that regulate essentially every major cellular function. Decades of research on 14-3-3s have revealed a remarkable network of interacting proteins that demonstrate how 14-3-3s integrate and control multiple signaling pathways. In particular, these interactions place 14-3-3 at the center of the signaling hub that governs critical processes in cancer, including apoptosis, cell cycle progression, autophagy, glucose metabolism, and cell motility. Historically, the majority of 14-3-3 interactions have been identified and studied under nutrient-replete cell culture conditions, which has revealed important nutrient driven interactions. However, this underestimates the reach of 14-3-3s. Indeed, the loss of nutrients, growth factors, or changes in other environmental conditions (e.g., genotoxic stress) will not only lead to the loss of homeostatic 14-3-3 interactions, but also trigger new interactions, many of which are likely stress adaptive. This dynamic nature of the 14-3-3 interactome is beginning to come into focus as advancements in mass spectrometry are helping to probe deeper and identify context-dependent 14-3-3 interactions—providing a window into adaptive phosphorylation-driven cellular mechanisms that orchestrate the tumor cell’s response to a variety of environmental conditions including hypoxia and chemotherapy. In this review, we discuss emerging 14-3-3 regulatory mechanisms with a focus on post-translational regulation of 14-3-3 and dynamic protein–protein interactions that illustrate 14-3-3’s role as a stress-adaptive signaling hub in cancer.

## 14-3-3 function

The human 14-3-3 family consists of seven isoforms (β, γ, ε, η, σ, τ, and ζ), each expressed by a different gene. The multiplicity of 14-3-3 isoforms appears to be somewhat common throughout evolutionary history, as budding and fission yeast contain two different 14-3-3 genes, and plants express up to 15 different 14-3-3 isoforms. Other organisms, such as Dictyostelium discoideum and Giardia duodenalis, express only one 14-3-3 gene [1, 2]. In cases where multiple isoforms exist, 14-3-3s are thought to function as hetero- or homodimers, with each monomer consisting of nine α-helices that help form a highly conserved amphipathic groove that serves as the phosphorylation-binding pocket. Within this pocket, positively charged lysine and arginine residues (K49, K120, R56, and R127) coordinate an interaction with the phosphate of the binding partner. The same lysine residues may also serve as shut-off switches for 14-3-3 binding when modified by acetylation (discussed below) [3–5].

14-3-3s interact with phosphorylated serines or threonines within the general consensus sequence RXX**pS/T**XP, although there are many examples that deviate from this motif. For example, the +2 proline occurs in just under half of the known 14-3-3 binding sites [6]. Phosphorylations on the target protein are frequently within regions of high intrinsic disorder and often occur in pairs with each phosphorylation interacting with a positively charged phospho-binding pocket on the 14-3-3 dimer. In some cases, a single phosphorylation is sufficient for 14-3-3 binding, while in others, two are required. Furthermore, when two phosphorylations are required for 14-3-3 binding, they may be added to the target protein by different kinase signaling pathways, which helps explain how 14-3-3s integrate multiple signaling pathways into a single output [7].

The effect of 14-3-3 docking to the phosphorylated protein can vary depending on the protein in question. 14-3-3 binding can mask nuclear localization or export signals, block binding of other proteins (e.g., phosphatases), or contort proteins into active or inhibited conformations (reviewed elsewhere [8, 9]). 14-3-3s may also act as molecular adapters by linking two phosphorylated proteins together [10]. The ability of 14-3-3 to alter the structure of its binding partners has been attributed to its rigid α-helical backbone, which may enforce conformational changes in the binding partner as 14-3-3 binds tightly around the phosphorylated docking sites.

## 14-3-3-mediated chemoresistance

Most of the evidence supporting a role for 14-3-3s in promoting chemoresistance and poor patient outcomes focuses on the 14-3-3ζ gene (*YWHAZ*), which resides within the 8q22.3 chromosomal region which is frequently duplicated in cancer [11, 12]. 14-3-3ζ functions as a central node to promote oncogenic and chemoresistance pathways in cancer including PI3K/AKT, IGF-IR, ERK/MAPK, TGF-β, β-catenin, and hTERT [13–23]. Transgenic overexpression of 14-3-3ζ alone induces tumorigenesis in mice [23]. High levels of 14-3-3ζ expression have been correlated with poor patient outcomes in a variety of cancers, including breast, lung, multiple myeloma, head and neck, and glioblastoma [13, 14, 19, 24–29]. Cell culture studies provide further evidence that overexpression of 14-3-3ζ, as well as other 14-3-3 isoforms, confers chemoresistance. These 14-3-3 isoforms include 14-3-3γ in melanoma cells resistant to cisplatin, etoposide, fotemustine, or vindesine; 14-3-3ε in breast cancer cells resistant to adriamycin or paclitaxel; and 14-3-3ζ in diffuse large B-cell lymphoma (DLBCL) and prostate cancer cells [30–33].

14-3-3ζ depletion has also been shown to sensitize non-small cell lung cancer cells to cisplatin treatment without affecting the proliferation or apoptosis rates of untreated cells in vitro, slow tumor growth in an animal model following cisplatin treatment, and restore sensitivity of DLBCL cells to CHOP-induced cell death [25, 32]. Disruption of 14-3-3-ligand interactions by treatment with the R18 peptide (described below) sensitizes BCR-Abl-transformed murine cells to U0126, GX15-070, and rapamycin-induced apoptosis [34]. Further, 14-3-3ζ overexpression reduced the sensitivity of prostate carcinoma cells to 9NC6 treatment, and 14-3-3ε overexpression protected colorectal carcinoma cells from NSAID-induced apoptosis [33, 35]. Recent evidence also suggests that 14-3-3ζ interacts with proteasome subunits and restrains proteasome activity in multiple myeloma cells, such that depletion of 14-3-3ζ increases proteasome activity and enhances the sensitivity of the cells to the proteasome inhibitor bortezomib [29]. Thus, published studies reinforce the idea that 14-3-3ζ, given its large network of pro-survival interactions, could potentially be targeted to sensitize cancer cells to a wide range of cell death-inducing therapeutics. As discussed below, although we have yet to see a 14-3-3 inhibitor in the clinic, current efforts are underway to develop small molecule disruptors of 14-3-3 interactions.

## 14-3-3s as therapeutic targets

Although pharmaceutical efforts have traditionally focused on developing inhibitors of enzymes (e.g., kinases, G protein-coupled receptors), there has been a recent surge in targeting protein–protein interactions (PPIs). PPI inhibitors include a variety of promising cancer therapeutics such as SMAC and BH3 mimetics, p53-MDM2 antagonists, HSP90 inhibitors, and tubulin polymerization inhibitors [36]. 14-3-3s present an additional high-value target with the potential to disrupt a hub of pro-tumor pathways with a single hit. Historically, the dogma against developing PPI inhibitors has centered around the argument that PPIs often involve large protein surfaces with numerous residues contributing to the free energy of binding [37]. However, 14-3-3s present a well-defined phospho-peptide binding pocket with a relatively small number of positively charged residues (e.g., K49) required for binding, which makes them a seemingly attainable PPI target. Furthermore, for other phosphorylation driven PPIs, such as SH2-phosphotyrosine binding, the phosphate of the binding partner is thought to be the primary contributor of the free energy of binding [38]. Thus, small molecule disruption of even a single positively charged residue, like K49, within the 14-3-3 phospho-binding pocket, would likely abrogate 14-3-3 interactions.

Several groups have focused on developing peptide and peptidomimetic inhibitors of 14-3-3s, which have been reviewed in detail elsewhere [39–42]. Among these are some notable leads. A phage display approach generated a short peptide, termed R18, which has a stretch of negatively charged Glu and Asp residues that mimic phosphorylation by binding within the positively charged phospho-binding pocket of 14-3-3 [43–45]. Fu and colleagues generated an expressible dimer form of R18, called difopein, which disrupts 14-3-3 interactions and sensitizes cultured cells to cisplatin-induced apoptosis and inhibits growth of xenografted glioma cells in mice [46, 47]. These studies helped establish a proof-of-concept for targeting 14-3-3s in vivo [46].

Several small-molecule 14-3-3 inhibitors have been developed, but, to our knowledge, few have moved past in vitro proof-of-concept. Botta and colleagues developed a non-peptidic 14-3-3 inhibitor that effectively induces apoptosis of imatinib-resistant BCR-Abl-expressing leukemia cells [48, 49]. The same group further optimized their compounds and settled on two 14-3-3 inhibitors that sensitize multi-drug resistant non-small-cell lung carcinoma and colon carcinoma cell lines to doxorubicin-induced apoptosis [50]. Effective doses of these compounds were in the low μM range in cultured cells. Other efforts have identified in vitro inhibitors of 14-3-3 [51–53]. For example, Fu and colleagues identified a peptidomimetic small molecule, termed FOBISIN101, which interacts with K120 and K49 within the 14-3-3 phosho-binding pocket. Interestingly, X-ray irradiation induces a covalent link between FOBISIN101 and K120, which suggests that it could serve as a radiation-activated drug [54].

**Fig. 1:**
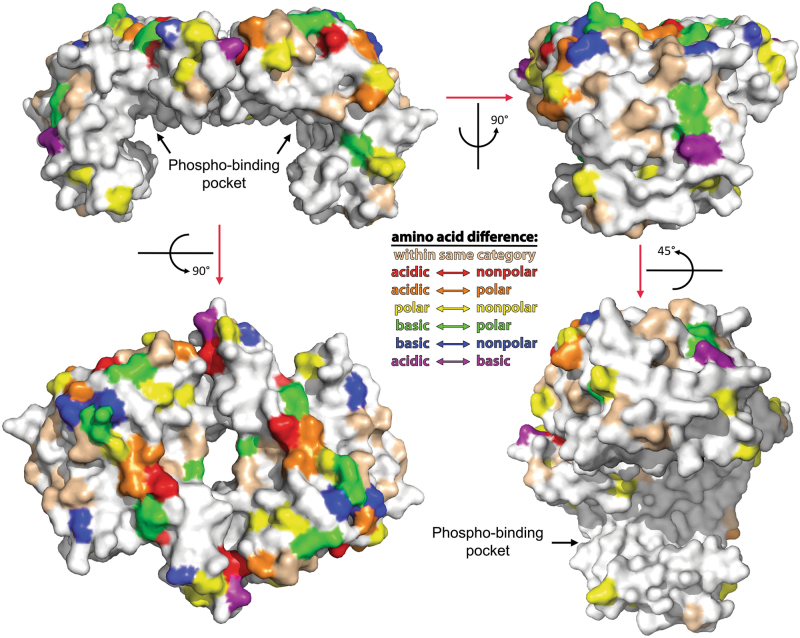
Amino acid divergence between σ and ζ isoforms superimposed over the 14-3-3ζ structure. The 14-3-3ζ crystal structure (PDB ID: 4FJ3, accessed from RCSB PDB: www.rcsb.org) was color coded to illustrate amino acid differences between the σ and ζ isoforms using PyMOL (Schrödinger, New York, NY, USA). White residues correspond to amino acids that were identical between the two isoforms. Each amino acid was categorized according to its side chain chemistry, and the categorical changes for each variable residue were color coded according to the table in the center. Each image represents differing views of the 14-3-3ζ dimer, as indicated by the rotational angles between each image. Note that the amino acids forming the phospho-binding pocket are identical between the two isoforms

Although these efforts have generated promising lead compounds, important questions remain: do any of the compounds show specificity for any specific 14-3-3 isoform(s) in cells? Some compounds reportedly show specificity for the ζ isoform, but mechanisms to explain their specificity are still lacking [55, 56]. In light of the highly conserved phospho-binding pockets of 14-3-3 isoforms (Fig. 1), it is difficult to imagine any isoform selectivity by targeting this region. However, one potential strategy to generate specificity is to design a molecule that targets the phospho-binding pocket and also interacts with the divergent C-terminal tail of a specific 14-3-3 isoform [56]. Another question relates to whether targeting a specific isoform is necessary or even advantageous in a cancer setting. As discussed in more detail below, many cancers seem to rely on high levels of 14-3-3ζ expression, but given the functional overlap between different isoforms, targeting the ζ isoform specifically may simply select for compensation by other 14-3-3 isoforms. Thus, a compound that targets multiple 14-3-3 isoforms may be advantageous in an acute treatment scenario.

## Differential regulation of 14-3-3 isoforms in cancer

A comparison of crystal structures from different human 14-3-3 isoforms shows striking structural similarities [8]. As one might expect given the similarity, there is functional overlap between 14-3-3 isoforms, and the ability of one isoform to compensate for the loss of another has been demonstrated in some cases [57, 58]. Nevertheless, there are isoform-specific functions. The clearest functional outlier within the 14-3-3 family is 14-3-3σ, which plays a tumor suppressive role in promoting DNA damage checkpoints and p53 activation [59]. Accordingly, its expression is epigenetically downregulated in breast and other cancers [60–62]. On the other end of the spectrum is 14-3-3ζ, which has been extensively characterized as a driver of pro-survival signaling. However, the distinction between these isoforms is not always clear-cut, as 14-3-3σ is overexpressed in some cancers, including colon carcinoma and pancreatic ductal carcinoma, where it has been shown to promote cell survival and invasiveness [63–65]. Nevertheless, on an aggregate, the published data suggest clear distinctions, relevant to cancer, between the σ and ζ isoforms.

Although the amino acid sequences and overall structure of the ζ and σ isoforms are quite conserved, there are some regions of divergence. Superimposing the amino acid differences of the σ isoform over the 14-3-3ζ crystal structure shows high conservation in the phospho-binding pockets, while the majority of amino acid differences occur on the outside surfaces of the protein (Fig. 1) [66, 67]. This is supported by structural studies that suggest that the core mechanism of phosphate docking within the 14-3-3 binding pocket is conserved across isoforms [8, 68, 69].

How then are the 14-3-3ζ and σ isoforms differentially regulated? One explanation, proposed by Yaffe and colleagues, is based on the divergence within the C-terminal 15 amino acids of ζ and σ, which is the most divergent stretch of amino acids between the two proteins. This region is flexible and therefore not captured in the crystal structure. Molecular modeling studies suggest that this C-terminal region, which contains negatively charged Glu and Asp residues, may interact with the phospho-binding pocket of 14-3-3s to block interactions, and thus may be a point of regulation to differentiate isoform function [8, 69]. In addition, our analysis of 14-3-3 post-translational modifications (PTMs) by SAPH-ire FPx, presented in detail below (see PTM regulation of 14-3-3), uncovered a striking PTM cluster within the C-terminus that encompasses the final 15 amino acids of 14-3-3ζ. This cluster includes several phospho-sites of unknown function including T205, S207, S210, T229, and S230 (human 14-3-3ζ numbering). It seems likely that these phosphorylations could control the regulatory activity of the 14-3-3 C-terminus.

Truncation of this C-terminal region increases the 14-3-3ζ affinity to phosphorylated Raf and Bad [70]. In addition, mutation of residues unique to the C-terminus of 14-3-3σ render it capable of binding Cdc25C, which normally interacts exclusively with other 14-3-3 isoforms [69]. There is also abundant evidence for tissue-specific expression of 14-3-3 isoforms in plants and animals and isoform-specific localization within the same tissues [71–74]. Some evidence suggests that the localization of isoforms depends on their binding partners, as disruption of specific 14-3-3 interactions shifts 14-3-3 localization and binding-defective mutants of 14-3-3 accumulate within the nucleus [71, 75]. In addition, Yu and colleagues recently demonstrated that 14-3-3ζ transcriptionally represses 14-3-3σ in breast cancer by binding and sequestering YAP1, which shifts the isoform balance from 14-3-3σ-mediated growth suppression via p53 to 14-3-3ζ-mediated cell growth and TGF-β-induced bone metastasis [19]. Given that the YWHAZ gene is situated within the 8q22 chromosomal region, which is frequently amplified in cancer, it is possible that the resulting increase in YWHAZ expression alone may position 14-3-3ζ as a dominant 14-3-3 isoform in cancer.

Finally, another proposed explanation for functional differences between the isoforms is their tendency to homo- or heterodimerize. 14-3-3σ displays a strong tendency to homodimerize, likely aided by amino acids and salt bridges unique to the σ isoform at its dimer interface [8, 69]. All other human isoforms seem to readily heterodimerize. Indeed, our laboratory’s proteomics efforts to identify 14-3-3ζ binding partners consistently find abundant peptides from all 14-3-3 isoforms, except σ, in 14-3-3ζ immunoprecipitates. This tendency of non-σ isoforms to heterodimerize can make the determination of isoform-specific functions difficult. It is probably for this reason that many 14-3-3 studies do not explicitly claim a particular isoform. Accordingly, in the paragraphs below, we indicate the isoform when clearly warranted, but otherwise leave it open to question.

## Mechanisms that regulate 14-3-3 dynamics: 14-3-3 ‘sinks’ and post-translational modifications

### 14-3-3 binding dynamics

14-3-3ζ levels can be relatively stable within a given tissue or cell type, and yet changes in the cellular conditions can drive rapid shifts in the 14-3-3 interactome, which may include expansion or shrinkage in the number of proteins bound. A long-standing question in the 14-3-3 field relates to the regulation of these interactions: what drives 14-3-3 binding dynamics and allows for rapid shifts in its interactome? In a simple model, a free pool of 14-3-3 is poised to bind targets once they are phosphorylated. In this scenario, phosphorylation of the binding partner helps to drive the 14-3-3 binding dynamics. While this model does not explain the entire complexity of 14-3-3 regulation, the concept of a free 14-3-3 pool is backed up by some biochemical data. In Xenopus egg extract experiments, dipping an in vitro-phosphorylated protein into the extract can recover high levels of bound 14-3-3, suggesting free, available 14-3-3 [5, 76]. Furthermore, changes in kinase signaling pathways (e.g., limiting nutrients, insulin) can dramatically alter some 14-3-3 interactions. In our experience, this occurs in the absence of any clear LC-MS/MS-detectable PTM changes on 14-3-3 itself. Moreover, the kinetics of 14-3-3 binding often correlate in a fairly linear way with phosphorylation of the binding partner, and loss of 14-3-3 binding tracks well with the loss of upstream kinase activity and binding partner phosphorylation [18, 77, 78]. However, these basic observations do not exclude the existence of other contributors to 14-3-3 binding dynamics, as discussed below.

An intriguing, albeit still preliminary, model of 14-3-3 regulation is the existence of 14-3-3 ‘sinks’ that sequester 14-3-3 away from binding partners. A trigger of cell cycle progression from DNA checkpoints into M phase is the release of 14-3-3 from Cdc25, which is dephosphorylated upon transition into M phase. However, 14-3-3 release is not solely dependent on the Cdc25 phosphorylation state, but also requires the sequestering of 14-3-3 into phosphorylated keratin filaments [79]. This suggests that removal of 14-3-3 from a binding partner may be reinforced by alternative docking sites for 14-3-3 on abundant proteins— essentially creating a sink. A similar mechanism was proposed to explain the release of 14-3-3 from TSC2 in hypoxia, wherein the creation of an alternative docking site on REDD1 sequesters 14-3-3 away from TSC2 and promotes the inhibition of mTORC1 [80]. The abundant proteins vimentin and heat shock protein 6 have also been proposed to act as 14-3-3 sinks [81, 82]. The sink could help explain the dynamic shrinking and expansion of the 14-3-3 interactome without significant changes in 14-3-3 synthesis or degradation. Further studies into the *K*_d_ of 14-3-3 binding to each of these proteins could help to better delineate this model.

### PTM regulation of 14-3-3

**Fig. 2:**
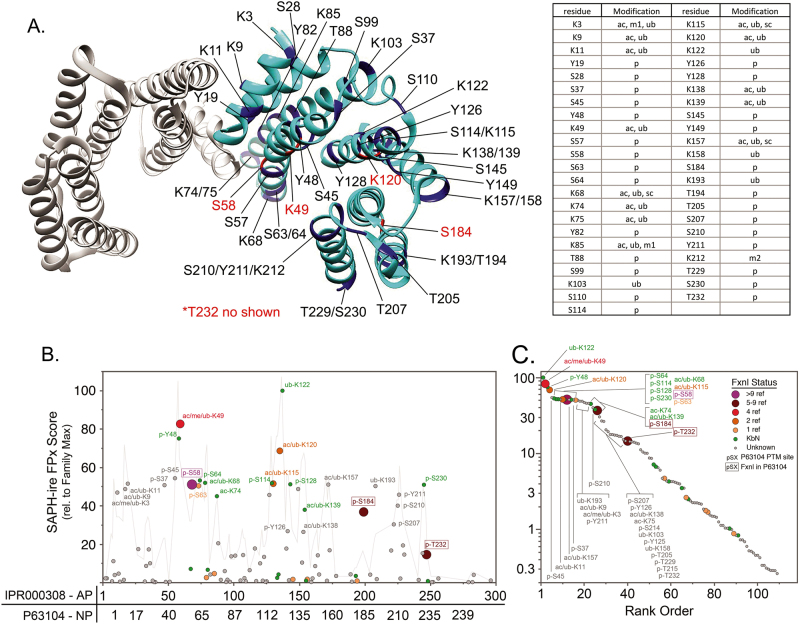
SAPH-ire analysis of 14-3-3 protein family IPR000308 and relationship to 14-3-3ζ (P63104). **a** Crystal structure of 14-3-3ζ dimer (PDB ID: 4FJ3, accessed from RCSB PDB: www.rcsb.org) with highlighted PTM sites shown on one of the monomers. Table on the right indicates the PTM identities at each site, as reported from aggregated proteomics data on dbPTM3 and PhosphoSitePlus (phosphosite.org). **b** A total of 654 distinct PTMs observed across 37 14-3-3 proteins from 10 different eukaryotic species were aligned and analyzed by SAPH-ire, resulting in 109 modified alignment positions (MAPs). Each MAP is represented as a single circle relative to the alignment position of the family. **c** Each MAP is represented as relative to the SAPH-ire probability-derived rank order. For panels **b** and **c**, circle size and color indicate MAP status and graphically reflects the number of sources from which known-functional status is derived for the MAP (colored circles), known-by-neighbor (KbN) status (green circles), or unknown functional status (gray circles). MAPs are labeled corresponding to the native position of 14-3-3ζ (P63104) that falls within the MAP. Functional PTM sites within 14-3-3ζ (P63104) are indicated with boxes. The relative concentration of the observed PTMs (taken as the “cluster count” of modified residues within ±2 alignment positions surrounding the MAP) is shown to highlight dense PTM clusters along the primary structure of the protein family (**b**, gray line). (See also supplementary Table S1.)

Another layer of regulation exists at the level of 14-3-3 itself. At least a subset of 14-3-3 interactions are also modulated by PTMs on 14-3-3 [83]. Over the last decade, MS approaches have uncovered a wide array of PTMs across the proteome. Aggregated proteomics data indicate at least 66 PTMs on 14-3-3ζ [84–86]. Some of these are shown scattered across the ζ crystal structure in Fig. 2a and include phosphorylation, acetylation, methylation, s-nitrosylation, and ubiquitination. Like for most proteins, our understanding of their impact on 14-3-3s lags far behind the pace of PTM discovery; only six of the 66 have published data that support any impact on 14-3-3 function. Furthermore, our understanding of these potentially functional PTMs on 14-3-3 is still in its infancy, as most have not been subjected to rigorous analysis [87].

Figs. 2b, c show an analysis of all MS-identified 14-3-3ζ PTMs by SAPH-ire FPx, a neural network-based PTM “hot spot” finder that prioritizes PTMs for the likelihood of biological function based on several previously described parameters, including the number of times the PTM has been observed in MS data and the evolutionary conservation of the modified amino acid within protein domain families (see also Table S1) [88–90]. Based on these parameters, each PTM is assigned a probability score, with higher scores suggesting an increased likelihood of biological function. Strikingly, three of the 14-3-3ζ phosphorylations (S58, S184, T232) that have an established biological function rank within the top ~ 30% of all phosphosites (discussed further below). Several additional high-ranking sites with known functional impact include acetylation sites at K49, K115, and K120 (discussed below), which occur on multiple isoforms (β, γ, and ζ) [3, 4, 91]. In addition, phosphorylation at an S63-aligned residue in rat 14-3-3β regulates nuclear localization of HDAC4 and subsequent MEF2-dependent gene silencing in smooth muscle (PMID 12619878). The co-alignment of this phosphorylation with 14-3-3ζ suggests that its functional impacts may be translatable across isoforms [92].

Several other high-ranking PTM sites on 14-3-3 proteins have never been reported as functional, presumably because they have not been tested. In this regard, tools like SAPH-ire may be useful for prioritizing functional studies. In many cases, high-ranking modification sites can be loosely associated with function simply through association with nearby neighboring PTMs (within 2 residues) that are known to be functional (Fig. 2b, c. green circles). In many other cases, the high rank of a PTM site cannot be explained and provides potential avenues for further discovery (Fig. 2b, c. gray circles). Several of these *unknown function* PTMs are observed in 14-3-3ζ, including acetylation/ubiquitination sites within a PTM cluster in the N-terminal region (K3, K9, K11), an alpha helix that helps form the phospho-binding pocket (K138, K157), and near regions known to control binding partner interactions (K193). Several high-ranking phosphosite-containing MAPs are observed in 14-3-3ζ specifically, including sites along the central α-helix of the phospho-binding groove (S37, S45), near acetylation sites known to modulate binding partner interactions (Y126), and in an uncharacterized phosphosite cluster near the C-terminus that has no known function to date (S207, S210, and Y211). In particular, S37 and S45, which rank within the top 4 of all 14-3-3 PTMs of unknown function, are of great interest due to their proximity to the phospho-binding pocket, which also includes positively charged residues (e.g., K49) required for 14-3-3 binding.

Of the 14-3-3 PTMs with known function, S58 of 14-3-3ζ was initially identified as an AKT substrate, and its position at the dimer interface suggests a role in regulating dimer formation [93]. Cross-linking and native gel analysis of 14-3-3 from cultured cells demonstrated that S58 phosphorylation is only detected on the 14-3-3 monomer, which appears to be at very low stoichiometry, relative to dimerized 14-3-3. One lingering question is how this surface of 14-3-3, normally masked by a very stable dimer interaction, is accessible to a kinase. S58 might be phosphorylated shortly after translation and prior to dimerization or certain stimuli may promote a shift of 14-3-3 from dimer to monomer. Regarding the latter possibility, there is evidence that the cationic lipid sphingosine may bind directly to 14-3-3 to promote its monomerization and accessibility of S58 to sphingosine-induced PKA or PKC activity [93–95]. This sphingosine-induced change in 14-3-3 was shown to sensitize cells to apoptosis via the release of pro-apoptotic Bax from 14-3-3, resulting in Bax activation and outer mitochondrial membrane permeabilization (MOMP) [96]. Thus, inactivation of 14-3-3’s anti-apoptotic interactions via monomerization may constitute a key hurdle that sphingosine overcomes to sensitize cells to death. Interestingly, a shift to monomeric 14-3-3 may also increase 14-3-3’s poorly understood chaperone-like activity, which involves an unconventional mode of 14-3-3 binding that does not require phosphorylation of the binding partner [97, 98].

T/S232 of 14-3-3 (serine or threonine depending on the isoform) was identified as a casein kinase 1 alpha substrate and is positioned within the intrinsically disordered C-terminal tail [99, 100]. T232 phosphorylation was shown to inhibit 14-3-3ζ binding to c-RAF, and a phospho-mimicking glutamate mutation at the same position abrogated 14-3-3*θ*-mediated cell survival in a model of Parkinson’s disease, suggesting that this phosphorylation may broadly impact 14-3-3 interactions [101]. Based on its position within the C-terminal tail, it is tempting to speculate that T232 phosphorylation may promote wedging of the C-terminal tail into the 14-3-3 phospho-binding pocket and thus may be an additional PTM-based mechanism to differentially control 14-3-3 isoforms [102]. 14-3-3ζ is also phosphorylated at S184 by c-JNK, and this phosphorylation stimulates release of pro-apoptotic proteins Bad and FOXO3A. S184 lies outside the phospho-binding pocket, but is in position to interact with binding partners adjacent to the minimal 14-3-3 binding motif (Fig. 1) [103–105].

A more recent discovery is the presence of acyl modifications on lysine residues around the 14-3-3 phospho-binding pocket. Structural studies of 14-3-3 proteins first highlighted the contribution of these lysines in coordinating interactions with the phosphate of the binding partner [44, 106]. This effect is dependent on the positive charge of the lysines, as charge-changing mutations at K49 render 14-3-3s defective in binding. Later, in 2009, a large-scale proteomics effort uncovered acetylations at several lysines within and around the 14-3-3 binding pocket, including K49 and K120 [4]. These acetylations, which neutralize the positive charge of lysine, are prime candidates to regulate 14-3-3 interactions. In support of this idea, lysine-to-glutamine mutations at K49 or K120, which mimic the change in charge of acetyl-lysine, inhibit 14-3-3 interactions [4].

Clues on how these acetylations could control 14-3-3 come from an understanding of their regulation. Like phosphorylation, lysine acetylation is controlled by acetyl-adding enzymes (lysine acetyltransferases or KATs) and acetyl-removing enzymes (lysine deacetylases or KDACs). The first link between a KDAC and 14-3-3 came when 14-3-3ζ was discovered as a substrate of the NAD +-dependent KDAC SIRT1 in Xenopus eggs [5]. In this system, high NAD + levels promote SIRT1 activity, which maintains 14-3-3ζ in a deacetylated active state. In turn, this helps to maintain 14-3-3ζ binding to caspase-2 and potentially other partners. Conversely, a drop in NAD + correlates with an increase in 14-3-3ζ K49 acetylation and release of 14-3-3ζ from caspase-2, which activates downstream apoptotic signaling in the egg [5, 76]. In mammalian cells, the deacetylation of 14-3-3ζ is dependent on the cytosolic KDAC HDAC6, with very little contribution from SIRT1 [3]. Inhibition of HDAC6 stimulates acetylation at K49 and K120, which triggers release of 14-3-3ζ from several binding partners, including Bad, AS160, and kinesin. Arginine mutations at these lysines, which maintain the positive charge but cannot be acetylated, make 14-3-3ζ resistant to this mode of regulation, allowing 14-3-3ζ to stay bound to its partners despite HDAC6 inhibition [3].

**Fig. 3:**
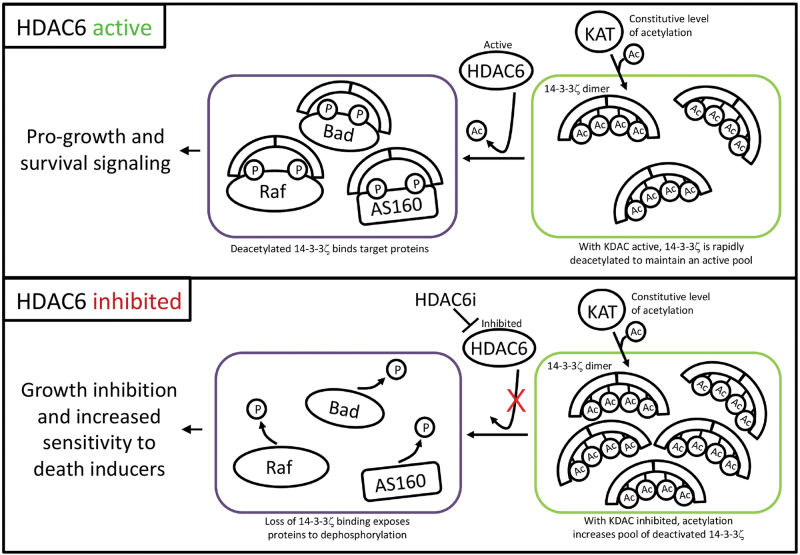
Model of 14-3-3ζ regulation by acetylations within the 14-3-3ζ binding pocket. HDAC6-mediated deacetylation of K49 and K120 maintains the 14-3-3ζ phospho-binding pocket free for interactions with phospho-proteins, which drive pro-growth and survival signaling. Inhibition of HDAC6 allows an unidentified KAT to acetylate and inhibit the unbound pool of 14-3-3ζ, which, in turn, leads to a loss of pro-growth/survival 14-3-3ζ interactions

One unanswered question is how these lysines are acetylated or deacetylated given that they are buried between 14-3-3 and its binding partner. We posit that the KDAC or KAT could only gain access to K49 and K120 when 14-3-3 is in an unbound state. If so, this argues for an actively regulated pool of free 14-3-3. If we assume 14-3-3 binding is dynamic and subject to an on-off rate, inhibition of the KDAC or activation of the KAT would build up acetylation on free 14-3-3 and drive the equilibrium of total 14-3-3 toward its unbound state (Fig. 3). This may effectively act as a sink to decommission a pool of 14-3-3ζ. This idea is supported by published data showing that K49 acetylation is required for the loss of several functionally diverse 14-3-3 binding partners in cells treated with an HDAC6 inhibitor [3].

Additional questions relate to how acetylation may regulate 14-3-3s spatially. For example, does inhibition of HDAC6 lead to loss of 14-3-3ζ interactions on a global scale or is just a small subset of interactions affected? Could different subcellular pools of 14-3-3 have their own dedicated KDAC? Although inhibition of SIRT1 does not have a dramatic effect in mammalian cells on 14-3-3ζ acetylation globally, it may affect a specific pool, such as nuclear 14-3-3ζ. Given the diversity of 14-3-3ζ interactions and their varied subcellular locales, it seems likely that such nuanced modes of regulation would exist, which could help explain how 14-3-3’s vast interactome is dynamically regulated both spatially and temporally.

## Dynamic 14-3-3 mechanisms promote the metabolic plasticity of cancer cells and drive critical cancer processes

### Insulin and glucose signaling

**Fig. 4:**
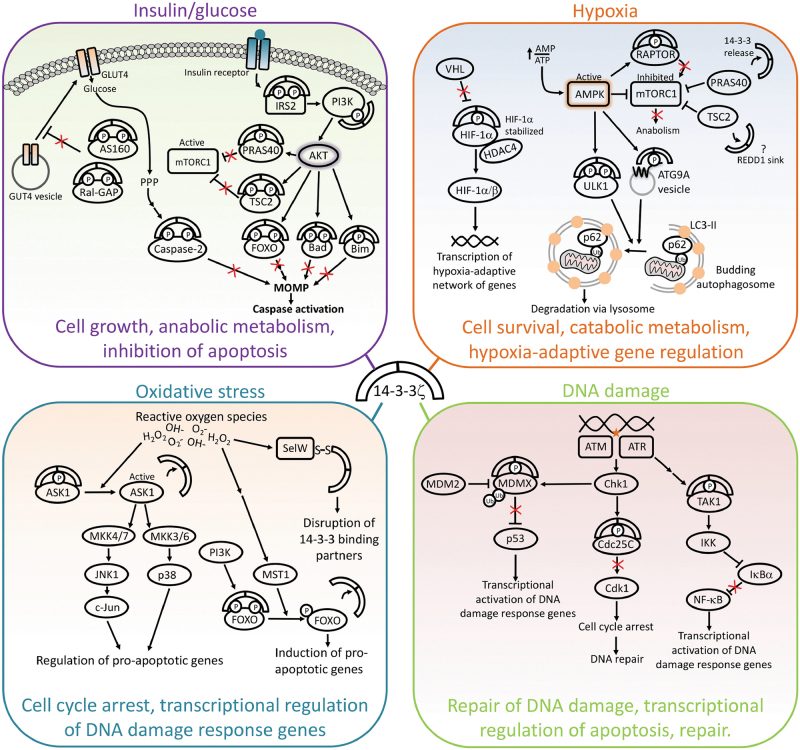
Dynamic 14-3-3 mechanisms drive the adaptive cellular response to stress and nutrient availability. Shown here are 14-3-3 interactions that drive mechanisms of cellular adaptation, as described in the text

One striking feature of many 14-3-3 interactions is their relationship to glucose and insulin signaling. 14-3-3 proteins are essential for every phase of insulin signaling, from the immediate transduction of the insulin signal, to glucose uptake, to downstream transcriptional responses (Fig. 4 and Table S2). Upon insulin binding to its receptor, 14-3-3 interacts with phosphorylated insulin receptor substrate-2 (IRS2), a direct intracellular effector of the insulin receptor. This interaction stabilizes IRS2 to propagate the signal from the receptor to downstream targets, one of which is phosphoinositide 3-kinase (PI3K) [107]. PI3K is put into position at the plasma membrane to receive the signal from IRS2 by an interaction between the PI3K subunit p85 and 14-3-3, which allows IRS2 to fully activate the membrane-associated PI3K complex [24]. Downstream of PI3K, active AKT phosphorylates the forkhead transcription factor FOXO, which triggers a 14-3-3-FOXO interaction that keeps FOXO out of the nucleus. 14-3-3-mediated inhibition of FOXO prevents upregulation of numerous FOXO target genes, including the pro-apoptotic effectors TRAIL, Puma, and Bim. In this manner, 14-3-3s enforce inhibition of the apoptotic program under nutrient-replete conditions.

Downstream of the immediate signaling events, 14-3-3s also play multiple roles in promoting GLUT4 trafficking to promote glucose uptake at the cell surface. 14-3-3 inhibits the Rab-GTPase-activating protein (Rab-GAP) AS160/TBC1D4, which allows for GLUT4 trafficking to the plasma membrane (reviewed in [7]). Remarkably, a single knock-in mutation within AS160 that abrogates the 14-3-3 binding site (T649A, an AKT phospho-site) renders mice glucose intolerant and unable to properly traffic GLUT4 [108]. In muscle tissue, 14-3-3 also interacts with the Rab-GAP TBC1D1 and GARNL1, a subunit of Ral-GAP, to promote GLUT4 trafficking [7, 109, 110]. Insulin also stimulates phosphorylation of myosin 1c at S701 and 14-3-3 binding, which is required for GLUT4 transport to the cell surface [111, 112].

Further downstream of insulin/glucose signaling, 14-3-3ζ interacts with and inhibits apoptotic effectors, such as Bad, caspase-2, and Bim, to promote cell survival [76, 113–115]. One of the best-studied 14-3-3ζ interactions is the pro-apoptotic BH3 only protein Bad. In response to AKT signaling, 14-3-3ζ sequesters Bad away from pro-survival Bcl-2 proteins (Bcl-xL and Bcl-2), which, in turn, allows Bcl-xL and Bcl-2 to suppress Bax/Bak-mediated MOMP [113, 114, 116, 117]. Glucose metabolism through the pentose phosphate pathway triggers a Ca^2+^/Calmodulin-dependent kinase-II (CAMKII)-dependent interaction between 14-3-3ζ and caspase-2. The resulting inhibition of caspase-2 prevents apoptotic signals from activating pro-apoptotic Bcl-2 family members upstream of MOMP [76, 118].

Several other 14-3-3 binding partners have been identified downstream of insulin and nutrient pathways. In particular, a focus of 14-3-3 seems to be in regulating mTOR-mediated growth signaling. 14-3-3 sequesters away the negative regulators of mTOR, PRAS40 and TSC2. These interactions are mediated by insulin/glucose-stimulated AKT, which phosphorylates PRAS40 at T246 and TSC2 at multiple potential 14-3-3 binding sites (S939, S981, and T1462). Phosphorylation and 14-3-3 binding to TSC2 prevents its inhibition of the mTORC1 activator Rheb at the plasma membrane. These interactions result in the derepression of mTOR and activation of mTORC1-mediated anabolic metabolism and cell growth [119, 120]. On the other hand, perhaps less appreciated is the role of 14-3-3 proteins in supporting cellular adaptation to conditions in which mTOR activity is inhibited. Several mechanisms, including very recent discoveries, illustrate how nutrient deprivation, which occurs in tumor hypoxia, can rearrange the 14-3-3 interactome toward interactions that directly inhibit mTORC1, activate autophagy, and thereby promote an anabolic-to-catabolic shift in metabolism. These mechanisms may play an equally important role to the insulin/glucose-stimulated 14-3-3 interactions in promoting cancer growth, given that tumor cells must adjust to frequent changes in nutrient availability.

### Hypoxia

One of the obstacles a growing tumor faces is the lack of adequate blood supply, which deprives a cell of oxygen, glucose, growth factors, and amino acids. Here we refer to this condition as hypoxia. As described above, 14-3-3 promotes key signaling nodes downstream of glucose and insulin, and in a quick turn of events, 14-3-3 plays a central role in shutting the same pathways off when glucose and insulin are depleted during hypoxia. This not only occurs by 14-3-3 releasing its binding partners, but also through acquiring new hypoxia-triggered interactions (Fig. 4 and Table S2).

Under hypoxic conditions, the sequential shutdown of glucose- and insulin-stimulated kinase pathways and the activation of kinases like AMP-activated protein kinase (AMPK), a central regulator of the adaptive shift from anabolic to catabolic metabolism, results in a rearrangement of the 14-3-3 interactome [18]. Active AMPK phosphorylates several 14-3-3 targets, including the negative regulator of mTORC1, RAPTOR [121]. This interaction is mediated by two AMPK phosphorylation sites within RAPTOR and results in RAPTOR dissociation from mTOR. Induction of hypoxia or other cellular stresses also promotes REDD1 disruption of the 14-3-3-TSC2 interaction to allow TSC2-dependent inhibition of mTORC1 activity and subsequent growth inhibition [80]. The mechanism for REDD1-mediated disruption of the 14-3-3-TSC2 interaction was proposed to occur via direct competition between REDD1 and TSC2 for 14-3-3 binding at the plasma membrane, a model which has subsequently had mixed support, particularly with regards to the direct 14-3-3-REDD1 interaction [80, 122–124]. Collectively, these 14-3-3-mediated mechanisms ensure the proper inhibition of mTORC1, which initiates many of the major adaptive responses to hypoxia, including a rewiring of cellular metabolic pathways and the activation of autophagy to recycle cellular components and consolidate core survival functions. Furthermore, Teo et al. observed increased 14-3-3γ expression with tumor stage for breast, colorectal, gastric, and head and neck cancers, which corresponded to prolonged hypoxia-dependent increases in 14-3-3γ expression which they showed mediate inhibition of TSC2 and also activation of Snai1 to promote an epithelial-mesenchymal transition (EMT), thus highlighting a 14-3-3-mediated adaptation acquired by tumor cells to promote survival [125].

14-3-3ζ regulates other AMPK substrates, downstream of mTORC1, that promote hypoxia-induced autophagy. AMPK-mediated phosphorylation of Unc51-like kinase (ULK1) at S555 triggers 14-3-3 binding, and cells expressing a phospho-defective mutant of ULK1 fail to degrade mitochondria via the autophagy pathway [126, 127]. Downstream of mTOR and ULK1, active AMPK also phosphorylates the core autophagy regulator ATG9A at S761, which triggers 14-3-3ζ binding [18]. The interaction between 14-3-3 and ATG9A promotes ATG9A-mediated growth of autophagosomes, which, in turn, sequester and recycle macromolecules and organelles to sustain cellular metabolism under starved conditions [18, 128–131].

14-3-3ζ interacts with other core regulators of the hypoxic response, including the hypoxia-inducible factor 1 (HIF1) alpha subunit. HIF1 modulates expression of genes involved in angiogenesis, cell proliferation/ survival, and metabolism to facilitate the cellular response to hypoxia as well as tumor adaptations and metastasis [132]. Under normoxic conditions, the HIF1α subunit is ubiquitinylated and rapidly degraded. However, upon induction of hypoxia, the HIF1α subunit is stabilized, binds to the HIF1β subunit, and translocates to the nucleus to modulate transcription of its target genes. Hypoxic conditions increase both 14-3-3ζ expression and binding to HIF1α, which leads to the stabilization of HIF1α protein [133]. 14-3-3ζ overexpression in the absence of hypoxic conditions also increases HIF1α expression in a PI3K/AKT and NFκB dependent manner [133, 134]. Tang et al. propose that 14-3-3ζ facilitates this regulated HIF1α stabilization by binding to both HIF1α and histone deacetylase 4 (HDAC4), acting as a scaffold to bring them together and thereby promoting HDAC4-dependent deacetylation of HIF1α, which is known to contribute to HIF1α stability [133, 135]. This pro-survival role of 14-3-3ζ in stabilizing HIF1α is necessary for hypoxia-induced expression of genes, including those indicative of EMT, which leads to tumor metastasis [133].

### DNA damage

14-3-3s regulate the cellular response to DNA damage primarily by inhibiting untimely progression of the cell cycle to allow time either for DNA repair mechanisms to function before entering mitosis or for cell death pathways to proceed if needed (Fig. 4 and Table S2). DNA damage induced G_2_ arrest requires the maintenance of Cdk1 pT14/pY15, which is accomplished by inhibitory phosphorylation of the phosphatases Cdc25B (at S151, S230, and/or S323) and Cdc25C (at S216) by Chk1, Chk2, or MK2 in response to DNA damage and by C-TAK1 during interphase [136–140]. 14-3-3ζ binds to phosphorylated Cdc25B and Cdc25C to both mask the Cdk1 binding sites and to sequester them in the cytosol, thereby preventing derepression of Cdk1 [141–147]. The inhibitory 14-3-3-Cdc25C association is further maintained by the phosphorylation state of Cdc25C T130. During interphase and G_2_ arrest, PP2A keeps Cdc25C T130 in a dephosphorylated state, which is favorable for 14-3-3 binding [79]. Prior to entry into mitosis, Cdk2 phosphorylates T130, disrupting the 14-3-3-Cdc25C complex and thereby increasing PP1-mediated removal of the S216 phosphate [148, 149]. This allows nuclear localization of Cdc25C, dephosphorylation of Cdk1, and cell cycle progression. During mitosis, Cdk1 activity is sustained by a positive feedback loop in which Cdk1 phosphorylates Cdc25C at S214, which then also helps reduce phosphorylation at S216 and continued progression through the cell cycle [150]. Rather than inhibiting phosphatase enzymatic activity, binding of 14-3-3 to Cdc25A promotes Cdc25A degradation [151, 152]. 14-3-3γ was shown to act as a scaffold to bring together Chk1 and Cdc25A, which facilitates Chk1-mediated phosphorylation of Cdc25A S76 and subsequent polyubiquitinylation and proteasome-dependent degradation [152]. 14-3-3ζ/β also binds to Chk1 in response to DNA damage, promoting nuclear retention of Chk1 and maintenance of the G_2_ checkpoint [153].

NF-κB signaling is also modulated by 14-3-3s upon DNA damage. ATM activates TAK1, which promotes NF-κB translocation to the nucleus to upregulate pro-survival transcriptional networks [154]. 14-3-3ε dynamically binds to both phospho-activated TAK1 and the phosphatase PPM1B to regulate the activation and deactivation of TAK1 [155]. Zuo et al. proposed that 14-3-3ε firstly promotes TAK1 activity by disrupting the TAK1-PPM1B interaction, which prevents cellular apoptosis under initial mild stress; upon sustained stress, 14-3-3ε promotes the inhibitory TAK1 dephosphorylation to allow apoptosis [155]. With high dose DNA damaging agents, 14-3-3ε binding to TAK1 and its interacting protein TAB1 inhibits the anti-apoptotic activity of TAK1, in line with the hypothesis that increasing stress severity switches the 14-3-3-TAK1 interaction from activating to inhibiting [156].

14-3-3s have been demonstrated to promote p53 activity by direct phospho-dependent binding of p53 and by binding its negative regulator, MDMX. 14-3-3 binding to p53 is dependent on phosphorylated S366, S378, and T387 and promotes p53 tetramerization and increased DNA binding affinity [157, 158]. MDMX is involved in maintaining the balance between resistance to stress and inhibition of tumorigenesis, through regulating p53 and through p53 independent mechanisms [159, 160]. Under basal growth conditions, MDMX binds to and inhibits p53 transcriptional activity [161]. DNA damage, hypoxia, and metabolic stress each induce 14-3-3 binding to MDMX, which disrupts MDMX-p53 and promotes MDMX degradation, resulting in p53 stabilization and activation [159, 162–167]. Due to its potent inhibition of p53 and frequent overexpression in tumor samples, MDMX function has become a target of interest for chemotherapeutic agents [168].

Genotoxic stress also induces a variety of interactions among 14-3-3s and other DNA damage-responsive proteins. The activity of Exo1 in resecting DNA ends is antagonized by binding of 14-3-3s to avoid excessive resection in response to damage [169–171]. 14-3-3τ binding to the E2F transcription factor subunit E2F1 promotes E2F-mediated pro-apoptotic gene expression in response to DNA damage by preventing E2F1 degradation [172]. Following DNA damage, 14-3-3ε also promotes apoptosis by releasing the E2F subunit DP-2 [173]. Additionally, 14-3-3 binding regulates nuclear localization of RALT, a protein involved in the downregulation and endocytosis of ligand-activated EGFR and the chromatin targeting of c-Abl, and therefore its activity as a regulator of the DNA damage response to promote DNA repair [174]. 14-3-3 binding to RALT depends on Chk1-dependent S250 phosphorylation, slows down RALT trafficking to endosome-like structures, and stabilizes RALT protein [175].

The majority of DNA damage-induced 14-3-3 interactions seem to favor checkpoint activation or pro-apoptotic pathways, which contrasts with observations of 14-3-3 function under other stress and growth conditions as well as observations of 14-3-3ζ as a driver in cancer development (discussed above). It will be interesting to see whether the magnitude of DNA damage intensity or duration more generally influences the 14-3-3 stress response, as has been proposed for the 14-3-3-TAK1 interaction in switching from pro-survival to pro-apoptotic regimes with increasing stress. Likewise, contrasting observations on the effects of 14-3-3-induced MDMX localization following DNA damage may represent differences in DNA damaging agent, cell type, intensity of treatment, or specific 14-3-3 isoform involvement, which may also reflect a more dynamic influence of 14-3-3 in ensuring appropriate responses to environmental stimuli [159, 165–167]. 14-3-3 mediated responses may be more nuanced than simply promoting particular pathways; further experiments into time-, dose-, and agent- dependent 14-3-3 interactions will be needed to fully understand these processes, particularly how they play out in tumor-relevant cell types and microenvironments.

### Oxidative stress

Reactive oxygen species (ROS) can be produced through a variety of enzymatic and nonenzymatic mechanisms that include electron leakage from complex III of the electron transport chain, as well as chemotherapy and ionizing radiation. High levels of ROS impose a significant pro-apoptotic stress on a growing tumor. Thus cancer cells can acquire a variety of adaptations to survive oxidative stress, including the upregulation of anti-oxidant enzymes, NADPH metabolism, and other pro-survival signaling pathways, many of which are supported by 14-3-3 interactions (Fig. 4 and Table S2) [176–179]. Consistent with other cellular contexts, 14-3-3 interactions generally act as a brake on pro-apoptotic signaling downstream of oxidative stress through the mechanisms described below.

Although, as a whole, direct links between 14-3-3 and oxidative stress signaling are not quite as understood as other contexts (e.g., insulin, DNA damage), there are some clear points of convergence between 14-3-3 and oxidative stress pathways. The apoptosis signal-regulating kinase-1 (ASK1) responds to oxidative stress by activating the Jun N-terminal kinase (JNK) and p38 map kinase pathways, which results in caspase activation and apoptosis [180–183]. 14-3-3 interacts with ASK1 when phosphorylated at S967, which inhibits ASK1 kinase activity, likely by blocking access to or changing confirmation of the ASK1 active site [184–189]. Fu and colleagues showed that high levels of ROS trigger dephosphorylation of ASK1 at S967, which results in the release of 14-3-3 and activation of pro-apoptotic JNK- and P38 MAPK signaling [186]. The 14-3-3-ASK1 axis also integrates AKT signaling with downstream NF-κB, as AKT promotes IKK-mediated phosphorylation of ASK1 at S967 and the simultaneous activation of pro-survival NF-κB transcriptional pathways [189].

The stability of the 14-3-3-ASK1 interaction is a focus of multiple modes of regulation. Force and colleagues demonstrated that a member of the mammalian sterile 20 (Mst) kinase family, SOK-1, responds to high levels of ROS by phosphorylating 14-3-3ζ at S58, which triggers dissociation of 14-3-3ζ from ASK1 and results in activation of downstream pro-apoptotic pathways [190]. In addition, the 14-3-3ζ-ASK1 complex also interacts with thioredoxin, which may cooperate with 14-3-3ζ to inhibit ASK1 kinase activity [191]. An interesting mode of 14-3-3 redox regulation involves selenoprotein W (SelW), which may form a redox-regulated covalent interaction (Sec–Se–S–Cys bond) between a selenocysteine on SelW and an exposed cysteine on 14-3-3. This interaction is increased under conditions of high oxidative stress and disrupts 14-3-3 binding to ASK1. In this manner, SelW may act as a redox-regulated sink to disrupt pro-survival 14-3-3 interactions [192–194].

14-3-3 also inhibits ROS-induced cell death by interacting with FOXO transcription factors and preventing upregulation of a wide array of pro-apoptotic genes (described above in the context of insulin). Bonni and colleagues demonstrated that ROS-induced activation of another Mst family kinase, MST1, triggers phosphorylation of FOXO3 at S196 (nematode numbering, S207 in humans), which sits adjacent to the 14-3-3 binding site. This phosphorylation disrupts 14-3-3 binding to promote FOXO3-mediated cell death [195]. In addition to the 14-3-3-FOXO connection, many of the pro-survival 14-3-3 interactions with apoptotic effectors, which were identified under nutrient replete/normal redox conditions (discussed above), would also play roles in protecting cells from oxidative stress. Thus, through a wide array of PPIs, 14-3-3 acts as a brake that must be overcome, through release of 14-3-3 and/or sequestration in sinks (e.g., SelW), for oxidative stress-induced cell death to proceed.

## Conclusions and future perspectives

The inherently dynamic nature of 14-3-3s as phospho-binding proteins places them at the center of an adaptive signaling hub. As described above, our emerging understanding of 14-3-3 mechanisms suggest that the dynamic interactome of 14-3-3ζ, and likely other non-σ 14-3-3 isoforms, generally promotes a pro-survival cellular program appropriate for the condition at hand—whether that be insulin signaling, nutrient deprivation, oxidative stress, or chemotherapy-induced DNA damage. Although 14-3-3s do not fit the traditional mold of a drug target (i.e., they are not kinases, GPCRs, or ion channels), their position at the center of pro-growth/survival signaling hubs suggests the therapeutic potential for simultaneously disrupting many tumorigenic pathways. As an example, in breast cancer, where 14-3-3ζ is frequently overexpressed, the genetic ablation of 14-3-3ζ blocks the effects of key oncogenic drivers, including HER2 and PIK3CA, which are dependent on 14-3-3ζ for multiple downstream signaling pathways [13, 23, 196]. Accordingly, there are efforts to identify small molecule antagonists of 14-3-3ζ, although, as described above, there are still no 14-3-3 inhibitors in the clinic [42, 48, 50, 54].

In addition to the potential of 14-3-3s as therapeutic targets is their still largely-unexploited usefulness as probes to identify functional, stress-adaptive phosphorylations. The breadth of the phospho-proteome has grown exponentially with the development of highly sensitive MS methods. As of writing this manuscript, 285,896 non-redundant phosphorylations have been identified (phosphosite.org) and only a small fraction (<5%) have a known function. Furthermore, there is growing frustration among the PTM community that phosphorylations identified via global proteomics seldom have a measurable impact on protein function when looked at experimentally, which may be partly due to the promiscuous nature of some PTM-catalyzing enzymes, generating off-target PTMs that act as ‘red herrings’ in the search for functional PTMs [197]. This challenge can be minimized by using biological phospho-binding proteins, like 14-3-3s, as probes to identify functional phosphorylations. As described here, some of the most important mechanisms in cell signaling have been identified from the perspective of 14-3-3 binding. Going forward, it will be important to take advantage of unprecedented MS power to probe deeper into dynamic, stress-adaptive 14-3-3 interactomes and uncover phosphorylation-driven mechanisms that could be exploited therapeutically in cancer.

## Electronic supplementary material

Supplemental Table S1. Tab1

Supplemental Table S1. Tab2

Supplemental Table S1. Tab3

Supplemental Table S1. Tab4

Supplemental Table S2

Supplemental Table Legends

## References

[1] Knetsch ML, van Heusden GP, Ennis HL, Shaw DR, Epskamp SJ, Snaar-Jagalska BE (1997). Isolation of a Dictyostelium discoideum 14-3-3 homologue. Biochim Biophys Acta.

[2] Lalle M, Salzano AM, Crescenzi M, Pozio E (2006). The Giardia duodenalis 14-3-3 protein is post-translationally modified by phosphorylation and polyglycylation of the C-terminal tail. J Biol Chem.

[3] Mortenson JB, Heppler LN, Banks CJ, Weerasekara VK, Whited MD, Piccolo SR (2015). Histone deacetylase 6 (HDAC6) promotes the pro-survival activity of 14-3-3zeta via deacetylation of lysines within the 14-3-3zeta binding pocket. J Biol Chem.

[4] Choudhary C, Kumar C, Gnad F, Nielsen ML, Rehman M, Walther TC (2009). Lysine acetylation targets protein complexes and co-regulates major cellular functions. Science.

[5] Andersen JL, Thompson JW, Lindblom KR, Johnson ES, Yang CS, Lilley LR (2011). A biotin switch-based proteomics approach identifies 14-3-3zeta as a target of Sirt1 in the metabolic regulation of caspase-2. Mol Cell.

[6] Johnson C, Crowther S, Stafford MJ, Campbell DG, Toth R, MacKintosh C (2010). Bioinformatic and experimental survey of 14-3-3-binding sites. Biochem J.

[7] Chen S, Synowsky S, Tinti M, MacKintosh C (2011). The capture of phosphoproteins by 14-3-3 proteins mediates actions of insulin. Trends Endocrinol Metab.

[8] Gardino AK, Smerdon SJ, Yaffe MB (2006). Structural determinants of 14-3-3 binding specificities and regulation of subcellular localization of 14-3-3-ligand complexes: a comparison of the X-ray crystal structures of all human 14-3-3 isoforms. Semin Cancer Biol.

[9] Yaffe MB (2002). How do 14-3-3 proteins work?-- Gatekeeper phosphorylation and the molecular anvil hypothesis. FEBS Lett.

[10] Ottmann C, Marco S, Jaspert N, Marcon C, Schauer N, Weyand M (2007). Structure of a 14-3-3 coordinated hexamer of the plant plasma membrane H+ -ATPase by combining X-ray crystallography and electron cryomicroscopy. Mol Cell.

[11] Pollack JR, Sorlie T, Perou CM, Rees CA, Jeffrey SS, Lonning PE (2002). Microarray analysis reveals a major direct role of DNA copy number alteration in the transcriptional program of human breast tumors. Proc Natl Acad Sci USA.

[12] Garnis C, Coe BP, Ishkanian A, Zhang L, Rosin MP, Lam WL (2004). Novel regions of amplification on 8q distinct from the MYC locus and frequently altered in oral dysplasia and cancer. Genes Chromosomes Cancer.

[13] Lu J, Guo H, Treekitkarnmongkol W, Li P, Zhang J, Shi B (2009). 14-3-3zeta Cooperates with ErbB2 to promote ductal carcinoma in situ progression to invasive breast cancer by inducing epithelial-mesenchymal transition. Cancer Cell.

[14] Bergamaschi A, Christensen BL, Katzenellenbogen BS (2011). Reversal of endocrine resistance in breast cancer: interrelationships among 14-3-3zeta, FOXM1, and a gene signature associated with mitosis. Breast Cancer Res.

[15] Li Z, Zhao J, Du Y, Park HR, Sun SY, Bernal-Mizrachi L (2008). Down-regulation of 14-3-3zeta suppresses anchorage-independent growth of lung cancer cells through anoikis activation. Proc Natl Acad Sci USA.

[16] Matta A, Siu KW, Ralhan R (2012). 14-3-3 zeta as novel molecular target for cancer therapy. Expert Opin Ther Targets.

[17] Neal CL, Yu D (2010). 14-3-3zeta as a prognostic marker and therapeutic target for cancer. Expert Opin Ther Targets.

[18] Weerasekara VK, Panek DJ, Broadbent DG, Mortenson JB, Mathis AD, Logan GN (2014). Metabolic stress-induced rearrangement of the 14-3-3zeta interactome promotes autophagy via a ULK1- and AMPK-regulated 14-3-3zeta interaction with phosphorylated Atg9A. Mol Cell Biol.

[19] Xu J, Acharya S, Sahin O, Zhang Q, Saito Y, Yao J (2015). 14-3-3zeta turns TGF-beta’s function from tumor suppressor to metastasis promoter in breast cancer by contextual changes of Smad partners from p53 to Gli2. Cancer Cell.

[20] Yang X, Cao W, Zhang L, Zhang W, Zhang X, Lin H (2012). Targeting 14-3-3zeta in cancer therapy. Cancer Gene Ther.

[21] Li Y, Zou L, Li Q, Haibe-Kains B, Tian R, Li Y (2010). Amplification of LAPTM4B and YWHAZ contributes to chemotherapy resistance and recurrence of breast cancer. Nat Med.

[22] Litzenburger BC, Creighton CJ, Tsimelzon A, Chan BT, Hilsenbeck SG, Wang T (2011). High IGF-IR activity in triple-negative breast cancer cell lines and tumorgrafts correlates with sensitivity to anti-IGF-IR therapy. Clin Cancer Res.

[23] Rehman SK, Li SH, Wyszomierski SL, Wang Q, Li P, Sahin O (2014). 14-3-3zeta orchestrates mammary tumor onset and progression via miR-221-mediated cell proliferation. Cancer Res.

[24] Neal CL, Xu J, Li P, Mori S, Yang J, Neal NN (2012). Overexpression of 14-3-3zeta in cancer cells activates PI3K via binding the p85 regulatory subunit. Oncogene.

[25] Fan T, Li R, Todd NW, Qiu Q, Fang HB, Wang H (2007). Up-regulation of 14-3-3zeta in lung cancer and its implication as prognostic and therapeutic target. Cancer Res.

[26] Lin M, Morrison CD, Jones S, Mohamed N, Bacher J, Plass C (2009). Copy number gain and oncogenic activity of YWHAZ/14-3-3zeta in head and neck squamous cell carcinoma. Int J Cancer.

[27] Yang X, Cao W, Zhou J, Zhang W, Zhang X, Lin W (2011). 14-3-3zeta positive expression is associated with a poor prognosis in patients with glioblastoma. Neurosurgery.

[28] Neal CL, Yao J, Yang W, Zhou X, Nguyen NT, Lu J (2009). 14-3-3zeta overexpression defines high risk for breast cancer recurrence and promotes cancer cell survival. Cancer Res.

[29] Gu Y, Xu K, Torre C, Samur M, Barwick BG, Rupji M (2018). 14-3-3zeta binds the proteasome, limits proteolytic function and enhances sensitivity to proteasome inhibitors. Leukemia.

[30] Sinha P, Kohl S, Fischer J, Hutter G, Kern M, Kottgen E (2000). Identification of novel proteins associated with the development of chemoresistance in malignant melanoma using two-dimensional electrophoresis. Electrophoresis.

[31] Chuthapisith S, Layfield R, Kerr ID, Hughes C, Eremin O (2007). Proteomic profiling of MCF-7 breast cancer cells with chemoresistance to different types of anti-cancer drugs. Int J Oncol.

[32] Maxwell SA, Li Z, Jaye D, Ballard S, Ferrell J, Fu H (2009). 14-3-3zeta mediates resistance of diffuse large B cell lymphoma to an anthracycline-based chemotherapeutic regimen. J Biol Chem.

[33] Chatterjee D, Goldman M, Braastad CD, Darnowski J, Wyche JH, Pantazis P (2004). Reduction of 9-nitrocamptothecin-triggered apoptosis in DU-145 human prostate cancer cells by ectopic expression of 14-3-3zeta. Int J Oncol.

[34] Dong S, Kang S, Lonial S, Khoury HJ, Viallet J, Chen J (2008). Targeting 14-3-3 sensitizes native and mutant BCR-ABL to inhibition with U0126, rapamycin and Bcl-2 inhibitor GX15-070. Leukemia.

[35] Liou JY, Ghelani D, Yeh S, Wu KK (2007). Nonsteroidal anti-inflammatory drugs induce colorectal cancer cell apoptosis by suppressing 14-3-3epsilon. Cancer Res.

[36] Aeluri M, Chamakuri S, Dasari B, Guduru SK, Jimmidi R, Jogula S (2014). Small molecule modulators of protein-protein interactions: selected case studies. Chem Rev.

[37] Lo Conte L, Chothia C, Janin J (1999). The atomic structure of protein-protein recognition sites. J Mol Biol.

[38] Bradshaw JM, Mitaxov V, Waksman G (1999). Investigation of phosphotyrosine recognition by the SH2 domain of the Src kinase. J Mol Biol.

[39] Aghazadeh Y, Papadopoulos V (2016). The role of the 14-3-3 protein family in health, disease, and drug development. Drug Discov Today.

[40] Zhao J, Meyerkord CL, Du Y, Khuri FR, Fu H (2011). 14-3-3 proteins as potential therapeutic targets. Semin Cell Dev Biol.

[41] Ottmann C (2013). Small-molecule modulators of 14-3-3 protein-protein interactions. Bioorg Med Chem.

[42] Hartman AM, Hirsch AKH (2017). Molecular insight into specific 14-3-3 modulators: Inhibitors and stabilisers of protein-protein interactions of 14-3-3. Eur J Med Chem.

[43] Wang B, Yang H, Liu YC, Jelinek T, Zhang L, Ruoslahti E (1999). Isolation of high-affinity peptide antagonists of 14-3-3 proteins by phage display. Biochemistry.

[44] Petosa C, Masters SC, Bankston LA, Pohl J, Wang B, Fu H (1998). 14-3-3zeta binds a phosphorylated Raf peptide and an unphosphorylated peptide via its conserved amphipathic groove. J Biol Chem.

[45] Ottmann C, Yasmin L, Weyand M, Veesenmeyer JL, Diaz MH, Palmer RH (2007). Phosphorylation-independent interaction between 14-3-3 and exoenzyme S: from structure to pathogenesis. EMBO J.

[46] Masters SC, Fu H (2001). 14-3-3 proteins mediate an essential anti-apoptotic signal. J Biol Chem.

[47] Cao W, Yang X, Zhou J, Teng Z, Cao L, Zhang X (2010). Targeting 14-3-3 protein, difopein induces apoptosis of human glioma cells and suppresses tumor growth in mice. Apoptosis.

[48] Corradi V, Mancini M, Manetti F, Petta S, Santucci MA, Botta M (2010). Identification of the first non-peptidic small molecule inhibitor of the c-Abl/14-3-3 protein-protein interactions able to drive sensitive and Imatinib-resistant leukemia cells to apoptosis. Bioorg Med Chem Lett.

[49] Corradi V, Mancini M, Santucci MA, Carlomagno T, Sanfelice D, Mori M (2011). Computational techniques are valuable tools for the discovery of protein-protein interaction inhibitors: the 14-3-3sigma case. Bioorg Med Chem Lett.

[50] Mori M, Vignaroli G, Cau Y, Dinic J, Hill R, Rossi M (2014). Discovery of 14-3-3 protein-protein interaction inhibitors that sensitize multidrug-resistant cancer cells to doxorubicin and the Akt inhibitor GSK690693. ChemMedChem.

[51] Yan YM, Dai HQ, Du Y, Schneider B, Guo H, Li DP (2012). Identification of blapsins A and B as potent small-molecule 14-3-3 inhibitors from the insect Blaps japanensis. Bioorg Med Chem Lett.

[52] Du Y, Masters SC, Khuri FR, Fu H (2006). Monitoring 14-3-3 protein interactions with a homogeneous fluorescence polarization assay. J Biomol Screen.

[53] Thiel P, Roglin L, Meissner N, Hennig S, Kohlbacher O, Ottmann C (2013). Virtual screening and experimental validation reveal novel small-molecule inhibitors of 14-3-3 protein-protein interactions. Chem Commun (Camb).

[54] Zhao J, Du Y, Horton JR, Upadhyay AK, Lou B, Bai Y (2011). Discovery and structural characterization of a small molecule 14-3-3 protein-protein interaction inhibitor. Proc Natl Acad Sci USA.

[55] An SS, Askovich PS, Zarembinski TI, Ahn K, Peltier JM, von Rechenberg M (2011). A novel small molecule target in human airway smooth muscle for potential treatment of obstructive lung diseases: a staged high-throughput biophysical screening. Respir Res.

[56] Kobayashi H, Ogura Y, Sawada M, Nakayama R, Takano K, Minato Y (2011). Involvement of 14-3-3 proteins in the second epidermal growth factor-induced wave of Rac1 activation in the process of cell migration. J Biol Chem.

[57] Acevedo SF, Tsigkari KK, Grammenoudi S, Skoulakis EM (2007). In vivo functional specificity and homeostasis of Drosophila 14-3-3 proteins. Genetics.

[58] Schoenwaelder SM, Darbousset R, Cranmer SL, Ramshaw HS, Orive SL, Sturgeon S (2016). 14-3-3zeta regulates the mitochondrial respiratory reserve linked to platelet phosphatidylserine exposure and procoagulant function. Nat Commun.

[59] Yang HY, Wen YY, Chen CH, Lozano G, Lee MH (2003). 14-3-3 sigma positively regulates p53 and suppresses tumor growth. Mol Cell Biol.

[60] Ferguson AT, Evron E, Umbricht CB, Pandita TK, Chan TA, Hermeking H (2000). High frequency of hypermethylation at the 14-3-3 sigma locus leads to gene silencing in breast cancer. Proc Natl Acad Sci USA.

[61] Iwata N, Yamamoto H, Sasaki S, Itoh F, Suzuki H, Kikuchi T (2000). Frequent hypermethylation of CpG islands and loss of expression of the 14-3-3 sigma gene in human hepatocellular carcinoma. Oncogene.

[62] Suzuki H, Itoh F, Toyota M, Kikuchi T, Kakiuchi H, Imai K (2000). Inactivation of the 14-3-3 sigma gene is associated with 5’ CpG island hypermethylation in human cancers. Cancer Res.

[63] Neupane D, Korc M (2008). 14-3-3sigma Modulates pancreatic cancer cell survival and invasiveness. Clin Cancer Res.

[64] Nakamura T, Furukawa Y, Nakagawa H, Tsunoda T, Ohigashi H, Murata K (2004). Genome-wide cDNA microarray analysis of gene expression profiles in pancreatic cancers using populations of tumor cells and normal ductal epithelial cells selected for purity by laser microdissection. Oncogene.

[65] Logsdon CD, Simeone DM, Binkley C, Arumugam T, Greenson JK, Giordano TJ (2003). Molecular profiling of pancreatic adenocarcinoma and chronic pancreatitis identifies multiple genes differentially regulated in pancreatic cancer. Cancer Res.

[66] Berman HM, Westbrook J, Feng Z, Gilliland G, Bhat TN, Weissig H (2000). The Protein Data Bank. Nucleic Acids Res.

[67] Molzan M, Ottmann C (2012). Synergistic binding of the phosphorylated S233- and S259-binding sites of C-RAF to one 14-3-3zeta dimer. J Mol Biol.

[68] Gardino AK, Yaffe MB (2011). 14-3-3 proteins as signaling integration points for cell cycle control and apoptosis. Semin Cell Dev Biol.

[69] Wilker EW, Grant RA, Artim SC, Yaffe MB (2005). A structural basis for 14-3-3sigma functional specificity. J Biol Chem.

[70] Truong AB, Masters SC, Yang H, Fu H (2002). Role of the 14-3-3 C-terminal loop in ligand interaction. Proteins.

[71] Paul AL, Sehnke PC, Ferl RJ (2005). Isoform-specific subcellular localization among 14-3-3 proteins in Arabidopsis seems to be driven by client interactions. Mol Biol Cell.

[72] Testerink C, van der Meulen RM, Oppedijk BJ, de Boer AH, Heimovaara-Dijkstra S, Kijne JW (1999). Differences in spatial expression between 14-3-3 isoforms in germinating barley embryos. Plant Physiol.

[73] van Hemert MJ, Niemantsverdriet M, Schmidt T, Backendorf C, Spaink HP (2004). Isoform-specific differences in rapid nucleocytoplasmic shuttling cause distinct subcellular distributions of 14-3-3 sigma and 14-3-3 zeta. J Cell Sci.

[74] Qi W, Liu X, Qiao D, Martinez JD (2005). Isoform-specific expression of 14-3-3 proteins in human lung cancer tissues. Int J Cancer.

[75] Brunet A, Kanai F, Stehn J, Xu J, Sarbassova D, Frangioni JV (2002). 14-3-3 transits to the nucleus and participates in dynamic nucleocytoplasmic transport. J Cell Biol.

[76] Nutt LK, Buchakjian MR, Gan E, Darbandi R, Yoon SY, Wu JQ (2009). Metabolic control of oocyte apoptosis mediated by 14-3-3zeta-regulated dephosphorylation of caspase-2. Dev Cell.

[77] Collins BC, Gillet LC, Rosenberger G, Rost HL, Vichalkovski A, Gstaiger M (2013). Quantifying protein interaction dynamics by SWATH mass spectrometry: application to the 14-3-3 system. Nat Methods.

[78] Dubois F, Vandermoere F, Gernez A, Murphy J, Toth R, Chen S (2009). Differential 14-3-3 affinity capture reveals new downstream targets of phosphatidylinositol 3-kinase signaling. Mol Cell Proteomics.

[79] Margolis SS, Perry JA, Forester CM, Nutt LK, Guo Y, Jardim MJ (2006). Role for the PP2A/B56delta phosphatase in regulating 14-3-3 release from Cdc25 to control mitosis. Cell.

[80] DeYoung MP, Horak P, Sofer A, Sgroi D, Ellisen LW (2008). Hypoxia regulates TSC1/2-mTOR signaling and tumor suppression through REDD1-mediated 14-3-3 shuttling. Genes Dev.

[81] Sluchanko NN, Beelen S, Kulikova AA, Weeks SD, Antson AA, Gusev NB (2017). Structural basis for the interaction of a human small heat shock protein with the 14-3-3 universal signaling regulator. Structure.

[82] Tzivion G, Luo ZJ, Avruch J (2000). Calyculin A-induced vimentin phosphorylation sequesters 14-3-3 and displaces other 14-3-3 partners in vivo. J Biol Chem.

[83] Aitken A (2011). Post-translational modification of 14-3-3 isoforms and regulation of cellular function. Semin Cell Dev Biol.

[84] Chen YJ, Lu CT, Su MG, Huang KY, Ching WC, Yang HH (2015). dbSNO 2.0: a resource for exploring structural environment, functional and disease association and regulatory network of protein S-nitrosylation. Nucleic Acids Res.

[85] Hornbeck PV, Zhang B, Murray B, Kornhauser JM, Latham V (2015). Skrzypek E. PhosphoSitePlus, 2014: mutations, PTMs and recalibrations. Nucleic Acids Res.

[86] Chen C, Huang H, Wu CH (2017). Protein bioinformatics databases and resources. Methods Mol Biol.

[87] Krebs EG, Beavo JA (1979). Phosphorylation-dephosphorylation of enzymes. Annu Rev Biochem.

[88] Banks CJ, Rodriguez NW, Gashler KR, Pandya R, Mortenson JB, Whited MD (2017). Acylation of superoxide dismutase 1 (SOD1) at K122 governs SOD1-mediated inhibition of mitochondrial respiration. Mol Cell Biol.

[89] Dewhurst HM, Choudhury S, Torres MP (2015). Structural analysis of PTM hotspots (SAPH-ire)--a quantitative informatics method enabling the discovery of novel regulatory elements in protein families. Mol Cell Proteomics.

[90] Torres MP, Dewhurst H, Sundararaman N (2016). Proteome-wide Structural Analysis of PTM Hotspots Reveals Regulatory Elements Predicted to Impact Biological Function and Disease. Mol Cell Proteomics.

[91] Aghazadeh Y, Ye X, Blonder J, Papadopoulos V (2014). Protein modifications regulate the role of 14-3-3gamma adaptor protein in cAMP-induced steroidogenesis in MA-10 Leydig cells. J Biol Chem.

[92] Ellis JJ, Valencia TG, Zeng H, Roberts LD, Deaton RA, Grant SR (2003). CaM kinase IIdeltaC phosphorylation of 14-3-3beta in vascular smooth muscle cells: activation of class II HDAC repression. Mol Cell Biochem.

[93] Woodcock JM, Murphy J, Stomski FC, Berndt MC, Lopez AF (2003). The dimeric versus monomeric status of 14-3-3zeta is controlled by phosphorylation of Ser58 at the dimer interface. J Biol Chem.

[94] Ma Y, Pitson S, Hercus T, Murphy J, Lopez A, Woodcock J (2005). Sphingosine activates protein kinase A type II by a novel cAMP-independent mechanism. J Biol Chem.

[95] Woodcock JM, Ma Y, Coolen C, Pham D, Jones C, Lopez AF (2010). Sphingosine and FTY720 directly bind pro-survival 14-3-3 proteins to regulate their function. Cell Signal.

[96] Kanno T, Nishizaki T (2011). Sphingosine induces apoptosis in hippocampal neurons and astrocytes by activating caspase-3/-9 via a mitochondrial pathway linked to SDK/14-3-3 protein/Bax/cytochrome c. J Cell Physiol.

[97] Sluchanko NN, Artemova NV, Sudnitsyna MV, Safenkova IV, Antson AA, Levitsky DI (2012). Monomeric 14-3-3zeta has a chaperone-like activity and is stabilized by phosphorylated HspB6. Biochemistry.

[98] Sluchanko NN, Sudnitsyna MV, Chernik IS, Seit-Nebi AS, Gusev NB (2011). Phosphomimicking mutations of human 14-3-3zeta affect its interaction with tau protein and small heat shock protein HspB6. Arch Biochem Biophys.

[99] Aitken A, Baxter H, Dubois T, Clokie S, Mackie S, Mitchell K (2002). Specificity of 14-3-3 isoform dimer interactions and phosphorylation. Biochem Soc Trans.

[100] Dubois T, Rommel C, Howell S, Steinhussen U, Soneji Y, Morrice N (1997). 14-3-3 is phosphorylated by casein kinase I on residue 233. Phosphorylation at this site in vivo regulates Raf/14-3-3 interaction. J Biol Chem.

[101] Slone SR, Lavalley N, McFerrin M, Wang B, Yacoubian TA (2015). Increased 14-3-3 phosphorylation observed in Parkinson’s disease reduces neuroprotective potential of 14-3-3 proteins. Neurobiol Dis.

[102] Sluchanko NN, Chernik IS, Seit-Nebi AS, Pivovarova AV, Levitsky DI, Gusev NB (2008). Effect of mutations mimicking phosphorylation on the structure and properties of human 14-3-3zeta. Arch Biochem Biophys.

[103] Aitken A, Howell S, Jones D, Madrazo J, Patel Y (1995). 14-3-3 alpha and delta are the phosphorylated forms of raf-activating 14-3-3 beta and zeta. In vivo stoichiometric phosphorylation in brain at a Ser-Pro-Glu-Lys MOTIF. J Biol Chem.

[104] Tsuruta F, Sunayama J, Mori Y, Hattori S, Shimizu S, Tsujimoto Y (2004). JNK promotes Bax translocation to mitochondria through phosphorylation of 14-3-3 proteins. EMBO J.

[105] Sunayama J, Tsuruta F, Masuyama N, Gotoh Y (2005). JNK antagonizes Akt-mediated survival signals by phosphorylating 14-3-3. J Cell Biol.

[106] Zhang L, Wang H, Liu D, Liddington R, Fu H (1997). Raf-1 kinase and exoenzyme S interact with 14-3-3zeta through a common site involving lysine 49. J Biol Chem.

[107] Neukamm SS, Ott J, Dammeier S, Lehmann R, Haring HU, Schleicher E (2013). Phosphorylation of serine 1137/1138 of mouse insulin receptor substrate (IRS) 2 regulates cAMP-dependent binding to 14-3-3 proteins and IRS2 protein degradation. J Biol Chem.

[108] Chen Q, Xie B, Zhu S, Rong P, Sheng Y, Ducommun S (2017). A Tbc1d1 (Ser231Ala)-knockin mutation partially impairs AICAR- but not exercise-induced muscle glucose uptake in mice. Diabetologia.

[109] Chen Q, Quan C, Xie B, Chen L, Zhou S, Toth R (2014). GARNL1, a major RalGAP alpha subunit in skeletal muscle, regulates insulin-stimulated RalA activation and GLUT4 trafficking via interaction with 14-3-3 proteins. Cell Signal.

[110] Pehmoller C, Treebak JT, Birk JB, Chen S, Mackintosh C, Hardie DG (2009). Genetic disruption of AMPK signaling abolishes both contraction- and insulin-stimulated TBC1D1 phosphorylation and 14-3-3 binding in mouse skeletal muscle. Am J Physiol Endocrinol Metab.

[111] Yip MF, Ramm G, Larance M, Hoehn KL, Wagner MC, Guilhaus M (2008). CaMKII-mediated phosphorylation of the myosin motor Myo1c is required for insulin-stimulated GLUT4 translocation in adipocytes. Cell Metab.

[112] Holman GD, Sakamoto K (2008). Regulating the motor for GLUT4 vesicle traffic. Cell Metab.

[113] Datta SR, Katsov A, Hu L, Petros A, Fesik SW, Yaffe MB (2000). 14-3-3 proteins and survival kinases cooperate to inactivate BAD by BH3 domain phosphorylation. Mol Cell.

[114] Zha J, Harada H, Yang E, Jockel J, Korsmeyer SJ (1996). Serine phosphorylation of death agonist BAD in response to survival factor results in binding to 14-3-3 not BCL-X(L). Cell.

[115] Qi XJ, Wildey GM, Howe PH (2006). Evidence that Ser87 of BimEL is phosphorylated by Akt and regulates BimEL apoptotic function. J Biol Chem.

[116] Danial NN, Gramm CF, Scorrano L, Zhang CY, Krauss S, Ranger AM (2003). BAD and glucokinase reside in a mitochondrial complex that integrates glycolysis and apoptosis. Nature.

[117] Lizcano JM, Morrice N, Cohen P (2000). Regulation of BAD by cAMP-dependent protein kinase is mediated via phosphorylation of a novel site, Ser155. Biochem J.

[118] Nutt LK, Margolis SS, Jensen M, Herman CE, Dunphy WG, Rathmell JC (2005). Metabolic regulation of oocyte cell death through the CaMKII-mediated phosphorylation of caspase-2. Cell.

[119] Nellist M, Goedbloed MA, de Winter C, Verhaaf B, Jankie A, Reuser AJ (2002). Identification and characterization of the interaction between tuberin and 14-3-3zeta. J Biol Chem.

[120] Cai SL, Tee AR, Short JD, Bergeron JM, Kim J, Shen J (2006). Activity of TSC2 is inhibited by AKT-mediated phosphorylation and membrane partitioning. J Cell Biol.

[121] Gwinn DM, Shackelford DB, Egan DF, Mihaylova MM, Mery A, Vasquez DS (2008). AMPK phosphorylation of raptor mediates a metabolic checkpoint. Mol Cell.

[122] Vega-Rubin-de-Celis S, Abdallah Z, Kinch L, Grishin NV, Brugarolas J, Zhang X (2010). Structural analysis and functional implications of the negative mTORC1 regulator REDD1. Biochemistry.

[123] Favier FB, Costes F, Defour A, Bonnefoy R, Lefai E, Bauge S (2010). Downregulation of Akt/mammalian target of rapamycin pathway in skeletal muscle is associated with increased REDD1 expression in response to chronic hypoxia. Am J Physiol Regul Integr Comp Physiol.

[124] Pieri BL, Souza DR, Luciano TF, Marques SO, Pauli JR, Silva AS (2014). Effects of physical exercise on the P38MAPK/REDD1/14-3-3 pathways in the myocardium of diet-induced obesity rats. Horm Metab Res.

[125] Teo Z, Sng M K, Chan J S K, Lim M M K, Li Y, Li L, Phua T, Lee J Y H, Tan Z W, Zhu P, Tan N S (2017). Elevation of adenylate energy charge by angiopoietin-like 4 enhances epithelial–mesenchymal transition by inducing 14-3-3γ expression. Oncogene.

[126] Egan DF, Shackelford DB, Mihaylova MM, Gelino S, Kohnz RA, Mair W (2011). Phosphorylation of ULK1 (hATG1) by AMP-activated protein kinase connects energy sensing to mitophagy. Science.

[127] Bach M, Larance M, James DE, Ramm G (2011). The serine/threonine kinase ULK1 is a target of multiple phosphorylation events. Biochem J.

[128] Hu YL, DeLay M, Jahangiri A, Molinaro AM, Rose SD, Carbonell WS (2012). Hypoxia-induced autophagy promotes tumor cell survival and adaptation to antiangiogenic treatment in glioblastoma. Cancer Res.

[129] Song J, Qu Z, Guo X, Zhao Q, Zhao X, Gao L (2009). Hypoxia-induced autophagy contributes to the chemoresistance of hepatocellular carcinoma cells. Autophagy.

[130] Zhang H, Bosch-Marce M, Shimoda LA, Tan YS, Baek JH, Wesley JB (2008). Mitochondrial autophagy is an HIF-1-dependent adaptive metabolic response to hypoxia. J Biol Chem.

[131] Guo JY, Teng X, Laddha SV, Ma S, Van Nostrand SC, Yang Y (2016). Autophagy provides metabolic substrates to maintain energy charge and nucleotide pools in Ras-driven lung cancer cells. Genes Dev.

[132] Tarrado-Castellarnau M, de Atauri P, Cascante M (2016). Oncogenic regulation of tumor metabolic reprogramming. Oncotarget.

[133] Tang Y, Liu S, Li N, Guo W, Shi J, Yu H (2016). 14-3-3zeta promotes hepatocellular carcinoma venous metastasis by modulating hypoxia-inducible factor-1alpha. Oncotarget.

[134] Tang Y, Lv P, Sun Z, Han L, Luo B, Zhou W (2015). 14-3-3zeta up-regulates hypoxia-inducible factor-1alpha in hepatocellular carcinoma via activation of PI3K/Akt/NF-small ka, CyrillicB signal transduction pathway. Int J Clin Exp Pathol.

[135] Geng H, Harvey CT, Pittsenbarger J, Liu Q, Beer TM, Xue C (2011). HDAC4 protein regulates HIF1alpha protein lysine acetylation and cancer cell response to hypoxia. J Biol Chem.

[136] Furnari B, Rhind N, Russell P (1997). Cdc25 mitotic inducer targeted by chk1 DNA damage checkpoint kinase. Science.

[137] Rhind N, Furnari B, Russell P (1997). Cdc2 tyrosine phosphorylation is required for the DNA damage checkpoint in fission yeast. Genes Dev.

[138] Brown AL, Lee CH, Schwarz JK, Mitiku N, Piwnica-Worms H, Chung JH (1999). A human Cds1-related kinase that functions downstream of ATM protein in the cellular response to DNA damage. Proc Natl Acad Sci USA.

[139] Manke IA, Nguyen A, Lim D, Stewart MQ, Elia AE, Yaffe MB (2005). MAPKAP kinase-2 is a cell cycle checkpoint kinase that regulates the G2/M transition and S phase progression in response to UV irradiation. Mol Cell.

[140] Peng CY, Graves PR, Ogg S, Thoma RS, Byrnes MJ, Wu Z (1998). C-TAK1 protein kinase phosphorylates human Cdc25C on serine 216 and promotes 14-3-3 protein binding. Cell Growth Differ.

[141] Peng CY, Graves PR, Thoma RS, Wu Z, Shaw AS, Piwnica-Worms H (1997). Mitotic and G2 checkpoint control: regulation of 14-3-3 protein binding by phosphorylation of Cdc25C on serine-216. Science.

[142] Lopez-Girona A, Furnari B, Mondesert O, Russell P (1999). Nuclear localization of Cdc25 is regulated by DNA damage and a 14-3-3 protein. Nature.

[143] Zeng Y, Piwnica-Worms H (1999). DNA damage and replication checkpoints in fission yeast require nuclear exclusion of the Cdc25 phosphatase via 14-3-3 binding. Mol Cell Biol.

[144] Morris MC, Heitz A, Mery J, Heitz F, Divita G (2000). An essential phosphorylation-site domain of human cdc25C interacts with both 14-3-3 and cyclins. J Biol Chem.

[145] Dalal SN, Yaffe MB, DeCaprio JA (2004). 14-3-3 family members act coordinately to regulate mitotic progression. Cell Cycle.

[146] Dalal SN, Schweitzer CM, Gan J, DeCaprio JA (1999). Cytoplasmic localization of human cdc25C during interphase requires an intact 14-3-3 binding site. Mol Cell Biol.

[147] Kumagai A, Dunphy WG (1999). Binding of 14-3-3 proteins and nuclear export control the intracellular localization of the mitotic inducer Cdc25. Genes Dev.

[148] Margolis SS, Walsh S, Weiser DC, Yoshida M, Shenolikar S, Kornbluth S (2003). PP1 control of M phase entry exerted through 14-3-3-regulated Cdc25 dephosphorylation. EMBO J.

[149] Margolis SS, Perry JA, Weitzel DH, Freel CD, Yoshida M, Haystead TA (2006). A role for PP1 in the Cdc2/Cyclin B-mediated positive feedback activation of Cdc25. Mol Biol Cell.

[150] Bulavin DV, Higashimoto Y, Demidenko ZN, Meek S, Graves P, Phillips C (2003). Dual phosphorylation controls Cdc25 phosphatases and mitotic entry. Nat Cell Biol.

[151] Conklin DS, Galaktionov K, Beach D (1995). 14-3-3 proteins associate with cdc25 phosphatases. Proc Natl Acad Sci USA.

[152] Kasahara K, Goto H, Enomoto M, Tomono Y, Kiyono T, Inagaki M (2010). 14-3-3gamma mediates Cdc25A proteolysis to block premature mitotic entry after DNA damage. EMBO J.

[153] Jiang K, Pereira E, Maxfield M, Russell B, Goudelock DM, Sanchez Y (2003). Regulation of Chk1 includes chromatin association and 14-3-3 binding following phosphorylation on Ser-345. J Biol Chem.

[154] Wu ZH, Wong ET, Shi Y, Niu J, Chen Z, Miyamoto S (2010). ATM- and NEMO-dependent ELKS ubiquitination coordinates TAK1-mediated IKK activation in response to genotoxic stress. Mol Cell.

[155] Zuo S, Xue Y, Tang S, Yao J, Du R, Yang P (2010). 14-3-3 epsilon dynamically interacts with key components of mitogen-activated protein kinase signal module for selective modulation of the TNF-alpha-induced time course-dependent NF-kappaB activity. J Proteome Res.

[156] Tang S, Bao H, Zhang Y, Yao J, Yang P, Chen X (2013). 14-3-3epsilon mediates the cell fate decision-making pathways in response of hepatocellular carcinoma to Bleomycin-induced DNA damage. PLoS One.

[157] Waterman MJ, Stavridi ES, Waterman JL, Halazonetis TD (1998). ATM-dependent activation of p53 involves dephosphorylation and association with 14-3-3 proteins. Nat Genet.

[158] Rajagopalan S, Jaulent AM, Wells M, Veprintsev DB, Fersht AR (2008). 14-3-3 activation of DNA binding of p53 by enhancing its association into tetramers. Nucleic Acids Res.

[159] Wang YV, Leblanc M, Wade M, Jochemsen AG, Wahl GM (2009). Increased radioresistance and accelerated B cell lymphomas in mice with Mdmx mutations that prevent modifications by DNA-damage-activated kinases. Cancer Cell.

[160] Eischen CM (2017). Role of Mdm2 and Mdmx in DNA repair. J Mol Cell Biol.

[161] Zhou X, Cao B, Lu H (2017). Negative auto-regulators trap p53 in their web. J Mol Cell Biol.

[162] Okamoto K, Kashima K, Pereg Y, Ishida M, Yamazaki S, Nota A (2005). DNA damage-induced phosphorylation of MdmX at serine 367 activates p53 by targeting MdmX for Mdm2-dependent degradation. Mol Cell Biol.

[163] He G, Zhang YW, Lee JH, Zeng SX, Wang YV, Luo Z (2014). AMP-activated protein kinase induces p53 by phosphorylating MDMX and inhibiting its activity. Mol Cell Biol.

[164] Lee JH, Jin Y, He G, Zeng SX, Wang YV, Wahl GM (2012). Hypoxia activates tumor suppressor p53 by inducing ATR-Chk1 kinase cascade-mediated phosphorylation and consequent 14-3-3gamma inactivation of MDMX protein. J Biol Chem.

[165] Jin Y, Dai MS, Lu SZ, Xu Y, Luo Z, Zhao Y (2006). 14-3-3gamma binds to MDMX that is phosphorylated by UV-activated Chk1, resulting in p53 activation. EMBO J.

[166] LeBron C, Chen L, Gilkes DM, Chen J (2006). Regulation of MDMX nuclear import and degradation by Chk2 and 14-3-3. EMBO J.

[167] Pereg Y, Lam S, Teunisse A, Biton S, Meulmeester E, Mittelman L (2006). Differential roles of ATM- and Chk2-mediated phosphorylations of Hdmx in response to DNA damage. Mol Cell Biol.

[168] Merkel O, Taylor N, Prutsch N, Staber PB, Moriggl R, Turner SD (2017). When the guardian sleeps: Reactivation of the p53 pathway in cancer. Mutat Res.

[169] Chen X, Kim IK, Honaker Y, Paudyal SC, Koh WK, Sparks M (2015). 14-3-3 proteins restrain the Exo1 nuclease to prevent overresection. J Biol Chem.

[170] Cheruiyot A, Paudyal SC, Kim IK, Sparks M, Ellenberger T, Piwnica-Worms H (2015). Poly(ADP-ribose)-binding promotes Exo1 damage recruitment and suppresses its nuclease activities. DNA Repair (Amst).

[171] Andersen SD, Keijzers G, Rampakakis E, Engels K, Luhn P, El-Shemerly M (2012). 14-3-3 checkpoint regulatory proteins interact specifically with DNA repair protein human exonuclease 1 (hEXO1) via a semi-conserved motif. DNA Repair (Amst).

[172] Wang B, Liu K, Lin FT, Lin WC (2004). A role for 14-3-3 tau in E2F1 stabilization and DNA damage-induced apoptosis. J Biol Chem.

[173] Milton AH, Khaire N, Ingram L, O’Donnell AJ, La Thangue NB (2006). 14-3-3 proteins integrate E2F activity with the DNA damage response. EMBO J.

[174] Li C, Park S, Zhang X, Eisenberg LM, Zhao H, Darzynkiewicz Z (2017). Nuclear Gene 33/Mig6 regulates the DNA damage response through an ATM serine/threonine kinase-dependent mechanism. J Biol Chem.

[175] Takeda K, Takata T, Kawai Y, Ishigaki Y, Kajinami K (2013). Chk1-mediated phosphorylation of receptor-associated late transducer at serine 250 increases its stability by stimulating its interaction with 14-3-3. Genes Cells.

[176] Gill JG, Piskounova E, Morrison SJ (2016). Cancer, oxidative stress, and metastasis. Cold Spring Harb Symp Quant Biol.

[177] Papa L, Hahn M, Marsh EL, Evans BS, Germain D (2014). SOD2 to SOD1 switch in breast cancer. J Biol Chem.

[178] Fan J, Ye J, Kamphorst JJ, Shlomi T, Thompson CB, Rabinowitz JD (2014). Quantitative flux analysis reveals folate-dependent NADPH production. Nature.

[179] Zhao T, Zhu Y, Morinibu A, Kobayashi M, Shinomiya K, Itasaka S (2014). HIF-1-mediated metabolic reprogramming reduces ROS levels and facilitates the metastatic colonization of cancers in lungs. Sci Rep.

[180] Saitoh M, Nishitoh H, Fujii M, Takeda K, Tobiume K, Sawada Y (1998). Mammalian thioredoxin is a direct inhibitor of apoptosis signal-regulating kinase (ASK) 1. EMBO J.

[181] Nishitoh H, Matsuzawa A, Tobiume K, Saegusa K, Takeda K, Inoue K (2002). ASK1 is essential for endoplasmic reticulum stress-induced neuronal cell death triggered by expanded polyglutamine repeats. Genes Dev.

[182] Tobiume K, Matsuzawa A, Takahashi T, Nishitoh H, Morita K, Takeda K (2001). ASK1 is required for sustained activations of JNK/p38 MAP kinases and apoptosis. EMBO Rep.

[183] Ichijo H, Nishida E, Irie K, ten Dijke P, Saitoh M, Moriguchi T (1997). Induction of apoptosis by ASK1, a mammalian MAPKKK that activates SAPK/JNK and p38 signaling pathways. Science.

[184] Petrvalska O, Kosek D, Kukacka Z, Tosner Z, Man P, Vecer J (2016). Structural Insight into the 14-3-3 Protein-dependent Inhibition of Protein Kinase ASK1 (Apoptosis Signal-regulating kinase 1). J Biol Chem.

[185] Cockrell LM, Puckett MC, Goldman EH, Khuri FR, Fu H (2010). Dual engagement of 14-3-3 proteins controls signal relay from ASK2 to the ASK1 signalosome. Oncogene.

[186] Goldman EH, Chen L, Fu H (2004). Activation of apoptosis signal-regulating kinase 1 by reactive oxygen species through dephosphorylation at serine 967 and 14-3-3 dissociation. J Biol Chem.

[187] Zhang L, Chen J, Fu H (1999). Suppression of apoptosis signal-regulating kinase 1-induced cell death by 14-3-3 proteins. Proc Natl Acad Sci USA.

[188] Seong HA, Jung H, Ichijo H, Ha H (2010). Reciprocal negative regulation of PDK1 and ASK1 signaling by direct interaction and phosphorylation. J Biol Chem.

[189] Puckett MC, Goldman EH, Cockrell LM, Huang B, Kasinski AL, Du Y (2013). Integration of apoptosis signal-regulating kinase 1-mediated stress signaling with the Akt/protein kinase B-IkappaB kinase cascade. Mol Cell Biol.

[190] Zhou J, Shao Z, Kerkela R, Ichijo H, Muslin AJ, Pombo C (2009). Serine 58 of 14-3-3zeta is a molecular switch regulating ASK1 and oxidant stress-induced cell death. Mol Cell Biol.

[191] Noguchi T, Takeda K, Matsuzawa A, Saegusa K, Nakano H, Gohda J (2005). Recruitment of tumor necrosis factor receptor-associated factor family proteins to apoptosis signal-regulating kinase 1 signalosome is essential for oxidative stress-induced cell death. J Biol Chem.

[192] Dikiy A, Novoselov SV, Fomenko DE, Sengupta A, Carlson BA, Cerny RL (2007). SelT, SelW, SelH, and Rdx12: genomics and molecular insights into the functions of selenoproteins of a novel thioredoxin-like family. Biochemistry.

[193] Jeon YH, Ko KY, Lee JH, Park KJ, Jang JK, Kim IY (2016). Identification of a redox-modulatory interaction between selenoprotein W and 14-3-3 protein. Biochim Biophys Acta.

[194] Jeon YH, Park YH, Kwon JH, Lee JH, Kim IY (2013). Inhibition of 14-3-3 binding to Rictor of mTORC2 for Akt phosphorylation at Ser473 is regulated by selenoprotein W. Biochim Biophys Acta.

[195] Lehtinen MK, Yuan Z, Boag PR, Yang Y, Villen J, Becker EB (2006). A conserved MST-FOXO signaling pathway mediates oxidative-stress responses and extends life span. Cell.

[196] Joshi S, Yang J, Wang Q, Li P, Wang H, Zhang Q (2017). 14-3-3zeta loss impedes oncogene-induced mammary tumorigenesis and metastasis by attenuating oncogenic signaling. Am J Cancer Res.

[197] Lienhard GE (2008). Non-functional phosphorylations?. Trends Biochem Sci.

