# Supplementary material for: The dynamic and stress-adaptive signaling hub of 14-3-3: emerging mechanisms of regulation and context-dependent protein–protein interactions
Source: Oncogene. 2018 Jun 18;37(42):5587–604. doi: 10.1038/s41388-018-0348-3 (PMC6193947; doi:10.1038/s41388-018-0348-3)
Supplement: Supplementary file 1 — Supplemental Table S1. Tab1 [file 41388_2018_348_MOESM1_ESM.pdf]

Category	Column	Description
SAPH-ire	RANK	MAP rank order based on SAPH-ire FPx
SAPH-ire	MAP	Modified Alignment Position
SAPH-ire	Family	Interpro Family ID
SAPH-ire	Mem	Number of UIDs within the family alignment
SAPH-ire	AP	Alignment Position
SAPH-ire	MAP PTMs	List of PTM types found in the MAP
SAPH-ire	PTMc	Number of members with observed modification within the MAP
SAPH-ire	PTMc/Mem	PTMc normalized as a function of total members within the MAP (gaps excluded)
SAPH-ire	OBSrc	observation count (number of observations from literature and high throughput proteomics
SAPH-ire	PRC	PTM residue conservation
SAPH-ire	DIS	Disorder tendancy (from IUPred)
SAPH-ire	Nc	Neighbor Count (number of MAPs within a +/-2 alignment position window
SAPH-ire	KNc	Known Neighbor Count (number of MAPs with at least one known functional PTM within +/- 2 alignment position window)
SAPH-ire	NPTMc	Neighbor PTM count (count of PTMs observed within a +/-2 alignment position window)
SAPH-ire	OIDc	Sum of unique organism UIDs within the MAP
SAPH-ire	Cc	Sum of PTMs observed within +/- 2 alignment position window (including the MAP in question)
SAPH-ire	Known	Binary classifier of known functional status of a MAP
SAPH-ire	KFSC	Known Function Source Count (sum of PMID's demonstrating funcitonal impact for any PTM within the MAP)
SAPH-ire	SAPH-ireFPx Score (rel to Family Max)	Probability score from SAPH-ire (Normalized to the family maximum value)
PTM Data	P63104 Aligned Native Position	The aligned native position of P63104 within the MAP
PTM Data	P63104 Resi	Residue of P63104
PTM Data	P63104 PTM	PTM type for P63104 at this site (may be redundant for multiple PTMs observed on the same residue)
PTM Data	Database	PTM database
PTM Data	Reference	Reference data (if available)
Functional Data	Functional in P63104?	PTM of P63104 has been shown to be functional
Functional Data	P63104 Functional Native Position	PSP-curated data for known funciton
Functional Data	P63104 Functional PTM	PSP-curated data for known funciton
Functional Data	GENE	PSP-curated data for known funciton
Functional Data	PROTEIN	PSP-curated data for known funciton
Functional Data	SITE_+/-7_AA	PSP-curated data for known funciton
Functional Data	DOMAIN	PSP-curated data for known funciton
Functional Data	ON_FUNCTION	PSP-curated data for known funciton
Functional Data	ON_PROCESS	PSP-curated data for known funciton
Functional Data	ON_PROT_INTERACT	PSP-curated data for known funciton
Functional Data	ON_OTHER_INTERACT	PSP-curated data for known funciton
Functional Data	PMIDs	PSP-curated data for known funciton
