# Supplementary material for: The dynamic and stress-adaptive signaling hub of 14-3-3: emerging mechanisms of regulation and context-dependent protein–protein interactions
Source: Oncogene. 2018 Jun 18;37(42):5587–604. doi: 10.1038/s41388-018-0348-3 (PMC6193947; doi:10.1038/s41388-018-0348-3)
Supplement: Supplementary file 2 — Supplemental Table S1. Tab2 [file 41388_2018_348_MOESM2_ESM.pdf]

RANK	MAP	Family	Mem	AP	MAP PTMs	PTMc	PTMc/Mem	OBSrc	PRC	DIS	Nc	KNc	NPTMc	OIDc	Cc	Known	KFSC	SAPH-ireFPx Score (rel to Family Max)	P63104 Aligned Native Position	P63104 Resi	P63104 PTM	Database	Reference	Functional in P63104?	P63104 Functional Native Position	P63104 Functional PTM	GENE	PROTEIN	SITE_+/-7_AA	DOMAIN	ON_FUNCTION	ON_PROCESS	ON_PROT_INTERACT	ON_OTHER_INTERACT	PMIDs
1	IPR000308-137	IPR000308	37	137	Ubiquitination;Acetylation;Ubiquitination;Ubiquitination;Ubiquitination;Ubiquitination;Acetylation;Ubiquitination;Ubiquitination;Ubiquitination;Acetylation;Ubiquitination;Ubiquitination;Ubiquitination;Ubiquitination;Ubiquitination;Ubiquitination;Ubiquitination;Acetylation	17	0.459459459	95	1	0.08702703	1	1	32	12	49	0	0	100	P63104-122	K	Ubiquitination	PSP	NULL												
2	IPR000308-59	IPR000308	37	59	Acetylation;Ubiquitination;Acetylation;Ubiquitination;Acetylation;Methylation;Ubiquitination;Acetylation;Ubiquitination;Ubiquitination;Ubiquitination;Acetylation;Methylation;Ubiquitination;Acetylation;Ubiquitination;Acetylation;Ubiquitination;Acetylation;Methylation;Ubiquitination;Acetylation;Ubiquitination;Acetylation;Ubiquitination;Acetylation;Methylation;Ubiquitination;Acetylation;Ubiquitination;Acetylation;Ubiquitination;Acetylation;Ubiquitination	16	0.432432432	816	1	0.25081081	1	0	14	12	30	1	4	82.71092193	P63104-49	K	Acetylation	PSP	NULL												
2	IPR000308-59	IPR000308	37	59	Acetylation;Ubiquitination;Acetylation;Ubiquitination;Acetylation;Methylation;Ubiquitination;Acetylation;Ubiquitination;Ubiquitination;Ubiquitination;Acetylation;Methylation;Ubiquitination;Acetylation;Ubiquitination;Acetylation;Ubiquitination;Acetylation;Methylation;Ubiquitination;Acetylation;Ubiquitination;Acetylation;Ubiquitination;Acetylation;Methylation;Ubiquitination;Acetylation;Ubiquitination;Acetylation;Ubiquitination;Acetylation;Ubiquitination	16	0.432432432	816	1	0.25081081	1	0	14	12	30	1	4	82.71092193	P63104-49	K	Methylation	PSP	NULL												
2	IPR000308-59	IPR000308	37	59	Acetylation;Ubiquitination;Acetylation;Ubiquitination;Acetylation;Methylation;Ubiquitination;Acetylation;Ubiquitination;Ubiquitination;Ubiquitination;Acetylation;Methylation;Ubiquitination;Acetylation;Ubiquitination;Acetylation;Ubiquitination;Acetylation;Methylation;Ubiquitination;Acetylation;Ubiquitination;Acetylation;Ubiquitination;Acetylation;Methylation;Ubiquitination;Acetylation;Ubiquitination;Acetylation;Ubiquitination;Acetylation;Ubiquitination	16	0.432432432	816	1	0.25081081	1	0	14	12	30	1	4	82.71092193	P63104-49	K	Ubiquitination	PSP	NULL												
3	IPR000308-58	IPR000308	37	58	Phosphorylation;Phosphorylation;Phosphorylation;Phosphorylation;Phosphorylation;Phosphorylation;Phosphorylation;Phosphorylation;Phosphorylation;Phosphorylation;Phosphorylation;Phosphorylation;Phosphorylation;Phosphorylation	14	0.378378378	57	1	0.22972973	1	1	34	12	48	0	0	75.11299543	P63104-48	Y	Phosphorylation	PSP	NULL												
4	IPR000308-135	IPR000308	37	135	Acetylation;Ubiquitination;Acetylation;Ubiquitination;Acetylation;Ubiquitination;Acetylation;Ubiquitination;Acetylation;Ubiquitination;Acetylation;Ubiquitination;Ubiquitination;Acetylation;Ubiquitination;Acetylation;Ubiquitination;Acetylation;Ubiquitination;Acetylation;Ubiquitination;Acetylation;Ubiquitination;Acetylation;Ubiquitination;Acetylation;Ubiquitination;Acetylation;Ubiquitination;Acetylation;Ubiquitination;Acetylation	17	0.459459459	471	1	0.08891892	3	0	22	12	39	1	2	68.6655591	P63104-120	K	Acetylation	PSP	NULL												
4	IPR000308-135	IPR000308	37	135	Acetylation;Ubiquitination;Acetylation;Ubiquitination;Acetylation;Ubiquitination;Acetylation;Ubiquitination;Acetylation;Ubiquitination;Acetylation;Ubiquitination;Ubiquitination;Acetylation;Ubiquitination;Acetylation;Ubiquitination;Acetylation;Ubiquitination;Acetylation;Ubiquitination;Acetylation;Ubiquitination;Acetylation;Ubiquitination;Acetylation;Ubiquitination;Acetylation;Ubiquitination;Acetylation;Ubiquitination;Acetylation	17	0.459459459	471	1	0.08891892	3	0	22	12	39	1	2	68.6655591	P63104-120	K	Ubiquitination	PSP	NULL												
5	IPR000308-55	IPR000308	37	55	Phosphorylation;Phosphorylation;Phosphorylation;Phosphorylation;Phosphorylation;Phosphorylation;Phosphorylation;Phosphorylation;Phosphorylation;Phosphorylation;Phosphorylation;Phosphorylation;Phosphorylation;Phosphorylation;Phosphorylation;Phosphorylation;Phosphorylation;Phosphorylation	18	0.486486486	178	1	0.19675676	0	0	0	12	18	0	0	54.41778659	P63104-45	S	Phosphorylation	PSP	NULL												
6	IPR000308-74	IPR000308	37	74	Phosphorylation;Phosphorylation;Phosphorylation;Phosphorylation;Phosphorylation;Phosphorylation;Phosphorylation;Phosphorylation;Phosphorylation;Phosphorylation;Phosphorylation;Phosphorylation;Phosphorylation;Phosphorylation;Phosphorylation;Phosphorylation	16	0.432432432	27	1	0.33945946	1	1	11	12	27	0	0	53.25446527	P63104-64	S	Phosphorylation	PSP	NULL												
7	IPR000308-78	IPR000308	37	78	Acetylation;Ubiquitination;Acetylation;Ubiquitination;Acetylation;Ubiquitination;Ubiquitination;Ubiquitination;Acetylation;Ubiquitination;Acetylation;Ubiquitination;Ubiquitination;Acetylation;Acetylation;Ubiquitination;Ubiquitination;Acetylation;Ubiquitination;Acetylation;Ubiquitination;Acetylation;Ubiquitination;Acetylation;Ubiquitination;Acetylation	16	0.432432432	107	1	0.37108108	2	1	4	12	20	0	0	52.01179587	P63104-68	K	Acetylation	PSP	NULL												
7	IPR000308-78	IPR000308	37	78	Acetylation;Ubiquitination;Acetylation;Ubiquitination;Acetylation;Ubiquitination;Ubiquitination;Ubiquitination;Acetylation;Ubiquitination;Acetylation;Ubiquitination;Ubiquitination;Acetylation;Acetylation;Ubiquitination;Ubiquitination;Acetylation;Ubiquitination;Acetylation;Ubiquitination;Acetylation;Ubiquitination;Acetylation;Ubiquitination;Acetylation	16	0.432432432	107	1	0.37108108	2	1	4	12	20	0	0	52.01179587	P63104-68	K	Ubiquitination	PSP	NULL												
8	IPR000308-129	IPR000308	37	129	Phosphorylation;Phosphorylation;Phosphorylation;Phosphorylation;Phosphorylation;Phosphorylation;Phosphorylation;Phosphorylation;Phosphorylation;Phosphorylation;Phosphorylation	11	0.297297297	16	1	0.08694444	2	1	24	12	35	0	0	51.89291494	P63104-114	S	Phosphorylation	PSP	NULL												
9	IPR000308-19	IPR000308	37	19	Acetylation;Ubiquitination;Acetylation;Ubiquitination;Ubiquitination;Ubiquitination;Ubiquitination;Acetylation;Ubiquitination;Acetylation;Ubiquitination;Acetylation;Ubiquitination;Acetylation;Ubiquitination;Acetylation;Ubiquitination;Acetylation;Ubiquitination;Ubiquitination;Acetylation;Ubiquitination	13	0.351351351	190	0.86486486	0.37756757	1	0	14	12	27	0	0	51.58024163	P63104-11	K	Acetylation	PSP	NULL												
9	IPR000308-19	IPR000308	37	19	Acetylation;Ubiquitination;Acetylation;Ubiquitination;Ubiquitination;Ubiquitination;Ubiquitination;Acetylation;Ubiquitination;Acetylation;Ubiquitination;Acetylation;Ubiquitination;Acetylation;Ubiquitination;Acetylation;Ubiquitination;Acetylation;Ubiquitination;Ubiquitination;Acetylation;Ubiquitination	13	0.351351351	190	0.86486486	0.37756757	1	0	14	12	27	0	0	51.58024163	P63104-11	K	Ubiquitination	PSP	NULL												
10	IPR000308-130	IPR000308	37	130	Ubiquitination;Acetylation;Ubiquitination;Acetylation;Ubiquitination;Acetylation;Ubiquitination;Acetylation;Ubiquitination;Acetylation;Acetylation;Ubiquitination;Acetylation;Ubiquitination;Acetylation;Ubiquitination;Acetylation;Ubiquitination;Acetylation;Ubiquitination;Acetylation	13	0.351351351	181	0.88888889	0.11694444	1	0	11	12	24	1	2	51.57366248	P63104-115	K	Acetylation	PSP	NULL												
10	IPR000308-130	IPR000308	37	130	Ubiquitination;Acetylation;Ubiquitination;Acetylation;Ubiquitination;Acetylation;Ubiquitination;Acetylation;Ubiquitination;Acetylation;Acetylation;Ubiquitination;Acetylation;Ubiquitination;Acetylation;Ubiquitination;Acetylation;Ubiquitination;Acetylation;Ubiquitination;Acetylation	13	0.351351351	181	0.88888889	0.11694444	1	0	11	12	24	1	2	51.57366248	P63104-115	K	Ubiquitination	PSP	NULL												
11	IPR000308-143	IPR000308	37	143	Phosphorylation;Phosphorylation;Phosphorylation;Phosphorylation;Phosphorylation;Phosphorylation;Phosphorylation;Phosphorylation;Phosphorylation;Phosphorylation;Phosphorylation	11	0.297297297	30	1	0.11486486	3	1	11	12	22	0	0	51.25691731	P63104-128	Y	Phosphorylation	PSP	NULL												

12	IPR000308-68	IPR000308	37	68	Phosphorylation;Phosphorylation;Phosphorylation;Phosphorylation;Phosphorylation;Phosphorylation;Phosphorylation;Phosphorylation;Phosphorylation;Phosphorylation;Phosphorylation	11	0.297297297	55	0.89189189	0.19	1	0	4	12	15	1	10	51.14959927	P63104-58	S	Phosphorylation	PSP	NULL	YES	P63104-58	p	YWHAZ	14-3-3 zeta	VVGARRSsWRVVSSI	14-3-3	protein degradation; molecular association, regulation	apoptosis, altered	14-3-3 zeta(DISRUPTS); 14-3-3 zeta(INDUCES); Tau iso3(DISRUPTS); ASK1(DISRUPTS); 14-3-3 epsilon(DISRUPTS); HSP20(DISRUPTS); p53(DISRUPTS)	11956222; 16376338; 18559254; 19451227; 21081103
13	IPR000308-172	IPR000308	37	172	Acetylation;Ubiquitination;Acetylation;Acetylation;Ubiquitination;Ubiquitination;Ubiquitination;Acetylation;Ubiquitination;Acetylation;Acetylation;Phosphorylation;Acetylation;Ubiquitination;Acetylation;Ubiquitination;Acetylation;Ubiquitination	12	0.324324324	62	0.7027027	0.34108108	2	0	8	12	20	0	0	51.0908373	P63104-157	K	Acetylation	PSP	NULL											
13	IPR000308-172	IPR000308	37	172	Acetylation;Ubiquitination;Acetylation;Acetylation;Ubiquitination;Ubiquitination;Ubiquitination;Acetylation;Ubiquitination;Acetylation;Acetylation;Phosphorylation;Acetylation;Ubiquitination;Acetylation;Ubiquitination;Acetylation;Ubiquitination	12	0.324324324	62	0.7027027	0.34108108	2	0	8	12	20	0	0	51.0908373	P63104-157	K	Ubiquitination	PSP	NULL											
14	IPR000308-245	IPR000308	37	245	Phosphorylation;Phosphorylation;Phosphorylation;Phosphorylation;Phosphorylation;Phosphorylation;Phosphorylation;Phosphorylation;Phosphorylation;Phosphorylation;Phosphorylation	11	0.297297297	23	0.94594595	0.41945946	2	1	12	12	23	0	0	50.99886488	P63104-230	S	Phosphorylation	PSP	NULL											
15	IPR000308-47	IPR000308	37	47	Phosphorylation;Phosphorylation;Phosphorylation;Phosphorylation;Phosphorylation;Phosphorylation;Phosphorylation;Phosphorylation;Phosphorylation;Phosphorylation	10	0.27027027	22	1	0.27621622	1	0	1	12	11	0	0	50.79838111	P63104-37	S	Phosphorylation	PSP	NULL											
16	IPR000308-73	IPR000308	37	73	Phosphorylation;Phosphorylation;Phosphorylation;Phosphorylation;Phosphorylation;Phosphorylation;Phosphorylation;Phosphorylation;Phosphorylation;Phosphorylation;Phosphorylation	11	0.297297297	15	1	0.34648649	1	0	16	12	27	1	1	50.43670447	P63104-63	S	Phosphorylation	PSP	NULL											
17	IPR000308-208	IPR000308	37	208	Ubiquitination;Ubiquitination;Ubiquitination;Acetylation;Ubiquitination;Ubiquitination;Ubiquitination;Ubiquitination;Ubiquitination;Ubiquitination	9	0.243243243	61	1	0.21444444	1	0	2	12	11	0	0	50.31878836	P63104-193	K	Ubiquitination	PSP	NULL											
18	IPR000308-149	IPR000308	37	149	Phosphorylation;Phosphorylation;S-Nitrosylation;Phosphorylation;Phosphorylation;Phosphorylation;Phosphorylation;Phosphorylation;S-Nitrosylation;Phosphorylation	10	0.27027027	14	0.81081081	0.22189189	0	0	0	12	10	0	0	48.7848573	P63104-134	A														
19	IPR000308-17	IPR000308	37	17	Ubiquitination;Acetylation;Ubiquitination;Acetylation;Ubiquitination;Ubiquitination;Ubiquitination;Ubiquitination;Acetylation;Ubiquitination;Ubiquitination;Acetylation;Ubiquitination;Ubiquitination	10	0.27027027	87	0.51351351	0.45189189	2	0	23	12	33	0	0	48.77784619	P63104-9	K	Acetylation	PSP	NULL											
19	IPR000308-17	IPR000308	37	17	Ubiquitination;Acetylation;Ubiquitination;Acetylation;Ubiquitination;Ubiquitination;Ubiquitination;Ubiquitination;Acetylation;Ubiquitination;Ubiquitination;Acetylation;Ubiquitination;Ubiquitination	10	0.27027027	87	0.51351351	0.45189189	2	0	23	12	33	0	0	48.77784619	P63104-9	K	Ubiquitination	PSP	NULL											
20	IPR000308-11	IPR000308	37	11	Acetylation;Acetylation;Methylation;Ubiquitination;Acetylation;Acetylation;Acetylation;Ubiquitination;Acetylation;Methylation;Acetylation;Ubiquitination;Acetylation	9	0.243243243	50	0.97297297	0.7	3	0	7	12	16	0	0	46.95547999	P63104-3	K	Acetylation	PSP	NULL											
20	IPR000308-11	IPR000308	37	11	Acetylation;Acetylation;Methylation;Ubiquitination;Acetylation;Acetylation;Acetylation;Ubiquitination;Acetylation;Methylation;Acetylation;Ubiquitination;Acetylation	9	0.243243243	50	0.97297297	0.7	3	0	7	12	16	0	0	46.95547999	P63104-3	K	Methylation	PSP	NULL											
20	IPR000308-11	IPR000308	37	11	Acetylation;Acetylation;Methylation;Ubiquitination;Acetylation;Acetylation;Acetylation;Ubiquitination;Acetylation;Methylation;Acetylation;Ubiquitination;Acetylation	9	0.243243243	50	0.97297297	0.7	3	0	7	12	16	0	0	46.95547999	P63104-3	K	Ubiquitination	PSP	NULL											
21	IPR000308-226	IPR000308	37	226	Phosphorylation;Phosphorylation;Phosphorylation;Phosphorylation;Phosphorylation;Phosphorylation;Phosphorylation;Phosphorylation;Phosphorylation	9	0.243243243	17	1	0.24324324	2	0	11	12	20	0	0	45.82260482	P63104-211	Y	Phosphorylation	PSP	NULL											
22	IPR000308-127	IPR000308	37	127	Phosphorylation;Phosphorylation;Phosphorylation	3	0.081081081	44	0.8	0.02	2	0	12	3	15	0	0	45.69606555																
23	IPR000308-87	IPR000308	37	87	Ubiquitination;Acetylation;Ubiquitination;Acetylation;Ubiquitination;Acetylation;Ubiquitination;Ubiquitination;Ubiquitination;Ubiquitination	9	0.243243243	14	0.54054054	0.41810811	2	1	10	12	19	0	0	45.04576116	P63104-74	K	Acetylation	PSP	NULL											
24	IPR000308-225	IPR000308	37	225	Phosphorylation;Phosphorylation;Phosphorylation;Phosphorylation;Phosphorylation;Phosphorylation;Phosphorylation;Phosphorylation;Phosphorylation	9	0.243243243	9	1	0.21864865	2	0	11	12	20	0	0	40.11288082	P63104-210	S	Phosphorylation	PSP	NULL											
25	IPR000308-154	IPR000308	37	154	Acetylation;Ubiquitination;Ubiquitination;Acetylation;Ubiquitination;Acetylation;Ubiquitination;Acetylation;Ubiquitination;Acetylation;Ubiquitination;Ubiquitination;Acetylation;Ubiquitination	9	0.243243243	105	0.54054054	0.35351351	4	1	22	12	31	0	0	37.98679236	P63104-139	K	Acetylation	PSP	NULL											
25	IPR000308-154	IPR000308	37	154	Acetylation;Ubiquitination;Ubiquitination;Acetylation;Ubiquitination;Acetylation;Ubiquitination;Acetylation;Ubiquitination;Acetylation;Ubiquitination;Ubiquitination;Acetylation;Ubiquitination	9	0.243243243	105	0.54054054	0.35351351	4	1	22	12	31	0	0	37.98679236	P63104-139	K	Ubiquitination	PSP	NULL											
26	IPR000308-199	IPR000308	37	199	Phosphorylation;Phosphorylation;Phosphorylation;Phosphorylation;Phosphorylation;Phosphorylation;Phosphorylation;Phosphorylation	8	0.216216216	23	0.75675676	0.04081081	0	0	0	12	8	1	9	36.85595479	P63104-184	S	Phosphorylation	PSP	NULL	YES	P63104-184	p	YWHAZ	14-3-3 zeta	FYYEILNsPEKACSL	14-3-3	protein stabilization; molecular association, regulation; protein conformation; phosphorylation		HSP20(INDUCES); JNK1(NOT_REPORTED); BAX(DISRUPTS)	15071501; 16009721; 18559254; 21081103
27	IPR000308-222	IPR000308	37	222	Phosphorylation;Phosphorylation;Phosphorylation;Phosphorylation;Phosphorylation;Phosphorylation;Phosphorylation;Phosphorylation	8	0.216216216	90	0.40540541	0.28972973	1	0	6	12	14	0	0	30.37903231	P63104-207	S	Phosphorylation	PSP	NULL											
28	IPR000308-141	IPR000308	37	141	Phosphorylation;Phosphorylation;Phosphorylation;Phosphorylation;Phosphorylation;Phosphorylation;Phosphorylation;Phosphorylation	8	0.216216216	140	0.45945946	0.04837838	3	0	18	12	26	0	0	26.84265206	P63104-126	Y	Phosphorylation	PSP	NULL											
29	IPR000308-153	IPR000308	37	153	Ubiquitination;Acetylation;Ubiquitination;Ubiquitination;Ubiquitination;Acetylation;Ubiquitination;Ubiquitination;Ubiquitination;Acetylation;Ubiquitination	8	0.216216216	198	0.40540541	0.3072973	3	0	22	12	30	0	0	26.44957102	P63104-138	K	Acetylation	PSP	NULL											
29	IPR000308-153	IPR000308	37	153	Ubiquitination;Acetylation;Ubiquitination;Ubiquitination;Ubiquitination;Acetylation;Ubiquitination;Ubiquitination;Ubiquitination;Acetylation;Ubiquitination	8	0.216216216	198	0.40540541	0.3072973	3	0	22	12	30	0	0	26.44957102	P63104-138	K	Ubiquitination	PSP	NULL											
30	IPR000308-88	IPR000308	37	88	Acetylation;Acetylation;Ubiquitination;Acetylation;Ubiquitination;Acetylation;Ubiquitination;Acetylation	8	0.216216216	11	0.72972973	0.42135135	2	0	12	12	20	0	0	20.53258527	P63104-75	K	Acetylation	PSP	NULL											
31	IPR000308-229	IPR000308	37	229	Phosphorylation;Phosphorylation;Phosphorylation;Phosphorylation;Phosphorylation;Phosphorylation;Phosphorylation;Phosphorylation	8	0.216216216	8	1	0.28756757	2	0	9	12	17	0	0	19.40087718	P63104-214	S	Phosphorylation	PSP	NULL											
32	IPR000308-116	IPR000308	37	116	Ubiquitination;Acetylation;Ubiquitination;Ubiquitination;Acetylation;Ubiquitination;Ubiquitination	5	0.135135135	43	0.63888889	0.05777778	1	0	2	12	7	0	0	17.82726797	P63104-103	K	Ubiquitination	PSP	NULL											
33	IPR000308-140	IPR000308	37	140	Phosphorylation;Phosphorylation;Phosphorylation;Phosphorylation;Phosphorylation;Phosphorylation	6	0.162162162	33	1	0.0472973	2	0	9	12	15	0	0	17.78012196	P63104-125	Y	Phosphorylation	PSP	NULL											
34	IPR000308-27	IPR000308	37	27	Phosphorylation;Phosphorylation;Phosphorylation;Phosphorylation;Phosphorylation;Phosphorylation;Phosphorylation	7	0.189189189	16	1	0.35378378	0	0	0	12	7	0	0	17.49299388	P63104-19	Y														
35	IPR000308-35	IPR000308	37	35	Ubiquitination;Ubiquitination;Ubiquitination;Acetylation;Acetylation;Ubiquitination;Acetylation;Ubiquitination	6	0.162162162	28	0.89189189	0.32675676	2	0	10	12	16	0	0	17.33510793	P63104-27	K														

36	IPR000308-173	IPR000308	37	173	Ubiquitination;Ubiquitination;Ubiquitination;Ubiquitination;Acetylation;Ubiquitination;Ubiquitination	6	0.162162162	169	0.40540541	0.34702703	2	0	19	12	25	0	0	16.88893527	P63104-158	K	Ubiquitination	PSP	NULL									
37	IPR000308-220	IPR000308	37	220	Phosphorylation;Phosphorylation;Phosphorylation;Phosphorylation;Phosphorylation;Phosphorylation	6	0.162162162	15	1	0.22756757	1	0	8	12	14	0	0	16.76758109	P63104-205	T	Phosphorylation	PSP	NULL									
38	IPR000308-244	IPR000308	37	244	Phosphorylation;Phosphorylation;Phosphorylation;Phosphorylation;Phosphorylation;Phosphorylation;Phosphorylation	7	0.189189189	8	1	0.43567568	1	0	11	12	18	0	0	15.31455757	P63104-229	T	Phosphorylation	PSP	NULL									
39	IPR000308-230	IPR000308	37	230	Phosphorylation;Phosphorylation;Phosphorylation;Phosphorylation;Phosphorylation;Phosphorylation;Phosphorylation	7	0.189189189	7	1	0.27783784	1	0	8	12	15	0	0	14.64343404	P63104-215	T	Phosphorylation	PSP	NULL									
40	IPR000308-247	IPR000308	37	247	Phosphorylation;Phosphorylation;Phosphorylation;Phosphorylation;Phosphorylation	5	0.135135135	58	0.18918919	0.54324324	2	0	13	12	18	1	5	14.61626821	P63104-232	T	Phosphorylation	PSP	NULL	YES	P63104-232	p	YWHAZ	14-3-3 zeta	LTLWTSDtQGDEAEA	molecular association, regulation; protein conformation; activity, inhibited	14-3-3 zeta(DISRUPTS); RAF1(DISRUPTS); Tau iso3(DISRUPTS); HSP20(DISRUPTS)	14613942; 15119949; 18559254; 21081103; 9360956
41	IPR000308-107	IPR000308	37	107	S-Nitrosylation;S-Nitrosylation;S-Nitrosylation;S-Nitrosylation;S-Nitrosylation;S-Nitrosylation;S-Nitrosylation	7	0.189189189	7	0.94444444	0.12111111	3	0	6	12	13	0	0	14.39198424	P63104-94	C	S-Nitrosylation	dbSNOv2	19483679									
42	IPR000308-164	IPR000308	37	164	Phosphorylation;Phosphorylation;Phosphorylation;Phosphorylation;Phosphorylation	5	0.135135135	16	1	0.30702703	2	0	8	12	13	0	0	14.24616551	P63104-149	Y	Phosphorylation	PSP	NULL									
43	IPR000308-101	IPR000308	37	101	Acetylation;Phosphorylation;Phosphorylation;Phosphorylation;Phosphorylation;Phosphorylation	6	0.162162162	9	0.69444444	0.17166667	0	0	0	12	6	0	0	14.09316887	P63104-88	T	Phosphorylation	PSP	NULL									
44	IPR000308-98	IPR000308	37	98	Acetylation;Acetylation;Methylation;Ubiquitination;Ubiquitination;Acetylation;Ubiquitination;Ubiquitination	5	0.135135135	10	0.86111111	0.19666667	1	0	1	12	6	0	0	13.70549386	P63104-85	K	Acetylation	PSP	NULL									
44	IPR000308-98	IPR000308	37	98	Acetylation;Acetylation;Methylation;Ubiquitination;Ubiquitination;Acetylation;Ubiquitination;Ubiquitination	5	0.135135135	10	0.86111111	0.19666667	1	0	1	12	6	0	0	13.70549386	P63104-85	K	Methylation	PSP	NULL									
44	IPR000308-98	IPR000308	37	98	Acetylation;Acetylation;Methylation;Ubiquitination;Ubiquitination;Acetylation;Ubiquitination;Ubiquitination	5	0.135135135	10	0.86111111	0.19666667	1	0	1	12	6	0	0	13.70549386	P63104-85	K	Ubiquitination	PSP	NULL									
45	IPR000308-123	IPR000308	37	123	Phosphorylation;Phosphorylation;Phosphorylation;Phosphorylation;Phosphorylation	5	0.135135135	15	0.61111111	0.05083333	3	0	4	12	9	0	0	13.42277129	P63104-110	S	Phosphorylation	PSP	NULL									
46	IPR000308-8	IPR000308	37	8	Acetylation;Phosphorylation;Acetylation;Phosphorylation;Phosphorylation;Acetylation;Acetylation	6	0.162162162	6	1	0.87	2	0	5	12	11	0	0	11.21550438														
47	IPR000308-152	IPR000308	37	152	Acetylation;Ubiquitination;Acetylation;Ubiquitination;Ubiquitination;Acetylation;Ubiquitination	4	0.108108108	32	0.16216216	0.31756757	2	0	25	12	29	0	0	10.48368032	P63104-137	D												
48	IPR000308-36	IPR000308	37	36	Phosphorylation;Phosphorylation;Ubiquitination;Ubiquitination;Phosphorylation	5	0.135135135	6	0.56756757	0.31432432	2	0	10	12	15	0	0	10.11904373	P63104-28	S	Phosphorylation	PSP	NULL									
49	IPR000308-120	IPR000308	37	120	Ubiquitination;Ubiquitination;Ubiquitination;Ubiquitination;Ubiquitination	5	0.135135135	6	0.19444444	0.08694444	1	0	2	12	7	0	0	10.0750276	P63104-107	P												
50	IPR000308-33	IPR000308	37	33	S-Nitrosylation;S-Nitrosylation;S-Nitrosylation;S-Nitrosylation;S-Nitrosylation	5	0.135135135	5	0.16216216	0.36648649	2	0	9	12	14	0	0	8.736480866	P63104-25	C	S-Nitrosylation	dbSNOv2	2212679									
51	IPR000308-162	IPR000308	37	162	Ubiquitination;Phosphorylation;Acetylation;Ubiquitination	3	0.081081081	27	0.24324324	0.32432432	2	0	8	12	11	0	0	8.644292056	P63104-147	Q												
52	IPR000308-67	IPR000308	37	67	Phosphorylation;Phosphorylation;Phosphorylation;Phosphorylation	4	0.108108108	4	0.54054054	0.16864865	1	1	11	12	15	0	0	7.168650879	P63104-57	S	Phosphorylation	PSP	NULL									
53	IPR000308-80	IPR000308	37	80	Phosphorylation;Phosphorylation	2	0.054054054	13	0.08108108	0.4027027	3	1	31	12	33	0	0	6.716522459	P63104-70	E												
54	IPR000308-112	IPR000308	37	112	Phosphorylation;Phosphorylation;Phosphorylation	3	0.081081081	6	0.38888889	0.05805556	0	0	0	12	3	0	0	6.521809983	P63104-99	S	Phosphorylation	PSP	NULL									
55	IPR000308-93	IPR000308	37	93	Ubiquitination;Ubiquitination	2	0.054054054	12	0.56756757	0.38324324	2	0	4	12	6	0	0	6.042997756	P63104-80	R												
56	IPR000308-12	IPR000308	37	12	Phosphorylation;Phosphorylation	2	0.054054054	8	0.24324324	0.63918919	2	0	14	12	16	0	0	4.721819781	P63104-4	N												
57	IPR000308-85	IPR000308	37	85	Phosphorylation;Phosphorylation	2	0.054054054	8	0.21621622	0.4327027	1	0	10	12	12	1	1	4.721782642	P63104-72	A												
58	IPR000308-189	IPR000308	37	189	Phosphorylation	1	0.027027027	9	0.08108108	0.01837838	1	0	1	12	2	0	0	4.351763374	P63104-174	F												
59	IPR000308-134	IPR000308	37	134	Phosphorylation	1	0.027027027	6	0.27777778	0.09638889	2	1	34	12	35	0	0	4.292677236	P63104-119	L												
60	IPR000308-82	IPR000308	37	82	Ubiquitination;Ubiquitination;Acetylation	3	0.081081081	5	0.5	0.35681818	1	0	2	9	5	0	0	4.27921991														
61	IPR000308-165	IPR000308	37	165	Acetylation;Methylation;Ubiquitination;Phosphorylation	3	0.081081081	5	0.51351351	0.32324324	1	0	5	12	8	0	0	4.269627013	P63104-150	Q												
62	IPR000308-204	IPR000308	37	204	S-Nitrosylation;S-Nitrosylation;S-Nitrosylation	3	0.081081081	3	0.94444444	0.14222222	0	0	0	12	3	0	0	4.18024712	P63104-189	C	S-Nitrosylation	dbSNOv2	19483679									
63	IPR000308-283	IPR000308	37	283	Phosphorylation;Phosphorylation	2	0.054054054	8	0.1	0.973	1	0	1	12	3	0	0	4.07431687	P63104-245	N												
64	IPR000308-9	IPR000308	37	9	Acetylation;Acetylation;Acetylation;Acetylation	4	0.108108108	4	1	0.83756757	3	0	21	12	25	0	0	3.979461556	P63104-1	M	Acetylation	Uniport_201509	19608861									
65	IPR000308-193	IPR000308	37	193	Phosphorylation;Phosphorylation;Phosphorylation	3	0.081081081	3	0.91891892	0.03432432	1	1	1	12	4	0	0	3.513924354	P63104-178	Y	Phosphorylation	PSP	NULL									
66	IPR000308-178	IPR000308	37	178	Phosphorylation;Phosphorylation	2	0.054054054	5	1	0.19324324	0	0	0	12	2	0	0	3.506039414	P63104-163	T												
67	IPR000308-79	IPR000308	37	79	Phosphorylation;Phosphorylation	2	0.054054054	3	0.59459459	0.32837838	2	0	28	12	30	1	1	2.638549157	P63104-69	T												
68	IPR000308-133	IPR000308	37	133	Phosphorylation;Phosphorylation	2	0.054054054	2	0.94444444	0.13277778	2	1	33	12	35	0	0	2.51149793	P63104-118	Y												
69	IPR000308-108	IPR000308	37	108	S-Nitrosylation;S-Nitrosylation;Phosphorylation	3	0.081081081	3	0.22222222	0.09166667	2	0	8	12	11	0	0	2.377650151	P63104-95	N												
70	IPR000308-3	IPR000308	37	3	Acetylation	1	0.027027027	1	1	0.915	1	0	1	1	2	0	0	2.26058741														
71	IPR000308-2	IPR000308	37	2	Acetylation	1	0.027027027	1	1	0.925	1	0	1	1	2	0	0	2.248744589														
72	IPR000308-160	IPR000308	37	160	Phosphorylation;Phosphorylation;Phosphorylation	3	0.081081081	3	0.94594595	0.42	2	0	5	12	8	0	0	2.244197849	P63104-145	S	Phosphorylation	PSP	NULL									
73	IPR000308-256	IPR000308	37	256	Ubiquitination;Ubiquitination	2	0.054054054	4	0.58333333	0.70916667	0	0	0	7	2	0	0	2.202907667														
74	IPR000308-227	IPR000308	37	227	Methylation;Methylation	2	0.054054054	2	1	0.21540541	3	0	26	12	28	0	0	2.158940107	P63104-212	K	Methylation	PSP	NULL									
75	IPR000308-209	IPR000308	37	209	Phosphorylation;Phosphorylation	2	0.054054054	3	0.47222222	0.14222222	1	0	10	12	12	0	0	1.8569283	P63104-194	T	Phosphorylation	PSP	NULL									
76	IPR000308-156	IPR000308	37	156	Phosphorylation;Phosphorylation;Phosphorylation	3	0.081081081	2	0.27027027	0.37891892	2	0	15	12	18	1	1	1.780563559	P63104-141	I												
77	IPR000308-145	IPR000308	37	145	Phosphorylation;Phosphorylation	2	0.054054054	3	0.18918919	0.22675676	1	0	11	12	13	1	1	1.697803042	P63104-130	A												
78	IPR000308-95	IPR000308	37	95	Phosphorylation;Nitration;Phosphorylation	3	0.081081081	2	1	0.31891892	3	0	4	12	7	0	0	1.55235857	P63104-82	Y	Phosphorylation	PSP	NULL									
79	IPR000308-16	IPR000308	37	16	Phosphorylation	1	0.027027027	3	0.40540541	0.49486486	1	0	14	12	15	0	0	1.499626888	P63104-8	Q												
80	IPR000308-90	IPR000308	37	90	Ubiquitination;Ubiquitination	2	0.054054054	3	0.16216216	0.37108108	1	0	8	12	10	0	0	1.490418168	P63104-77	Q												
81	IPR000308-241	IPR000308	37	241	Phosphorylation;Phosphorylation	2	0.054054054	2	1	0.25162162	0	0	0	12	2	0	0	1.448409944	P63104-226	T												
82	IPR000308-142	IPR000308	37	142	Methylation	1	0.027027027	1	1	0.06918919	3	0	25	12	26	0	0	1.260798708	P63104-127	R												
83	IPR000308-296	IPR000308	37	296	Phosphorylation	1	0.027027027	1	0.5	0.985	0	0	0	2	1	0	0	1.136152823														
84	IPR000308-105	IPR000308	37	105	Phosphorylation;Phosphorylation	2	0.054054054	2	0.13888889	0.07555556	1	0	7	12	9	0	0	1.132458341	P63104-92	D												
85	IPR000308-38	IPR000308	37	38	Phosphorylation;Phosphorylation	2	0.054054054	2	0.54054054	0.36918919	3	0	7	12	9	0	0	1.047226356	P63104-30	T												
86	IPR000308-122	IPR000308	37	122	S-Nitrosylation;S-Nitrosylation	2	0.054054054	2	0.13888889	0.08166667	3	0	11	12	13	0	0	1.040024513	P63104-109	A												
87	IPR000308-155	IPR000308	37	155	Phosphorylation	1	0.027027027	1	0.35135135	0.34675676	3	1	28	12	29	0	0	1.031509615	P63104-140	G												
88	IPR000308-171	IPR000308	37	171	Phosphorylation	1	0.027027027	1	0.54054054	0.40405405	2	0	25	12	26	0	0	1.001336876	P63104-156	S												
89	IPR000308-194	IPR000308	37	194	Phosphorylation	1	0.027027027	2	1	0.04648649	1	0	3	12	4	1	1	0.885332368	P63104-179	Y												

90	IPR000308-117	IPR000308	37	117	Nitration;Phosphorylation	2	0.054054054	1	0.30555556	0.09416667	1	0	7	12	9	0	0	0.839455489	P63104-104	F
91	IPR000308-248	IPR000308	37	248	Phosphorylation;Phosphorylation	2	0.054054054	2	0.10810811	0.57216216	1	1	5	12	7	0	0	0.837512982	P63104-233	Q
92	IPR000308-109	IPR000308	37	109	Phosphorylation	1	0.027027027	2	0.11111111	0.06083333	2	0	10	12	11	0	0	0.732576817	P63104-96	D
93	IPR000308-7	IPR000308	37	7	Acetylation	1	0.027027027	1	1	0.88333333	2	0	11	11	12	0	0	0.730493098		
94	IPR000308-125	IPR000308	37	125	Phosphorylation	1	0.027027027	2	0.08333333	0.08861111	3	0	9	12	10	0	0	0.69157603	P63104-112	A
95	IPR000308-32	IPR000308	37	32	Phosphorylation	1	0.027027027	2	0.10810811	0.38810811	1	0	5	12	6	0	0	0.600443693	P63104-24	A
96	IPR000308-13	IPR000308	37	13	Phosphorylation	1	0.027027027	1	0.08108108	0.61567568	2	0	15	12	16	0	0	0.524528817	P63104-5	E
97	IPR000308-190	IPR000308	37	190	Phosphorylation	1	0.027027027	1	1	0.01648649	1	0	1	12	2	0	0	0.518818056	P63104-175	S
98	IPR000308-51	IPR000308	37	51	Methylation	1	0.027027027	1	1	0.27837838	0	0	0	12	1	0	0	0.492826868	P63104-41	R
99	IPR000308-97	IPR000308	37	97	Phosphorylation	1	0.027027027	1	0.11111111	0.24416667	2	0	11	12	12	0	0	0.458923839	P63104-84	E
100	IPR000308-124	IPR000308	37	124	Phosphorylation	1	0.027027027	1	0.27777778	0.07333333	3	0	8	12	9	0	0	0.458596532	P63104-111	Q
101	IPR000308-48	IPR000308	37	48	S-Nitrosylation	1	0.027027027	1	0.05405405	0.26648649	1	0	10	12	11	0	0	0.426302426	P63104-38	N
102	IPR000308-159	IPR000308	37	159	Phosphorylation	1	0.027027027	1	0.24324324	0.39243243	1	0	3	12	4	0	0	0.338302921	P63104-144	Q
103	IPR000308-94	IPR000308	37	94	Phosphorylation	1	0.027027027	1	0.08108108	0.32297297	2	0	5	12	6	0	0	0.33802711	P63104-81	E
104	IPR000308-39	IPR000308	37	39	Phosphorylation	1	0.027027027	1	0.10810811	0.35297297	2	0	3	12	4	0	0	0.311245454	P63104-31	E
105	IPR000308-40	IPR000308	37	40	Acetylation	1	0.027027027	1	0.05405405	0.33702703	2	0	3	12	4	0	0	0.301444697	P63104-32	Q
106	IPR000308-252	IPR000308	37	252	Phosphorylation	1	0.027027027	1	0.07692308	0.57615385	1	0	1	7	2	0	0	0.290038696		
107	IPR000308-251	IPR000308	37	251	Phosphorylation	1	0.027027027	1	0.07142857	0.565	1	0	1	7	2	0	0	0.289072834		
108	IPR000308-282	IPR000308	37	282	Phosphorylation	1	0.027027027	1	0.05555556	0.96611111	1	0	2	12	3	0	0	0.287635714	P63104-244	E
109	IPR000308-279	IPR000308	37	279	Phosphorylation	1	0.027027027	1	0.05405405	0.91405405	0	0	0	12	1	0	0	0.263642489	P63104-241	E
