# Supplementary material for: The dynamic and stress-adaptive signaling hub of 14-3-3: emerging mechanisms of regulation and context-dependent protein–protein interactions
Source: Oncogene. 2018 Jun 18;37(42):5587–604. doi: 10.1038/s41388-018-0348-3 (PMC6193947; doi:10.1038/s41388-018-0348-3)
Supplement: Supplementary file 3 — Supplemental Table S1. Tab3 [file 41388_2018_348_MOESM3_ESM.pdf]

MAP	Family	UID-NP	UID	NP	AP	UniProt name	OID	Length	Resi	Native PTM	Database	Reference	Resi_PSPfxn	PTM_PSPfxn	GENE_PSPfxn	PROTEIN_PSPfxn	PROT_TYPE_PSPfxn	GENE_ID_PSPfxn	HU_CHR_LOC_PSPfxn	ORGANISM_PSPfxn	SITE_GRP_ID_PSPfxn	SITE_+/-7_AA_PSPfxn	DOMAIN_PSPfxn	ON_FUNCTION_PSPfxn	ON_PROCESS_PSPfxn	ON_PROT_INTERACT_PSPfxn	ON_OTHER_INTERACT_PSPfxn	PMIDs_PSPfxn	NOTES_PSPfxn
IPR000308-2	IPR000308	P46077-2	P46077	2	2	14334_ARATH	Arabidopsis thaliana	267	A	Acetylation	Uniport_201509	22223895																	
IPR000308-3	IPR000308	P42643-2	P42643	2	3	14331_ARATH	Arabidopsis thaliana	267	A	Acetylation	Uniport_201509	22223895																	
IPR000308-7	IPR000308	P31946-1	P31946	1	7	1433B_HUMAN	Homo sapiens	246	M	Acetylation	Uniport_201509	22814378																	
IPR000308-8	IPR000308	P29311-2	P29311	2	8	BMH1_YEAST	Saccharomyces cerevisiae	267	S	Acetylation	Uniport_201509	9298649																	
IPR000308-8	IPR000308	P31946-2	P31946	2	8	1433B_HUMAN	Homo sapiens	246	T	Acetylation	Uniport_201509	22814378																	
IPR000308-8	IPR000308	P31946-2	P31946	2	8	1433B_HUMAN	Homo sapiens	246	T	Phosphorylation	PSP	NULL																	
IPR000308-8	IPR000308	P34730-2	P34730	2	8	BMH2_YEAST	Saccharomyces cerevisiae	273	S	Acetylation	Uniport_201509	9298649																	
IPR000308-8	IPR000308	P41932-2	P41932	2	8	14331_CAEEL	Caenorhabditis elegans	248	S	Phosphorylation	PhosphoELM.10011	19530675																	
IPR000308-8	IPR000308	P62258-1	P62258	1	8	1433E_HUMAN	Homo sapiens	255	M	Acetylation	Uniport_201509	19413330																	
IPR000308-8	IPR000308	Q9CQV8-2	Q9CQV8	2	8	1433B_MOUSE	Mus musculus	246	T	Phosphorylation	PSP	NULL																	
IPR000308-9	IPR000308	P27348-1	P27348	1	9	1433T_HUMAN	Homo sapiens	245	M	Acetylation	Uniport_201509	19608861																	
IPR000308-9	IPR000308	P29361-1	P29361	1	9	1433Z_SHEEP	Ovis aries	245	M	Acetylation	Uniport_201509	1317796																	
IPR000308-9	IPR000308	P61981-2	P61981	2	9	1433G_HUMAN	Homo sapiens	247	V	Acetylation	Uniport_201509	14534293																	
IPR000308-9	IPR000308	P63104-1	P63104	1	9	1433Z_HUMAN	Homo sapiens	245	M	Acetylation	Uniport_201509	19608861																	
IPR000308-11	IPR000308	P27348-3	P27348	3	11	1433T_HUMAN	Homo sapiens	245	K	Acetylation	PSP	NULL																	
IPR000308-11	IPR000308	P27348-3	P27348	3	11	1433T_HUMAN	Homo sapiens	245	K	Ubiquitination	PSP	NULL																	
IPR000308-11	IPR000308	P31946-5	P31946	5	11	1433B_HUMAN	Homo sapiens	246	K	Acetylation	PSP	NULL																	
IPR000308-11	IPR000308	P31946-5	P31946	5	11	1433B_HUMAN	Homo sapiens	246	K	Ubiquitination	PSP	NULL																	
IPR000308-11	IPR000308	P35213-5	P35213	5	11	1433B_RAT	Rattus norvegicus	246	K	Acetylation	PSP	NULL																	
IPR000308-11	IPR000308	P62258-4	P62258	4	11	1433E_HUMAN	Homo sapiens	255	R	Methylation	PSP	NULL																	
IPR000308-11	IPR000308	P63101-3	P63101	3	11	1433Z_MOUSE	Mus musculus	245	K	Acetylation	PSP	NULL																	
IPR000308-11	IPR000308	P63102-3	P63102	3	11	1433Z_RAT	Rattus norvegicus	245	K	Acetylation	PSP	NULL																	
IPR000308-11	IPR000308	P63104-3	P63104	3	11	1433Z_HUMAN	Homo sapiens	245	K	Acetylation	PSP	NULL																	
IPR000308-11	IPR000308	P63104-3	P63104	3	11	1433Z_HUMAN	Homo sapiens	245	K	Methylation	PSP	NULL																	
IPR000308-11	IPR000308	P63104-3	P63104	3	11	1433Z_HUMAN	Homo sapiens	245	K	Ubiquitination	PSP	NULL																	
IPR000308-11	IPR000308	P68254-3	P68254	3	11	1433T_MOUSE	Mus musculus	245	K	Acetylation	PSP	NULL																	
IPR000308-11	IPR000308	Q9CQV8-5	Q9CQV8	5	11	1433B_MOUSE	Mus musculus	246	K	Acetylation	PSP	NULL																	
IPR000308-12	IPR000308	P31946-6	P31946	6	12	1433B_HUMAN	Homo sapiens	246	S	Phosphorylation	PSP	NULL																	
IPR000308-12	IPR000308	Q9CQV8-6	Q9CQV8	6	12	1433B_MOUSE	Mus musculus	246	S	Phosphorylation	PSP	NULL																	
IPR000308-13	IPR000308	P31947-5	P31947	5	13	1433S_HUMAN	Homo sapiens	248	S	Phosphorylation	PSP	NULL																	
IPR000308-16	IPR000308	P62259-9	P62259	9	16	1433E_MOUSE	Mus musculus	255	Y	Phosphorylation	PSP	NULL																	
IPR000308-17	IPR000308	P27348-9	P27348	9	17	1433T_HUMAN	Homo sapiens	245	K	Acetylation	PSP	NULL																	
IPR000308-17	IPR000308	P27348-9	P27348	9	17	1433T_HUMAN	Homo sapiens	245	K	Ubiquitination	PSP	NULL																	
IPR000308-17	IPR000308	P31946-11	P31946	11	17	1433B_HUMAN	Homo sapiens	246	K	Ubiquitination	PSP	NULL																	
IPR000308-17	IPR000308	P35213-11	P35213	11	17	1433B_RAT	Rattus norvegicus	246	K	Acetylation	PSP	NULL																	
IPR000308-17	IPR000308	P35213-11	P35213	11	17	1433B_RAT	Rattus norvegicus	246	K	Ubiquitination	PSP	NULL																	
IPR000308-17	IPR000308	P61981-10	P61981	10	17	1433G_HUMAN	Homo sapiens	247	K	Ubiquitination	PSP	NULL																	
IPR000308-17	IPR000308	P61982-10	P61982	10	17	1433G_MOUSE	Mus musculus	247	K	Ubiquitination	PSP	NULL																	
IPR000308-17	IPR000308	P63101-9	P63101	9	17	1433Z_MOUSE	Mus musculus	245	K	Ubiquitination	PSP	NULL																	
IPR000308-17	IPR000308	P63102-9	P63102	9	17	1433Z_RAT	Rattus norvegicus	245	K	Acetylation	PSP	NULL																	
IPR000308-17	IPR000308	P63102-9	P63102	9	17	1433Z_RAT	Rattus norvegicus	245	K	Ubiquitination	PSP	NULL																	
IPR000308-17	IPR000308	P63104-9	P63104	9	17	1433Z_HUMAN	Homo sapiens	245	K	Acetylation	PSP	NULL																	
IPR000308-17	IPR000308	P63104-9	P63104	9	17	1433Z_HUMAN	Homo sapiens	245	K	Ubiquitination	PSP	NULL																	
IPR000308-17	IPR000308	P68254-9	P68254	9	17	1433T_MOUSE	Mus musculus	245	K	Ubiquitination	PSP	NULL																	
IPR000308-17	IPR000308	Q9CQV8-11	Q9CQV8	11	17	1433B_MOUSE	Mus musculus	246	K	Ubiquitination	PSP	NULL																	
IPR000308-19	IPR000308	O70456-11	O70456	11	19	1433S_MOUSE	Mus musculus	248	K	Ubiquitination	PSP	NULL																	
IPR000308-19	IPR000308	P27348-11	P27348	11	19	1433T_HUMAN	Homo sapiens	245	K	Acetylation	PSP	NULL																	
IPR000308-19	IPR000308	P27348-11	P27348	11	19	1433T_HUMAN	Homo sapiens	245	K	Ubiquitination	PSP	NULL																	
IPR000308-19	IPR000308	P31946-13	P31946	13	19	1433B_HUMAN	Homo sapiens	246	K	Acetylation	PSP	NULL																	
IPR000308-19	IPR000308	P31946-13	P31946	13	19	1433B_HUMAN	Homo sapiens	246	K	Ubiquitination	PSP	NULL																	
IPR000308-19	IPR000308	P31947-11	P31947	11	19	1433S_HUMAN	Homo sapiens	248	K	Acetylation	PSP	NULL																	
IPR000308-19	IPR000308	P31947-11	P31947	11	19	1433S_HUMAN	Homo sapiens	248	K	Ubiquitination	PSP	NULL																	
IPR000308-19	IPR000308	P35213-13	P35213	13	19	1433B_RAT	Rattus norvegicus	246	K	Acetylation	PSP	NULL																	
IPR000308-19	IPR000308	P35213-13	P35213	13	19	1433B_RAT	Rattus norvegicus	246	K	Ubiquitination	PSP	NULL																	
IPR000308-19	IPR000308	P62258-12	P62258	12	19	1433E_HUMAN	Homo sapiens	255	K	Acetylation	PSP	NULL																	
IPR000308-19	IPR000308	P62258-12	P62258	12	19	1433E_HUMAN	Homo sapiens	255	K	Ubiquitination	PSP	NULL																	
IPR000308-19	IPR000308	P62259-12	P62259	12	19	1433E_MOUSE	Mus musculus	255	K	Acetylation	PSP	NULL																	
IPR000308-19	IPR000308	P62259-12	P62259	12	19	1433E_MOUSE	Mus musculus	255	K	Ubiquitination	PSP	NULL																	
IPR000308-19	IPR000308	P62260-12	P62260	12	19	1433E_RAT	Rattus norvegicus	255	K	Acetylation	PSP	NULL																	
IPR000308-19	IPR000308	P62260-12	P62260	12	19	1433E_RAT	Rattus norvegicus	255	K	Ubiquitination	PSP	NULL																	
IPR000308-19	IPR000308	P63101-11	P63101	11	19	1433Z_MOUSE	Mus musculus	245	K	Ubiquitination	PSP	NULL																	
IPR000308-19	IPR000308	P63102-11	P63102	11	19	1433Z_RAT	Rattus norvegicus	245	K	Acetylation	PSP	NULL																	
IPR000308-19	IPR000308	P63102-11	P63102	11	19	1433Z_RAT	Rattus norvegicus	245	K	Ubiquitination	PSP	NULL																	
IPR000308-19	IPR000308	P63104-11	P63104	11	19	1433Z_HUMAN	Homo sapiens	245	K	Acetylation	PSP	NULL																	
IPR000308-19	IPR000308	P63104-11	P63104	11	19	1433Z_HUMAN	Homo sapiens	245	K	Ubiquitination	PSP	NULL																	
IPR000308-19	IPR000308	P68254-11	P68254	11	19	1433T_MOUSE	Mus musculus	245	K	Ubiquitination	PSP	NULL																	
IPR000308-19	IPR000308	Q9CQV8-13	Q9CQV8	13	19	1433B_MOUSE	Mus musculus	246	K	Ubiquitination	PSP	NULL																	
IPR000308-27	IPR000308	O70456-19	O70456	19	27	1433S_MOUSE	Mus musculus	248	Y	Phosphorylation	PSP	NULL																	
IPR000308-27	IPR000308	P27348-19	P27348	19	27	1433T_HUMAN	Homo sapiens	245	Y	Phosphorylation	PSP	NULL																	
IPR000308-27	IPR000308	P31946-21	P31946	21	27	1433B_HUMAN	Homo sapiens	246	Y	Phosphorylation	PSP	NULL																	
IPR000308-27	IPR000308	P31947-19	P31947	19	27	1433S_HUMAN	Homo sapiens	248	Y	Phosphorylation	PSP	NULL																	
IPR000308-27	IPR000308	P35213-21	P35213	21	27	1433B_RAT	Rattus norvegicus	246	Y	Phosphorylation	PSP	NULL																	
IPR000308-27	IPR000308	P61981-20	P61981	20	27	1433G_HUMAN	Homo sapiens	247	Y	Phosphorylation	PSP	NULL																	
IPR000308-27	IPR000308	Q04917-20	Q04917	20	27	1433F_HUMAN	Homo sapiens	246	Y	Phosphorylation	PSP	NULL																	
IPR000308-32	IPR000308	Q04917-25	Q04917	25	32	1433F_HUMAN	Homo sapiens	246	S	Phosphorylation	PSP	NULL																	
IPR000308-33	IPR000308	P27348-25	P27348	25	33	1433T_HUMAN	Homo sapiens	245	C	S-Nitrosylation	dbSNOv2	16648260																	
IPR000308-33	IPR000308	P63101-25	P63101	25	33	1433Z_MOUSE	Mus musculus	245	C	S-Nitrosylation	dbSNOv2	20925432																	
IPR000308-33	IPR000308	P63102-25	P63102	25	33	1433Z_RAT	Rattus norvegicus	245	C	S-Nitrosylation	dbSNOv2	17629318																	
IPR000308-33	IPR000308	P63104-25	P63104	25	33	1433Z_HUMAN	Homo sapiens	245	C	S-Nitrosylation	dbSNOv2	2212679																	
IPR000308-33	IPR000308	P68254-25	P68254	25	33	1433T_MOUSE	Mus musculus	245	C	S-Nitrosylation	dbSNOv2	20925432																	
IPR000308-35	IPR000308	P27348-27	P27348	27	35	1433T_HUMAN	Homo sapiens	245	K	Acetylation	PSP	NULL																	
IPR000308-35	IPR000308	P27348-27	P27348	27	35	1433T_HUMAN	Homo sapiens	245	K	Ubiquitination	PSP	NULL																	
IPR000308-35	IPR000308	P35213-29	P35213	29	35	1433B_RAT	Rattus norvegicus	246	K	Ubiquitination	PSP	NULL																	
IPR000308-35	IPR000308	P61982-28	P61982	28	35	1433G_MOUSE	Mus musculus	247	K	Ubiquitination	PSP	NULL																	
IPR000308-35	IPR000308	P62258-28	P62258	28	35	1433E_HUMAN	Homo sapiens	255	K	Acetylation	PSP	NULL																	
IPR000308-35	IPR000308	P62258-28	P62258	28	35	1433E_HUMAN	Homo sapiens	255	K	Ubiquitination	PSP	NULL																	
IPR000308-35	IPR000308	P62259-28	P62259	28	35	1433E_MOUSE	Mus musculus	255	K	Acetylation	PSP	NULL																	
IPR000308-35	IPR000308	Q9CQV8-29	Q9CQV8	29	35	1433B_MOUSE	Mus musculus	246	K	Ubiquitination	PSP	NULL																	

IPR000308-36	IPR000308	O70456-28	O70456	28	36	1433S_MOUSE	Mus musculus	248	S	Phosphorylation	PSP	NULL															
IPR000308-36	IPR000308	P62258-29	P62258	29	36	1433E_HUMAN	Homo sapiens	255	K	Ubiquitination	PSP	NULL															
IPR000308-36	IPR000308	P62259-29	P62259	29	36	1433E_MOUSE	Mus musculus	255	K	Ubiquitination	PSP	NULL															
IPR000308-36	IPR000308	P63101-28	P63101	28	36	1433Z_MOUSE	Mus musculus	245	S	Phosphorylation	PSP	NULL															
IPR000308-36	IPR000308	P63104-28	P63104	28	36	1433Z_HUMAN	Homo sapiens	245	S	Phosphorylation	PSP	NULL															
IPR000308-38	IPR000308	P31946-32	P31946	32	38	1433B_HUMAN	Homo sapiens	246	T	Phosphorylation	PSP	NULL															
IPR000308-38	IPR000308	P61981-31	P61981	31	38	1433G_HUMAN	Homo sapiens	247	T	Phosphorylation	PSP	NULL															
IPR000308-39	IPR000308	P42656-34	P42656	34	39	RAD24_SCHPO	Schizosaccharomyces pombe	270	S	Phosphorylation	Uniport_201509	18257517															
IPR000308-40	IPR000308	P31947-32	P31947	32	40	1433S_HUMAN	Homo sapiens	248	K	Acetylation	PSP	NULL															
IPR000308-47	IPR000308	P27348-37	P27348	37	47	1433T_HUMAN	Homo sapiens	245	S	Phosphorylation	PSP	NULL															
IPR000308-47	IPR000308	P31946-39	P31946	39	47	1433B_HUMAN	Homo sapiens	246	S	Phosphorylation	PSP	NULL															
IPR000308-47	IPR000308	P61981-38	P61981	38	47	1433G_HUMAN	Homo sapiens	247	S	Phosphorylation	PSP	NULL															
IPR000308-47	IPR000308	P61982-38	P61982	38	47	1433G_MOUSE	Mus musculus	247	S	Phosphorylation	PSP	NULL															
IPR000308-47	IPR000308	P62258-38	P62258	38	47	1433E_HUMAN	Homo sapiens	255	T	Phosphorylation	PSP	NULL															
IPR000308-47	IPR000308	P63101-37	P63101	37	47	1433Z_MOUSE	Mus musculus	245	S	Phosphorylation	PSP	NULL															
IPR000308-47	IPR000308	P63104-37	P63104	37	47	1433Z_HUMAN	Homo sapiens	245	S	Phosphorylation	PSP	NULL															
IPR000308-47	IPR000308	P68254-37	P68254	37	47	1433T_MOUSE	Mus musculus	245	S	Phosphorylation	PSP	NULL															
IPR000308-47	IPR000308	P68510-38	P68510	38	47	1433F_MOUSE	Mus musculus	246	S	Phosphorylation	PSP	NULL															
IPR000308-47	IPR000308	Q04917-38	Q04917	38	47	1433F_HUMAN	Homo sapiens	246	S	Phosphorylation	PSP	NULL															
IPR000308-48	IPR000308	P31947-38	P31947	38	48	1433S_HUMAN	Homo sapiens	248	C	S-Nitrosylation	dbSNOv2	22178444															
IPR000308-51	IPR000308	Q04917-42	Q04917	42	51	1433F_HUMAN	Homo sapiens	246	R	Methylation	PSP	NULL															
IPR000308-55	IPR000308	O70456-45	O70456	45	55	1433S_MOUSE	Mus musculus	248	S	Phosphorylation	PSP	NULL															
IPR000308-55	IPR000308	P27348-45	P27348	45	55	1433T_HUMAN	Homo sapiens	245	S	Phosphorylation	PSP	NULL															
IPR000308-55	IPR000308	P31946-47	P31946	47	55	1433B_HUMAN	Homo sapiens	246	S	Phosphorylation	PSP	NULL															
IPR000308-55	IPR000308	P31947-45	P31947	45	55	1433S_HUMAN	Homo sapiens	248	S	Phosphorylation	PSP	NULL															
IPR000308-55	IPR000308	P35213-47	P35213	47	55	1433B_RAT	Rattus norvegicus	246	S	Phosphorylation	PSP	NULL															
IPR000308-55	IPR000308	P61981-46	P61981	46	55	1433G_HUMAN	Homo sapiens	247	S	Phosphorylation	PSP	NULL															
IPR000308-55	IPR000308	P61982-46	P61982	46	55	1433G_MOUSE	Mus musculus	247	S	Phosphorylation	PSP	NULL															
IPR000308-55	IPR000308	P62258-46	P62258	46	55	1433E_HUMAN	Homo sapiens	255	S	Phosphorylation	PSP	NULL															
IPR000308-55	IPR000308	P62259-46	P62259	46	55	1433E_MOUSE	Mus musculus	255	S	Phosphorylation	PSP	NULL															
IPR000308-55	IPR000308	P62260-46	P62260	46	55	1433E_RAT	Rattus norvegicus	255	S	Phosphorylation	PSP	NULL															
IPR000308-55	IPR000308	P63101-45	P63101	45	55	1433Z_MOUSE	Mus musculus	245	S	Phosphorylation	PSP	NULL															
IPR000308-55	IPR000308	P63102-45	P63102	45	55	1433Z_RAT	Rattus norvegicus	245	S	Phosphorylation	PSP	NULL															
IPR000308-55	IPR000308	P63104-45	P63104	45	55	1433Z_HUMAN	Homo sapiens	245	S	Phosphorylation	PSP	NULL															
IPR000308-55	IPR000308	P68254-45	P68254	45	55	1433T_MOUSE	Mus musculus	245	S	Phosphorylation	PSP	NULL															
IPR000308-55	IPR000308	P68510-46	P68510	46	55	1433F_MOUSE	Mus musculus	246	S	Phosphorylation	PSP	NULL															
IPR000308-55	IPR000308	Q04917-46	Q04917	46	55	1433F_HUMAN	Homo sapiens	246	S	Phosphorylation	PSP	NULL															
IPR000308-55	IPR000308	Q20655-47	Q20655	47	55	14332_CAEEL	Caenorhabditis elegans	248	S	Phosphorylation	PhosphoELM.10011	19530675															
IPR000308-55	IPR000308	Q9CQV8-47	Q9CQV8	47	55	1433B_MOUSE	Mus musculus	246	S	Phosphorylation	PSP	NULL															
IPR000308-58	IPR000308	O70456-48	O70456	48	58	1433S_MOUSE	Mus musculus	248	Y	Phosphorylation	PSP	NULL															
IPR000308-58	IPR000308	P27348-48	P27348	48	58	1433T_HUMAN	Homo sapiens	245	Y	Phosphorylation	PSP	NULL															
IPR000308-58	IPR000308	P31946-50	P31946	50	58	1433B_HUMAN	Homo sapiens	246	Y	Phosphorylation	PSP	NULL															
IPR000308-58	IPR000308	P31947-48	P31947	48	58	1433S_HUMAN	Homo sapiens	248	Y	Phosphorylation	PSP	NULL															
IPR000308-58	IPR000308	P61981-49	P61981	49	58	1433G_HUMAN	Homo sapiens	247	Y	Phosphorylation	PSP	NULL															
IPR000308-58	IPR000308	P61982-49	P61982	49	58	1433G_MOUSE	Mus musculus	247	Y	Phosphorylation	PSP	NULL															
IPR000308-58	IPR000308	P62258-49	P62258	49	58	1433E_HUMAN	Homo sapiens	255	Y	Phosphorylation	PSP	NULL															
IPR000308-58	IPR000308	P62259-49	P62259	49	58	1433E_MOUSE	Mus musculus	255	Y	Phosphorylation	PSP	NULL															
IPR000308-58	IPR000308	P63101-48	P63101	48	58	1433Z_MOUSE	Mus musculus	245	Y	Phosphorylation	PSP	NULL															
IPR000308-58	IPR000308	P63104-48	P63104	48	58	1433Z_HUMAN	Homo sapiens	245	Y	Phosphorylation	PSP	NULL															
IPR000308-58	IPR000308	P68254-48	P68254	48	58	1433T_MOUSE	Mus musculus	245	Y	Phosphorylation	PSP	NULL															
IPR000308-58	IPR000308	P68510-49	P68510	49	58	1433F_MOUSE	Mus musculus	246	Y	Phosphorylation	PSP	NULL															
IPR000308-58	IPR000308	Q04917-49	Q04917	49	58	1433F_HUMAN	Homo sapiens	246	Y	Phosphorylation	PSP	NULL															
IPR000308-58	IPR000308	Q9CQV8-50	Q9CQV8	50	58	1433B_MOUSE	Mus musculus	246	Y	Phosphorylation	PSP	NULL															
IPR000308-59	IPR000308	P61982-50	P61982	50	59	1433G_MOUSE	Mus musculus	247	K	Acetylation	PSP	NULL	K	ac	Ywhag	14-3-3 gamma	Adaptor/scaffold	22628	5|5 G2	mouse	474722	NLLSVAYkNVVGARR	14-3-3	molecular association, regulation	14-3-3 gamma(INDUCES); STAR(INDUCES)	25086053
IPR000308-59	IPR000308	P61982-50	P61982	50	59	1433G_MOUSE	Mus musculus	247	K	Ubiquitination	PSP	NULL	K	ac	Ywhag	14-3-3 gamma	Adaptor/scaffold	22628	5|5 G2	mouse	474722	NLLSVAYkNVVGARR	14-3-3	molecular association, regulation	14-3-3 gamma(INDUCES); STAR(INDUCES)	25086053
IPR000308-59	IPR000308	P62258-50	P62258	50	59	1433E_HUMAN	Homo sapiens	255	K	Acetylation	PSP	NULL	K	ac	YWHAE	14-3-3 epsilon	Adaptor/scaffold	7531	17p13.3	human	11315836	NLLSVAYkNVIGARR	14-3-3	molecular association, regulation	SFRS6(DISRUPTS); PLEKHQ1(DISRUPTS); SFRS4(DISRUPTS); DOK3(DISRUPTS); TSC2(DISRUPTS); ARAF(DISRUPTS); LARP(DISRUPTS); SRm300(DISRUPTS); SF2(DISRUPTS); TSC22D1(DISRUPTS); RAF1(DISRUPTS); GAB2(DISRUPTS); VASP(DISRUPTS); HDAC4(DISRUPTS); TRA2B(DISRUPTS); GBF1(DISRUPTS)	19608861
IPR000308-59	IPR000308	P62258-50	P62258	50	59	1433E_HUMAN	Homo sapiens	255	K	Ubiquitination	PSP	NULL	K	ac	YWHAE	14-3-3 epsilon	Adaptor/scaffold	7531	17p13.3	human	11315836	NLLSVAYkNVIGARR	14-3-3	molecular association, regulation	SFRS6(DISRUPTS); PLEKHQ1(DISRUPTS); SFRS4(DISRUPTS); DOK3(DISRUPTS); TSC2(DISRUPTS); ARAF(DISRUPTS); LARP(DISRUPTS); SRm300(DISRUPTS); SF2(DISRUPTS); TSC22D1(DISRUPTS); RAF1(DISRUPTS); GAB2(DISRUPTS); VASP(DISRUPTS); HDAC4(DISRUPTS); TRA2B(DISRUPTS); GBF1(DISRUPTS)	19608861
IPR000308-59	IPR000308	P27348-49	P27348	49	59	1433T_HUMAN	Homo sapiens	245	K	Acetylation	PSP	NULL															
IPR000308-59	IPR000308	P27348-49	P27348	49	59	1433T_HUMAN	Homo sapiens	245	K	Ubiquitination	PSP	NULL															
IPR000308-59	IPR000308	P31946-51	P31946	51	59	1433B_HUMAN	Homo sapiens	246	K	Acetylation	PSP	NULL															

IPR000308-59	IPR000308	P31946-51	P31946	51	59	1433B_HUMAN	Homo sapiens	246	K	Methylation	PSP	NULL																
IPR000308-59	IPR000308	P31946-51	P31946	51	59	1433B_HUMAN	Homo sapiens	246	K	Ubiquitination	PSP	NULL																
IPR000308-59	IPR000308	P31947-49	P31947	49	59	1433S_HUMAN	Homo sapiens	248	K	Acetylation	PSP	NULL																
IPR000308-59	IPR000308	P31947-49	P31947	49	59	1433S_HUMAN	Homo sapiens	248	K	Ubiquitination	PSP	NULL																
IPR000308-59	IPR000308	P35213-51	P35213	51	59	1433B_RAT	Rattus norvegicus	246	K	Acetylation	PSP	NULL																
IPR000308-59	IPR000308	P35213-51	P35213	51	59	1433B_RAT	Rattus norvegicus	246	K	Ubiquitination	PSP	NULL																
IPR000308-59	IPR000308	P61981-50	P61981	50	59	1433G_HUMAN	Homo sapiens	247	K	Acetylation	PSP	NULL																
IPR000308-59	IPR000308	P61981-50	P61981	50	59	1433G_HUMAN	Homo sapiens	247	K	Methylation	PSP	NULL																
IPR000308-59	IPR000308	P61981-50	P61981	50	59	1433G_HUMAN	Homo sapiens	247	K	Ubiquitination	PSP	NULL																
IPR000308-59	IPR000308	P62259-50	P62259	50	59	1433E_MOUSE	Mus musculus	255	K	Ubiquitination	PSP	NULL																
IPR000308-59	IPR000308	P62260-50	P62260	50	59	1433E_RAT	Rattus norvegicus	255	K	Acetylation	PSP	NULL																
IPR000308-59	IPR000308	P62260-50	P62260	50	59	1433E_RAT	Rattus norvegicus	255	K	Ubiquitination	PSP	NULL																
IPR000308-59	IPR000308	P63101-49	P63101	49	59	1433Z_MOUSE	Mus musculus	245	K	Acetylation	PSP	NULL																
IPR000308-59	IPR000308	P63101-49	P63101	49	59	1433Z_MOUSE	Mus musculus	245	K	Ubiquitination	PSP	NULL																
IPR000308-59	IPR000308	P63102-49	P63102	49	59	1433Z_RAT	Rattus norvegicus	245	K	Acetylation	PSP	NULL																
IPR000308-59	IPR000308	P63102-49	P63102	49	59	1433Z_RAT	Rattus norvegicus	245	K	Ubiquitination	PSP	NULL																
IPR000308-59	IPR000308	P63104-49	P63104	49	59	1433Z_HUMAN	Homo sapiens	245	K	Acetylation	PSP	NULL																
IPR000308-59	IPR000308	P63104-49	P63104	49	59	1433Z_HUMAN	Homo sapiens	245	K	Methylation	PSP	NULL																
IPR000308-59	IPR000308	P63104-49	P63104	49	59	1433Z_HUMAN	Homo sapiens	245	K	Ubiquitination	PSP	NULL																
IPR000308-59	IPR000308	P68254-49	P68254	49	59	1433T_MOUSE	Mus musculus	245	K	Ubiquitination	PSP	NULL																
IPR000308-59	IPR000308	P68510-50	P68510	50	59	1433F_MOUSE	Mus musculus	246	K	Acetylation	PSP	NULL																
IPR000308-59	IPR000308	P68510-50	P68510	50	59	1433F_MOUSE	Mus musculus	246	K	Ubiquitination	PSP	NULL																
IPR000308-59	IPR000308	Q04917-50	Q04917	50	59	1433F_HUMAN	Homo sapiens	246	K	Acetylation	PSP	NULL																
IPR000308-59	IPR000308	Q04917-50	Q04917	50	59	1433F_HUMAN	Homo sapiens	246	K	Methylation	PSP	NULL																
IPR000308-59	IPR000308	Q04917-50	Q04917	50	59	1433F_HUMAN	Homo sapiens	246	K	Ubiquitination	PSP	NULL																
IPR000308-59	IPR000308	Q9CQV8-51	Q9CQV8	51	59	1433B_MOUSE	Mus musculus	246	K	Acetylation	PSP	NULL																
IPR000308-59	IPR000308	Q9CQV8-51	Q9CQV8	51	59	1433B_MOUSE	Mus musculus	246	K	Ubiquitination	PSP	NULL																
IPR000308-67	IPR000308	P27348-57	P27348	57	67	1433T_HUMAN	Homo sapiens	245	S	Phosphorylation	PSP	NULL																
IPR000308-67	IPR000308	P31946-59	P31946	59	67	1433B_HUMAN	Homo sapiens	246	S	Phosphorylation	PSP	NULL																
IPR000308-67	IPR000308	P63102-57	P63102	57	67	1433Z_RAT	Rattus norvegicus	245	S	Phosphorylation	PSP	NULL																
IPR000308-67	IPR000308	P63104-57	P63104	57	67	1433Z_HUMAN	Homo sapiens	245	S	Phosphorylation	PSP	NULL																
IPR000308-68	IPR000308	P35213-60	P35213	60	68	1433B_RAT	Rattus norvegicus	246	S	Phosphorylation	PSP	NULL	S	p	Ywhab	14-3-3 beta	Adaptor/scaffold	56011	3q42	rat	450226	VVGARRSsWRVISSI	14-3-3	intracellular localization	transcription, altered		12619878	Double mutant inhibited a MEF2 reporter and promotes nuclear localization of HDAC4/GFP.
IPR000308-68	IPR000308	P61981-59	P61981	59	68	1433G_HUMAN	Homo sapiens	247	S	Phosphorylation	PSP	NULL	S	p	YWHAG	14-3-3 gamma	Adaptor/scaffold	7532	7q11.23	human	3892745	VVGARRSsWRVISSI	14-3-3	molecular association, regulation; intracellular localization		TNRC6A(INDUCES)	21118956	localizes to P-bodies
IPR000308-68	IPR000308	P61982-59	P61982	59	68	1433G_MOUSE	Mus musculus	247	S	Phosphorylation	PSP	NULL	S	p	Ywhag	14-3-3 gamma	Adaptor/scaffold	22628	5|5 G2	mouse	3892745	VVGARRSsWRVISSI	14-3-3	molecular association, regulation		14-3-3 gamma(INDUCES); STAR(INDUCES)	25086053	
IPR000308-68	IPR000308	P63101-58	P63101	58	68	1433Z_MOUSE	Mus musculus	245	S	Phosphorylation	PSP	NULL	S	p	Ywhaz	14-3-3 zeta	Adaptor/scaffold; Motility/polarity/chemotaxis	22631	15|15 B3.1	mouse	449833	VVGARRSsWRVVSSI	14-3-3	molecular association, regulation	apoptosis, induced	14-3-3 zeta(DISRUPTS)	12865427; 20813501	
IPR000308-68	IPR000308	P63104-58	P63104	58	68	1433Z_HUMAN	Homo sapiens	245	S	Phosphorylation	PSP	NULL	S	p	YWHAZ	14-3-3 zeta	Adaptor/scaffold; Motility/polarity/chemotaxis	7534	8q22.3	human	449833	VVGARRSsWRVVSSI	14-3-3	protein degradation; molecular association, regulation	apoptosis, altered	14-3-3 zeta(DISRUPTS); 14-3-3 zeta(INDUCES); Tau iso3(DISRUPTS); ASK1(DISRUPTS); 14-3-3 epsilon(DISRUPTS); HSP20(DISRUPTS); p53(DISRUPTS)	11956222; 16376338; 18559254; 19451227; 21081103	
IPR000308-68	IPR000308	P31946-60	P31946	60	68	1433B_HUMAN	Homo sapiens	246	S	Phosphorylation	PSP	NULL																
IPR000308-68	IPR000308	P62258-59	P62258	59	68	1433E_HUMAN	Homo sapiens	255	S	Phosphorylation	PSP	NULL																
IPR000308-68	IPR000308	P63102-58	P63102	58	68	1433Z_RAT	Rattus norvegicus	245	S	Phosphorylation	PSP	NULL																
IPR000308-68	IPR000308	P68510-59	P68510	59	68	1433F_MOUSE	Mus musculus	246	S	Phosphorylation	PSP	NULL																
IPR000308-68	IPR000308	Q04917-59	Q04917	59	68	1433F_HUMAN	Homo sapiens	246	S	Phosphorylation	PSP	NULL																
IPR000308-68	IPR000308	Q9CQV8-60	Q9CQV8	60	68	1433B_MOUSE	Mus musculus	246	S	Phosphorylation	PSP	NULL																
IPR000308-73	IPR000308	P35213-65	P35213	65	73	1433B_RAT	Rattus norvegicus	246	S	Phosphorylation	PSP	NULL	S	p	Ywhab	14-3-3 beta	Adaptor/scaffold	56011	3q42	rat	467269	RSSWRVIsSIEQKTE	14-3-3	intracellular localization	transcription, altered		12619878	Double mutant inhibited a MEF2 reporter and promotes nuclear localization of HDAC4/GFP.
IPR000308-73	IPR000308	O70456-63	O70456	63	73	1433S_MOUSE	Mus musculus	248	S	Phosphorylation	PSP	NULL																
IPR000308-73	IPR000308	P29311-65	P29311	65	73	BMH1_YEAST	Saccharomyces cerevisiae	267	S	Phosphorylation	SysPTM 1.1	17287358																
IPR000308-73	IPR000308	P31947-63	P31947	63	73	1433S_HUMAN	Homo sapiens	248	S	Phosphorylation	PSP	NULL																
IPR000308-73	IPR000308	P42656-66	P42656	66	73	RAD24_SCHPO	Schizosaccharomyces pombe	270	S	Phosphorylation	Uniport_201509	18257517																
IPR000308-73	IPR000308	P61982-64	P61982	64	73	1433G_MOUSE	Mus musculus	247	S	Phosphorylation	PSP	NULL																
IPR000308-73	IPR000308	P62258-64	P62258	64	73	1433E_HUMAN	Homo sapiens	255	S	Phosphorylation	PSP	NULL																
IPR000308-73	IPR000308	P63104-63	P63104	63	73	1433Z_HUMAN	Homo sapiens	245	S	Phosphorylation	PSP	NULL																
IPR000308-73	IPR000308	P68254-63	P68254	63	73	1433T_MOUSE	Mus musculus	245	S	Phosphorylation	PSP	NULL																
IPR000308-73	IPR000308	P68510-64	P68510	64	73	1433F_MOUSE	Mus musculus	246	S	Phosphorylation	PSP	NULL																
IPR000308-73	IPR000308	Q9CQV8-65	Q9CQV8	65	73	1433B_MOUSE	Mus musculus	246	S	Phosphorylation	PSP	NULL																
IPR000308-74	IPR000308	O70456-64	O70456	64	74	1433S_MOUSE	Mus musculus	248	S	Phosphorylation	PSP	NULL																
IPR000308-74	IPR000308	P27348-64	P27348	64	74	1433T_HUMAN	Homo sapiens	245	S	Phosphorylation	PSP	NULL																

IPR000308-74	IPR000308	P29311-66	P29311	66	74	BMH1_YEAST	Saccharomyces cerevisiae	267	S	Phosphorylation	SysPTM 1.1	17287358													
IPR000308-74	IPR000308	P31946-66	P31946	66	74	1433B_HUMAN	Homo sapiens	246	S	Phosphorylation	PSP	NULL													
IPR000308-74	IPR000308	P31947-64	P31947	64	74	1433S_HUMAN	Homo sapiens	248	S	Phosphorylation	PSP	NULL													
IPR000308-74	IPR000308	P61981-65	P61981	65	74	1433G_HUMAN	Homo sapiens	247	S	Phosphorylation	PSP	NULL													
IPR000308-74	IPR000308	P61982-65	P61982	65	74	1433G_MOUSE	Mus musculus	247	S	Phosphorylation	PSP	NULL													
IPR000308-74	IPR000308	P62258-65	P62258	65	74	1433E_HUMAN	Homo sapiens	255	S	Phosphorylation	PSP	NULL													
IPR000308-74	IPR000308	P62260-65	P62260	65	74	1433E_RAT	Rattus norvegicus	255	S	Phosphorylation	PSP	NULL													
IPR000308-74	IPR000308	P63101-64	P63101	64	74	1433Z_MOUSE	Mus musculus	245	S	Phosphorylation	PSP	NULL													
IPR000308-74	IPR000308	P63104-64	P63104	64	74	1433Z_HUMAN	Homo sapiens	245	S	Phosphorylation	PSP	NULL													
IPR000308-74	IPR000308	P68254-64	P68254	64	74	1433T_MOUSE	Mus musculus	245	S	Phosphorylation	PSP	NULL													
IPR000308-74	IPR000308	P68510-65	P68510	65	74	1433F_MOUSE	Mus musculus	246	S	Phosphorylation	PSP	NULL													
IPR000308-74	IPR000308	Q04917-65	Q04917	65	74	1433F_HUMAN	Homo sapiens	246	S	Phosphorylation	PSP	NULL													
IPR000308-74	IPR000308	Q20655-66	Q20655	66	74	14332_CAEEL	Caenorhabditis elegans	248	S	Phosphorylation	PhosphoELM.10011	19530675													
IPR000308-74	IPR000308	Q9CQV8-66	Q9CQV8	66	74	1433B_MOUSE	Mus musculus	246	S	Phosphorylation	PSP	NULL													
IPR000308-78	IPR000308	P27348-68	P27348	68	78	1433T_HUMAN	Homo sapiens	245	K	Acetylation	PSP	NULL													
IPR000308-78	IPR000308	P27348-68	P27348	68	78	1433T_HUMAN	Homo sapiens	245	K	Ubiquitination	PSP	NULL													
IPR000308-78	IPR000308	P31946-70	P31946	70	78	1433B_HUMAN	Homo sapiens	246	K	Acetylation	PSP	NULL													
IPR000308-78	IPR000308	P31946-70	P31946	70	78	1433B_HUMAN	Homo sapiens	246	K	Ubiquitination	PSP	NULL													
IPR000308-78	IPR000308	P31947-68	P31947	68	78	1433S_HUMAN	Homo sapiens	248	K	Ubiquitination	PSP	NULL													
IPR000308-78	IPR000308	P35213-70	P35213	70	78	1433B_RAT	Rattus norvegicus	246	K	Acetylation	PSP	NULL													
IPR000308-78	IPR000308	P35213-70	P35213	70	78	1433B_RAT	Rattus norvegicus	246	K	Ubiquitination	PSP	NULL													
IPR000308-78	IPR000308	P61981-69	P61981	69	78	1433G_HUMAN	Homo sapiens	247	K	Acetylation	PSP	NULL													
IPR000308-78	IPR000308	P61981-69	P61981	69	78	1433G_HUMAN	Homo sapiens	247	K	Ubiquitination	PSP	NULL													
IPR000308-78	IPR000308	P61982-69	P61982	69	78	1433G_MOUSE	Mus musculus	247	K	Acetylation	PSP	NULL													
IPR000308-78	IPR000308	P61982-69	P61982	69	78	1433G_MOUSE	Mus musculus	247	K	Ubiquitination	PSP	NULL													
IPR000308-78	IPR000308	P62258-69	P62258	69	78	1433E_HUMAN	Homo sapiens	255	K	Acetylation	PSP	NULL													
IPR000308-78	IPR000308	P62258-69	P62258	69	78	1433E_HUMAN	Homo sapiens	255	K	Ubiquitination	PSP	NULL													
IPR000308-78	IPR000308	P62259-69	P62259	69	78	1433E_MOUSE	Mus musculus	255	K	Acetylation	PSP	NULL													
IPR000308-78	IPR000308	P62259-69	P62259	69	78	1433E_MOUSE	Mus musculus	255	K	Ubiquitination	PSP	NULL													
IPR000308-78	IPR000308	P62260-69	P62260	69	78	1433E_RAT	Rattus norvegicus	255	K	Acetylation	PSP	NULL													
IPR000308-78	IPR000308	P63101-68	P63101	68	78	1433Z_MOUSE	Mus musculus	245	K	Acetylation	PSP	NULL													
IPR000308-78	IPR000308	P63101-68	P63101	68	78	1433Z_MOUSE	Mus musculus	245	K	Ubiquitination	PSP	NULL													
IPR000308-78	IPR000308	P63102-68	P63102	68	78	1433Z_RAT	Rattus norvegicus	245	K	Acetylation	PSP	NULL													
IPR000308-78	IPR000308	P63104-68	P63104	68	78	1433Z_HUMAN	Homo sapiens	245	K	Acetylation	PSP	NULL													
IPR000308-78	IPR000308	P63104-68	P63104	68	78	1433Z_HUMAN	Homo sapiens	245	K	Ubiquitination	PSP	NULL													
IPR000308-78	IPR000308	P68254-68	P68254	68	78	1433T_MOUSE	Mus musculus	245	K	Ubiquitination	PSP	NULL													
IPR000308-78	IPR000308	P68510-69	P68510	69	78	1433F_MOUSE	Mus musculus	246	K	Ubiquitination	PSP	NULL													
IPR000308-78	IPR000308	Q04917-69	Q04917	69	78	1433F_HUMAN	Homo sapiens	246	K	Acetylation	PSP	NULL													
IPR000308-78	IPR000308	Q04917-69	Q04917	69	78	1433F_HUMAN	Homo sapiens	246	K	Ubiquitination	PSP	NULL													
IPR000308-78	IPR000308	Q9CQV8-70	Q9CQV8	70	78	1433B_MOUSE	Mus musculus	246	K	Ubiquitination	PSP	NULL													
IPR000308-79	IPR000308	P31947-69	P31947	69	79	1433S_HUMAN	Homo sapiens	248	S	Phosphorylation	PSP	NULL	S	p	SFN	14-3-3 sigma	Adaptor/scaffold	2810	1p36.11	human	454996	LSSIEQKsNEEGSEE	14-3-3	carcinogenesis, induced; transcription, induced	23741479
IPR000308-79	IPR000308	O70456-69	O70456	69	79	1433S_MOUSE	Mus musculus	248	S	Phosphorylation	PSP	NULL													
IPR000308-80	IPR000308	P61981-71	P61981	71	80	1433G_HUMAN	Homo sapiens	247	S	Phosphorylation	PSP	NULL													
IPR000308-80	IPR000308	P61982-71	P61982	71	80	1433G_MOUSE	Mus musculus	247	S	Phosphorylation	PSP	NULL													
IPR000308-82	IPR000308	P62258-73	P62258	73	82	1433E_HUMAN	Homo sapiens	255	K	Ubiquitination	PSP	NULL													
IPR000308-82	IPR000308	P62259-73	P62259	73	82	1433E_MOUSE	Mus musculus	255	K	Ubiquitination	PSP	NULL													
IPR000308-82	IPR000308	P62260-73	P62260	73	82	1433E_RAT	Rattus norvegicus	255	K	Acetylation	PSP	NULL													
IPR000308-85	IPR000308	P31947-74	P31947	74	85	1433S_HUMAN	Homo sapiens	248	S	Phosphorylation	PSP	NULL	S	p	SFN	14-3-3 sigma	Adaptor/scaffold	2810	1p36.11	human	454997	QKSNEEGsEEKGPEV	14-3-3	carcinogenesis, induced; transcription, induced	23741479
IPR000308-85	IPR000308	O70456-74	O70456	74	85	1433S_MOUSE	Mus musculus	248	S	Phosphorylation	PSP	NULL													
IPR000308-87	IPR000308	P27348-74	P27348	74	87	1433T_HUMAN	Homo sapiens	245	K	Ubiquitination	PSP	NULL													
IPR000308-87	IPR000308	P61981-77	P61981	77	87	1433G_HUMAN	Homo sapiens	247	K	Ubiquitination	PSP	NULL													
IPR000308-87	IPR000308	P61982-77	P61982	77	87	1433G_MOUSE	Mus musculus	247	K	Ubiquitination	PSP	NULL													
IPR000308-87	IPR000308	P63101-74	P63101	74	87	1433Z_MOUSE	Mus musculus	245	K	Ubiquitination	PSP	NULL													
IPR000308-87	IPR000308	P63102-74	P63102	74	87	1433Z_RAT	Rattus norvegicus	245	K	Acetylation	PSP	NULL													
IPR000308-87	IPR000308	P63104-74	P63104	74	87	1433Z_HUMAN	Homo sapiens	245	K	Acetylation	PSP	NULL													
IPR000308-87	IPR000308	P68254-74	P68254	74	87	1433T_MOUSE	Mus musculus	245	K	Ubiquitination	PSP	NULL													
IPR000308-87	IPR000308	P68510-77	P68510	77	87	1433F_MOUSE	Mus musculus	246	K	Acetylation	PSP	NULL													
IPR000308-87	IPR000308	P68510-77	P68510	77	87	1433F_MOUSE	Mus musculus	246	K	Ubiquitination	PSP	NULL													
IPR000308-87	IPR000308	Q04917-77	Q04917	77	87	1433F_HUMAN	Homo sapiens	246	K	Ubiquitination	PSP	NULL													
IPR000308-88	IPR000308	P27348-75	P27348	75	88	1433T_HUMAN	Homo sapiens	245	K	Ubiquitination	PSP	NULL													
IPR000308-88	IPR000308	P31946-77	P31946	77	88	1433B_HUMAN	Homo sapiens	246	K	Acetylation	PSP	NULL													
IPR000308-88	IPR000308	P31947-77	P31947	77	88	1433S_HUMAN	Homo sapiens	248	K	Ubiquitination	PSP	NULL													
IPR000308-88	IPR000308	P61982-78	P61982	78	88	1433G_MOUSE	Mus musculus	247	K	Acetylation	PSP	NULL													
IPR000308-88	IPR000308	P62258-78	P62258	78	88	1433E_HUMAN	Homo sapiens	255	K	Acetylation	PSP	NULL													
IPR000308-88	IPR000308	P62259-78	P62259	78	88	1433E_MOUSE	Mus musculus	255	K	Ubiquitination	PSP	NULL													
IPR000308-88	IPR000308	P62260-78	P62260	78	88	1433E_RAT	Rattus norvegicus	255	K	Acetylation	PSP	NULL													
IPR000308-88	IPR000308	P63104-75	P63104	75	88	1433Z_HUMAN	Homo sapiens	245	K	Acetylation	PSP	NULL													
IPR000308-90	IPR000308	P62258-80	P62258	80	90	1433E_HUMAN	Homo sapiens	255	K	Ubiquitination	PSP	NULL													
IPR000308-90	IPR000308	P62259-80	P62259	80	90	1433E_MOUSE	Mus musculus	255	K	Ubiquitination	PSP	NULL													
IPR000308-93	IPR000308	P27348-80	P27348	80	93	1433T_HUMAN	Homo sapiens	245	K	Ubiquitination	PSP	NULL													
IPR000308-93	IPR000308	P68254-80	P68254	80	93	1433T_MOUSE	Mus musculus	245	K	Ubiquitination	PSP	NULL													
IPR000308-94	IPR000308	P29311-86	P29311	86	94	BMH1_YEAST	Saccharomyces cerevisiae	267	S	Phosphorylation	SysPTM 1.1	17287358													
IPR000308-95	IPR000308	P27348-82	P27348	82	95	1433T_HUMAN	Homo sapiens	245	Y	Phosphorylation	PSP	NULL													
IPR000308-95	IPR000308	P63104-82	P63104	82	95	1433Z_HUMAN	Homo sapiens	245	Y	Phosphorylation	PSP	NULL													
IPR000308-95	IPR000308	Q9CQV8-84	Q9CQV8	84	95	1433B_MOUSE	Mus musculus	246	Y	Nitration	Uniport_201509	16800626													
IPR000308-97	IPR000308	P29311-89	P29311	89	97	BMH1_YEAST	Saccharomyces cerevisiae	267	S	Phosphorylation	SysPTM 1.1	17287358													
IPR000308-98	IPR000308	P27348-85	P27348	85	98	1433T_HUMAN	Homo sapiens	245	K	Acetylation	PSP	NULL													
IPR000308-98	IPR000308	P27348-85	P27348	85	98	1433T_HUMAN	Homo sapiens	245	K	Ubiquitination	PSP	NULL													
IPR000308-98	IPR000308	P61982-88	P61982	88	98	1433G_MOUSE	Mus musculus	247	K	Acetylation	PSP	NULL													
IPR000308-98	IPR000308	P63101-85	P63101	85	98	1433Z_MOUSE	Mus musculus	245	K	Ubiquitination	PSP	NULL													
IPR000308-98	IPR000308	P63104-85	P63104	85	98	1433Z_HUMAN	Homo sapiens	245	K	Acetylation	PSP	NULL													
IPR000308-98	IPR000308	P63104-85	P63104	85	98	1433Z_HUMAN	Homo sapiens	245	K	Methylation	PSP	NULL													
IPR000308-98	IPR000308	P63104-85	P63104	85	98	1433Z_HUMAN	Homo sapiens	245	K	Ubiquitination	PSP	NULL													
IPR000308-98	IPR000308	P68254-85	P68254	85	98	1433T_MOUSE	Mus musculus	245	K	Ubiquitination	PSP	NULL													
IPR000308-101	IPR000308	O70456-90	O70456	90	101	1433S_MOUSE	Mus musculus	248	T	Phosphorylation	PSP	NULL													
IPR000308-101	IPR000308	P27348-88	P27348	88	101	1433T_HUMAN	Homo sapiens	245	S	Phosphorylation	PSP	NULL													

IPR000308-101	IPR000308	P61982-91	P61982	91	101	1433G_MOUSE	Mus musculus	247	K	Acetylation	PSP	NULL															
IPR000308-101	IPR000308	P62258-91	P62258	91	101	1433E_HUMAN	Homo sapiens	255	T	Phosphorylation	PSP	NULL															
IPR000308-101	IPR000308	P63104-88	P63104	88	101	1433Z_HUMAN	Homo sapiens	245	T	Phosphorylation	PSP	NULL															
IPR000308-101	IPR000308	P68254-88	P68254	88	101	1433T_MOUSE	Mus musculus	245	S	Phosphorylation	PSP	NULL															
IPR000308-105	IPR000308	P27348-92	P27348	92	105	1433T_HUMAN	Homo sapiens	245	S	Phosphorylation	PSP	NULL															
IPR000308-105	IPR000308	P68254-92	P68254	92	105	1433T_MOUSE	Mus musculus	245	S	Phosphorylation	PSP	NULL															
IPR000308-107	IPR000308	P27348-94	P27348	94	107	1433T_HUMAN	Homo sapiens	245	C	S-Nitrosylation	dbSNOv2	19483679															
IPR000308-107	IPR000308	P48347-98	P48347	98	107	14310_ARATH	Arabidopsis thaliana	254	C	S-Nitrosylation	dbSNOv2	15734904															
IPR000308-107	IPR000308	P62258-97	P62258	97	107	1433E_HUMAN	Homo sapiens	255	C	S-Nitrosylation	dbSNOv2	19483679															
IPR000308-107	IPR000308	P63101-94	P63101	94	107	1433Z_MOUSE	Mus musculus	245	C	S-Nitrosylation	dbSNOv2	21278135															
IPR000308-107	IPR000308	P63102-94	P63102	94	107	1433Z_RAT	Rattus norvegicus	245	C	S-Nitrosylation	dbSNOv2	16418269															
IPR000308-107	IPR000308	P63104-94	P63104	94	107	1433Z_HUMAN	Homo sapiens	245	C	S-Nitrosylation	dbSNOv2	19483679															
IPR000308-107	IPR000308	P68254-94	P68254	94	107	1433T_MOUSE	Mus musculus	245	C	S-Nitrosylation	dbSNOv2	22588120															
IPR000308-108	IPR000308	P27348-95	P27348	95	108	1433T_HUMAN	Homo sapiens	245	T	Phosphorylation	HPRD 9.0	20068231															
IPR000308-108	IPR000308	P62258-98	P62258	98	108	1433E_HUMAN	Homo sapiens	255	C	S-Nitrosylation	dbSNOv2	19483679															
IPR000308-108	IPR000308	P62259-98	P62259	98	108	1433E_MOUSE	Mus musculus	255	C	S-Nitrosylation	dbSNOv2	20925432															
IPR000308-109	IPR000308	O70456-98	O70456	98	109	1433S_MOUSE	Mus musculus	248	T	Phosphorylation	PSP	NULL															
IPR000308-112	IPR000308	P34730-104	P34730	104	112	BMH2_YEAST	Saccharomyces cerevisiae	273	S	Phosphorylation	SysPTM 1.1	18407956															
IPR000308-112	IPR000308	P63101-99	P63101	99	112	1433Z_MOUSE	Mus musculus	245	S	Phosphorylation	PSP	NULL															
IPR000308-112	IPR000308	P63104-99	P63104	99	112	1433Z_HUMAN	Homo sapiens	245	S	Phosphorylation	PSP	NULL															
IPR000308-116	IPR000308	P31946-105	P31946	105	116	1433B_HUMAN	Homo sapiens	246	K	Ubiquitination	PSP	NULL															
IPR000308-116	IPR000308	P62258-106	P62258	106	116	1433E_HUMAN	Homo sapiens	255	K	Acetylation	PSP	NULL															
IPR000308-116	IPR000308	P62258-106	P62258	106	116	1433E_HUMAN	Homo sapiens	255	K	Ubiquitination	PSP	NULL															
IPR000308-116	IPR000308	P62259-106	P62259	106	116	1433E_MOUSE	Mus musculus	255	K	Acetylation	PSP	NULL															
IPR000308-116	IPR000308	P62259-106	P62259	106	116	1433E_MOUSE	Mus musculus	255	K	Ubiquitination	PSP	NULL															
IPR000308-116	IPR000308	P62260-106	P62260	106	116	1433E_RAT	Rattus norvegicus	255	K	Ubiquitination	PSP	NULL															
IPR000308-116	IPR000308	P63104-103	P63104	103	116	1433Z_HUMAN	Homo sapiens	245	K	Ubiquitination	PSP	NULL															
IPR000308-117	IPR000308	P31946-106	P31946	106	117	1433B_HUMAN	Homo sapiens	246	Y	Phosphorylation	PSP	NULL															
IPR000308-117	IPR000308	Q9CQV8-106	Q9CQV8	106	117	1433B_MOUSE	Mus musculus	246	Y	Nitration	Uniport_201509	16800626															
IPR000308-120	IPR000308	O70456-109	O70456	109	120	1433S_MOUSE	Mus musculus	248	K	Ubiquitination	PSP	NULL															
IPR000308-120	IPR000308	P31947-109	P31947	109	120	1433S_HUMAN	Homo sapiens	248	K	Ubiquitination	PSP	NULL															
IPR000308-120	IPR000308	P61981-110	P61981	110	120	1433G_HUMAN	Homo sapiens	247	K	Ubiquitination	PSP	NULL															
IPR000308-120	IPR000308	P68510-110	P68510	110	120	1433F_MOUSE	Mus musculus	246	K	Ubiquitination	PSP	NULL															
IPR000308-120	IPR000308	Q04917-110	Q04917	110	120	1433F_HUMAN	Homo sapiens	246	K	Ubiquitination	PSP	NULL															
IPR000308-122	IPR000308	P61982-112	P61982	112	122	1433G_MOUSE	Mus musculus	247	C	S-Nitrosylation	dbSNOv2	21278135															
IPR000308-122	IPR000308	P68510-112	P68510	112	122	1433F_MOUSE	Mus musculus	246	C	S-Nitrosylation	dbSNOv2	19101475															
IPR000308-123	IPR000308	P27348-110	P27348	110	123	1433T_HUMAN	Homo sapiens	245	T	Phosphorylation	HPRD 9.0	20068231															
IPR000308-123	IPR000308	P61982-113	P61982	113	123	1433G_MOUSE	Mus musculus	247	S	Phosphorylation	PSP	NULL															
IPR000308-123	IPR000308	P63101-110	P63101	110	123	1433Z_MOUSE	Mus musculus	245	S	Phosphorylation	PSP	NULL															
IPR000308-123	IPR000308	P63104-110	P63104	110	123	1433Z_HUMAN	Homo sapiens	245	S	Phosphorylation	PSP	NULL															
IPR000308-123	IPR000308	P68254-110	P68254	110	123	1433T_MOUSE	Mus musculus	245	T	Phosphorylation	PSP	NULL															
IPR000308-124	IPR000308	P62258-114	P62258	114	124	1433E_HUMAN	Homo sapiens	255	T	Phosphorylation	PSP	NULL															
IPR000308-125	IPR000308	P61981-115	P61981	115	125	1433G_HUMAN	Homo sapiens	247	T	Phosphorylation	PSP	NULL															
IPR000308-127	IPR000308	P61981-117	P61981	117	127	1433G_HUMAN	Homo sapiens	247	Y	Phosphorylation	PSP	NULL															
IPR000308-127	IPR000308	P61982-117	P61982	117	127	1433G_MOUSE	Mus musculus	247	Y	Phosphorylation	SysPTM 1.1	18034455															
IPR000308-127	IPR000308	P68510-117	P68510	117	127	1433F_MOUSE	Mus musculus	246	Y	Phosphorylation	PSP	NULL															
IPR000308-129	IPR000308	O70456-116	O70456	116	129	1433S_MOUSE	Mus musculus	248	S	Phosphorylation	PSP	NULL															
IPR000308-129	IPR000308	P27348-114	P27348	114	129	1433T_HUMAN	Homo sapiens	245	S	Phosphorylation	PSP	NULL															
IPR000308-129	IPR000308	P31946-116	P31946	116	129	1433B_HUMAN	Homo sapiens	246	S	Phosphorylation	PSP	NULL															
IPR000308-129	IPR000308	P31947-116	P31947	116	129	1433S_HUMAN	Homo sapiens	248	S	Phosphorylation	PSP	NULL															
IPR000308-129	IPR000308	P61981-119	P61981	119	129	1433G_HUMAN	Homo sapiens	247	S	Phosphorylation	PSP	NULL															
IPR000308-129	IPR000308	P61982-119	P61982	119	129	1433G_MOUSE	Mus musculus	247	S	Phosphorylation	PSP	NULL															
IPR000308-129	IPR000308	P62258-117	P62258	117	129	1433E_HUMAN	Homo sapiens	255	S	Phosphorylation	PSP	NULL															
IPR000308-129	IPR000308	P63101-114	P63101	114	129	1433Z_MOUSE	Mus musculus	245	S	Phosphorylation	PSP	NULL															
IPR000308-129	IPR000308	P63104-114	P63104	114	129	1433Z_HUMAN	Homo sapiens	245	S	Phosphorylation	PSP	NULL															
IPR000308-129	IPR000308	Q04917-119	Q04917	119	129	1433F_HUMAN	Homo sapiens	246	S	Phosphorylation	PSP	NULL															
IPR000308-129	IPR000308	Q9CQV8-116	Q9CQV8	116	129	1433B_MOUSE	Mus musculus	246	S	Phosphorylation	PSP	NULL															
IPR000308-130	IPR000308	P62258-118	P62258	118	130	1433E_HUMAN	Homo sapiens	255	K	Acetylation	PSP	NULL	K	ac	YWHAE	14-3-3 epsilon	Adaptor/scaffold	7531	17p13.3	human	474724	AANTGESkVFYYKMK	14-3-3	molecular association, regulation		SFRS6(DISRUPTS);<br>PLEKHQ1(DISRUPTS);<br>SFRS4(DISRUPTS);<br>DOK3(DISRUPTS);<br>TSC2(DISRUPTS);<br>ARAF(DISRUPTS);<br>LARP(DISRUPTS);<br>SRm300(DISRUPTS);<br>SF2(DISRUPTS);<br>TSC22D1(DISRUPTS);<br>RAF1(DISRUPTS);<br>GAB2(DISRUPTS);<br>VASP(DISRUPTS);<br>HDAC4(DISRUPTS);<br>TRA2B(DISRUPTS);<br>GBF1(DISRUPTS)	19608861
IPR000308-130	IPR000308	P62258-118	P62258	118	130	1433E_HUMAN	Homo sapiens	255	K	Ubiquitination	PSP	NULL	K	ac	YWHAE	14-3-3 epsilon	Adaptor/scaffold	7531	17p13.3	human	474724	AANTGESkVFYYKMK	14-3-3	molecular association, regulation		SFRS6(DISRUPTS);<br>PLEKHQ1(DISRUPTS);<br>SFRS4(DISRUPTS);<br>DOK3(DISRUPTS);<br>TSC2(DISRUPTS);<br>ARAF(DISRUPTS);<br>LARP(DISRUPTS);<br>SRm300(DISRUPTS);<br>SF2(DISRUPTS);<br>TSC22D1(DISRUPTS);<br>RAF1(DISRUPTS);<br>GAB2(DISRUPTS);<br>VASP(DISRUPTS);<br>HDAC4(DISRUPTS);<br>TRA2B(DISRUPTS);<br>GBF1(DISRUPTS)	19608861
IPR000308-130	IPR000308	P27348-115	P27348	115	130	1433T_HUMAN	Homo sapiens	245	K	Acetylation	PSP	NULL															
IPR000308-130	IPR000308	P27348-115	P27348	115	130	1433T_HUMAN	Homo sapiens	245	K	Ubiquitination	PSP	NULL															
IPR000308-130	IPR000308	P31946-117	P31946	117	130	1433B_HUMAN	Homo sapiens	246	K	Acetylation	PSP	NULL															
IPR000308-130	IPR000308	P31946-117	P31946	117	130	1433B_HUMAN	Homo sapiens	246	K	Ubiquitination	PSP	NULL															
IPR000308-130	IPR000308	P61981-120	P61981	120	130	1433G_HUMAN	Homo sapiens	247	K	Acetylation	PSP	NULL															
IPR000308-130	IPR000308	P61981-120	P61981	120	130	1433G_HUMAN	Homo sapiens	247	K	Ubiquitination	PSP	NULL															

IPR000308-130	IPR000308	P61982-120	P61982	120	130	1433G_MOUSE	Mus musculus	247	K	Ubiquitination	PSP	NULL														
IPR000308-130	IPR000308	P62259-118	P62259	118	130	1433E_MOUSE	Mus musculus	255	K	Acetylation	PSP	NULL														
IPR000308-130	IPR000308	P62259-118	P62259	118	130	1433E_MOUSE	Mus musculus	255	K	Ubiquitination	PSP	NULL														
IPR000308-130	IPR000308	P62260-118	P62260	118	130	1433E_RAT	Rattus norvegicus	255	K	Acetylation	PSP	NULL														
IPR000308-130	IPR000308	P63101-115	P63101	115	130	1433Z_MOUSE	Mus musculus	245	K	Acetylation	PSP	NULL														
IPR000308-130	IPR000308	P63101-115	P63101	115	130	1433Z_MOUSE	Mus musculus	245	K	Ubiquitination	PSP	NULL														
IPR000308-130	IPR000308	P63102-115	P63102	115	130	1433Z_RAT	Rattus norvegicus	245	K	Acetylation	PSP	NULL														
IPR000308-130	IPR000308	P63104-115	P63104	115	130	1433Z_HUMAN	Homo sapiens	245	K	Acetylation	PSP	NULL														
IPR000308-130	IPR000308	P63104-115	P63104	115	130	1433Z_HUMAN	Homo sapiens	245	K	Ubiquitination	PSP	NULL														
IPR000308-130	IPR000308	P68254-115	P68254	115	130	1433T_MOUSE	Mus musculus	245	K	Acetylation	PSP	NULL														
IPR000308-130	IPR000308	P68254-115	P68254	115	130	1433T_MOUSE	Mus musculus	245	K	Ubiquitination	PSP	NULL														
IPR000308-130	IPR000308	P68510-120	P68510	120	130	1433F_MOUSE	Mus musculus	246	K	Ubiquitination	PSP	NULL														
IPR000308-130	IPR000308	Q04917-120	Q04917	120	130	1433F_HUMAN	Homo sapiens	246	K	Acetylation	PSP	NULL														
IPR000308-133	IPR000308	P31946-120	P31946	120	133	1433B_HUMAN	Homo sapiens	246	Y	Phosphorylation	PSP	NULL														
IPR000308-133	IPR000308	Q04917-123	Q04917	123	133	1433F_HUMAN	Homo sapiens	246	Y	Phosphorylation	PSP	NULL														
IPR000308-134	IPR000308	P62258-122	P62258	122	134	1433E_HUMAN	Homo sapiens	255	Y	Phosphorylation	PSP	NULL														
IPR000308-135	IPR000308	P62258-123	P62258	123	135	1433E_HUMAN	Homo sapiens	255	K	Acetylation	PSP	NULL	K	ac	YWHAE	14-3-3 epsilon	Adaptor/scaffold	7531	17p13.3	human	11315832	ESKVFYYkMKGDYHR	14-3-3	molecular association, regulation	SFRS6(DISRUPTS); PLEKHQ1(DISRUPTS); SFRS4(DISRUPTS); DOK3(DISRUPTS); TSC2(DISRUPTS); ARAF(DISRUPTS); LARP(DISRUPTS); SRm300(DISRUPTS); SF2(DISRUPTS); TSC22D1(DISRUPTS); RAF1(DISRUPTS); GAB2(DISRUPTS); VASP(DISRUPTS); HDAC4(DISRUPTS); TRA2B(DISRUPTS); GBF1(DISRUPTS)	19608861
IPR000308-135	IPR000308	P62258-123	P62258	123	135	1433E_HUMAN	Homo sapiens	255	K	Ubiquitination	PSP	NULL	K	ac	YWHAE	14-3-3 epsilon	Adaptor/scaffold	7531	17p13.3	human	11315832	ESKVFYYkMKGDYHR	14-3-3	molecular association, regulation	SFRS6(DISRUPTS); PLEKHQ1(DISRUPTS); SFRS4(DISRUPTS); DOK3(DISRUPTS); TSC2(DISRUPTS); ARAF(DISRUPTS); LARP(DISRUPTS); SRm300(DISRUPTS); SF2(DISRUPTS); TSC22D1(DISRUPTS); RAF1(DISRUPTS); GAB2(DISRUPTS); VASP(DISRUPTS); HDAC4(DISRUPTS); TRA2B(DISRUPTS); GBF1(DISRUPTS)	19608861
IPR000308-135	IPR000308	O70456-122	O70456	122	135	1433S_MOUSE	Mus musculus	248	K	Acetylation	PSP	NULL														
IPR000308-135	IPR000308	O70456-122	O70456	122	135	1433S_MOUSE	Mus musculus	248	K	Ubiquitination	PSP	NULL														
IPR000308-135	IPR000308	P27348-120	P27348	120	135	1433T_HUMAN	Homo sapiens	245	K	Acetylation	PSP	NULL														
IPR000308-135	IPR000308	P27348-120	P27348	120	135	1433T_HUMAN	Homo sapiens	245	K	Ubiquitination	PSP	NULL														
IPR000308-135	IPR000308	P31946-122	P31946	122	135	1433B_HUMAN	Homo sapiens	246	K	Acetylation	PSP	NULL														
IPR000308-135	IPR000308	P31946-122	P31946	122	135	1433B_HUMAN	Homo sapiens	246	K	Ubiquitination	PSP	NULL														
IPR000308-135	IPR000308	P31947-122	P31947	122	135	1433S_HUMAN	Homo sapiens	248	K	Acetylation	PSP	NULL														
IPR000308-135	IPR000308	P31947-122	P31947	122	135	1433S_HUMAN	Homo sapiens	248	K	Ubiquitination	PSP	NULL														
IPR000308-135	IPR000308	P35213-122	P35213	122	135	1433B_RAT	Rattus norvegicus	246	K	Acetylation	PSP	NULL														
IPR000308-135	IPR000308	P35213-122	P35213	122	135	1433B_RAT	Rattus norvegicus	246	K	Ubiquitination	PSP	NULL														
IPR000308-135	IPR000308	P61981-125	P61981	125	135	1433G_HUMAN	Homo sapiens	247	K	Acetylation	PSP	NULL														
IPR000308-135	IPR000308	P61981-125	P61981	125	135	1433G_HUMAN	Homo sapiens	247	K	Ubiquitination	PSP	NULL														
IPR000308-135	IPR000308	P61982-125	P61982	125	135	1433G_MOUSE	Mus musculus	247	K	Acetylation	PSP	NULL														
IPR000308-135	IPR000308	P61982-125	P61982	125	135	1433G_MOUSE	Mus musculus	247	K	Ubiquitination	PSP	NULL														
IPR000308-135	IPR000308	P62259-123	P62259	123	135	1433E_MOUSE	Mus musculus	255	K	Ubiquitination	PSP	NULL														
IPR000308-135	IPR000308	P62260-123	P62260	123	135	1433E_RAT	Rattus norvegicus	255	K	Acetylation	PSP	NULL														
IPR000308-135	IPR000308	P63101-120	P63101	120	135	1433Z_MOUSE	Mus musculus	245	K	Acetylation	PSP	NULL														
IPR000308-135	IPR000308	P63101-120	P63101	120	135	1433Z_MOUSE	Mus musculus	245	K	Ubiquitination	PSP	NULL														
IPR000308-135	IPR000308	P63102-120	P63102	120	135	1433Z_RAT	Rattus norvegicus	245	K	Acetylation	PSP	NULL														
IPR000308-135	IPR000308	P63102-120	P63102	120	135	1433Z_RAT	Rattus norvegicus	245	K	Ubiquitination	PSP	NULL														
IPR000308-135	IPR000308	P63104-120	P63104	120	135	1433Z_HUMAN	Homo sapiens	245	K	Acetylation	PSP	NULL														
IPR000308-135	IPR000308	P63104-120	P63104	120	135	1433Z_HUMAN	Homo sapiens	245	K	Ubiquitination	PSP	NULL														
IPR000308-135	IPR000308	P68254-120	P68254	120	135	1433T_MOUSE	Mus musculus	245	K	Acetylation	PSP	NULL														
IPR000308-135	IPR000308	P68254-120	P68254	120	135	1433T_MOUSE	Mus musculus	245	K	Ubiquitination	PSP	NULL														
IPR000308-135	IPR000308	P68510-125	P68510	125	135	1433F_MOUSE	Mus musculus	246	K	Acetylation	PSP	NULL														
IPR000308-135	IPR000308	P68510-125	P68510	125	135	1433F_MOUSE	Mus musculus	246	K	Ubiquitination	PSP	NULL														
IPR000308-135	IPR000308	Q04917-125	Q04917	125	135	1433F_HUMAN	Homo sapiens	246	K	Acetylation	PSP	NULL														
IPR000308-135	IPR000308	Q04917-125	Q04917	125	135	1433F_HUMAN	Homo sapiens	246	K	Ubiquitination	PSP	NULL														
IPR000308-135	IPR000308	Q9CQV8-122	Q9CQV8	122	135	1433B_MOUSE	Mus musculus	246	K	Acetylation	PSP	NULL														
IPR000308-135	IPR000308	Q9CQV8-122	Q9CQV8	122	135	1433B_MOUSE	Mus musculus	246	K	Ubiquitination	PSP	NULL														
IPR000308-137	IPR000308	O70456-124	O70456	124	137	1433S_MOUSE	Mus musculus	248	K	Ubiquitination	PSP	NULL														
IPR000308-137	IPR000308	P27348-122	P27348	122	137	1433T_HUMAN	Homo sapiens	245	K	Ubiquitination	PSP	NULL														
IPR000308-137	IPR000308	P31946-124	P31946	124	137	1433B_HUMAN	Homo sapiens	246	K	Ubiquitination	PSP	NULL														
IPR000308-137	IPR000308	P31947-124	P31947	124	137	1433S_HUMAN	Homo sapiens	248	K	Ubiquitination	PSP	NULL														
IPR000308-137	IPR000308	P35213-124	P35213	124	137	1433B_RAT	Rattus norvegicus	246	K	Acetylation	PSP	NULL														
IPR000308-137	IPR000308	P61981-127	P61981	127	137	1433G_HUMAN	Homo sapiens	247	K	Ubiquitination	PSP	NULL														
IPR000308-137	IPR000308	P61982-127	P61982	127	137	1433G_MOUSE	Mus musculus	247	K	Ubiquitination	PSP	NULL														
IPR000308-137	IPR000308	P62258-125	P62258	125	137	1433E_HUMAN	Homo sapiens	255	K	Ubiquitination	PSP	NULL														
IPR000308-137	IPR000308	P62259-125	P62259	125	137	1433E_MOUSE	Mus musculus	255	K	Acetylation	PSP	NULL														
IPR000308-137	IPR000308	P62259-125	P62259	125	137	1433E_MOUSE	Mus musculus	255	K	Ubiquitination	PSP	NULL														
IPR000308-137	IPR000308	P62260-125	P62260	125	137	1433E_RAT	Rattus norvegicus	255	K	Acetylation	PSP	NULL														
IPR000308-137	IPR000308	P63101-122	P63101	122	137	1433Z_MOUSE	Mus musculus	245	K	Ubiquitination	PSP	NULL														
IPR000308-137	IPR000308	P63102-122	P63102	122	137	1433Z_RAT	Rattus norvegicus	245	K	Acetylation	PSP	NULL														
IPR000308-137	IPR000308	P63102-122	P63102	122	137	1433Z_RAT	Rattus norvegicus	245	K	Ubiquitination	PSP	NULL														
IPR000308-137	IPR000308	P63104-122	P63104	122	137	1433Z_HUMAN	Homo sapiens	245	K	Ubiquitination	PSP	NULL														
IPR000308-137	IPR000308	P68254-122	P68254	122	137	1433T_MOUSE	Mus musculus	245	K	Ubiquitination	PSP	NULL														
IPR000308-137	IPR000308	P68510-127	P68510	127	137	1433F_MOUSE	Mus musculus	246	K	Ubiquitination	PSP	NULL														
IPR000308-137	IPR000308	Q04917-127	Q04917	127	137	1433F_HUMAN	Homo sapiens	246	K	Ubiquitination	PSP	NULL														

IPR000308-137	IPR000308	Q9CQV8-124	Q9CQV8	124	137	1433B_MOUSE	Mus musculus	246	K	Ubiquitination	PSP	NULL														
IPR000308-140	IPR000308	P27348-125	P27348	125	140	1433T_HUMAN	Homo sapiens	245	Y	Phosphorylation	PSP	NULL														
IPR000308-140	IPR000308	P31946-127	P31946	127	140	1433B_HUMAN	Homo sapiens	246	Y	Phosphorylation	PSP	NULL														
IPR000308-140	IPR000308	P31947-127	P31947	127	140	1433S_HUMAN	Homo sapiens	248	Y	Phosphorylation	PSP	NULL														
IPR000308-140	IPR000308	P61981-130	P61981	130	140	1433G_HUMAN	Homo sapiens	247	Y	Phosphorylation	PSP	NULL														
IPR000308-140	IPR000308	P63104-125	P63104	125	140	1433Z_HUMAN	Homo sapiens	245	Y	Phosphorylation	PSP	NULL														
IPR000308-140	IPR000308	Q04917-130	Q04917	130	140	1433F_HUMAN	Homo sapiens	246	Y	Phosphorylation	PSP	NULL														
IPR000308-141	IPR000308	O70456-128	O70456	128	141	1433S_MOUSE	Mus musculus	248	Y	Phosphorylation	PSP	NULL														
IPR000308-141	IPR000308	P31947-128	P31947	128	141	1433S_HUMAN	Homo sapiens	248	Y	Phosphorylation	PSP	NULL														
IPR000308-141	IPR000308	P61981-131	P61981	131	141	1433G_HUMAN	Homo sapiens	247	Y	Phosphorylation	PSP	NULL														
IPR000308-141	IPR000308	P61982-131	P61982	131	141	1433G_MOUSE	Mus musculus	247	Y	Phosphorylation	PSP	NULL														
IPR000308-141	IPR000308	P63101-126	P63101	126	141	1433Z_MOUSE	Mus musculus	245	Y	Phosphorylation	PSP	NULL														
IPR000308-141	IPR000308	P63104-126	P63104	126	141	1433Z_HUMAN	Homo sapiens	245	Y	Phosphorylation	PSP	NULL														
IPR000308-141	IPR000308	P68510-131	P68510	131	141	1433F_MOUSE	Mus musculus	246	Y	Phosphorylation	PSP	NULL														
IPR000308-141	IPR000308	Q04917-131	Q04917	131	141	1433F_HUMAN	Homo sapiens	246	Y	Phosphorylation	PSP	NULL														
IPR000308-142	IPR000308	P62258-130	P62258	130	142	1433E_HUMAN	Homo sapiens	255	R	Methylation	PSP	NULL														
IPR000308-143	IPR000308	P27348-128	P27348	128	143	1433T_HUMAN	Homo sapiens	245	Y	Phosphorylation	PSP	NULL														
IPR000308-143	IPR000308	P31946-130	P31946	130	143	1433B_HUMAN	Homo sapiens	246	Y	Phosphorylation	PSP	NULL														
IPR000308-143	IPR000308	P61981-133	P61981	133	143	1433G_HUMAN	Homo sapiens	247	Y	Phosphorylation	PSP	NULL														
IPR000308-143	IPR000308	P61982-133	P61982	133	143	1433G_MOUSE	Mus musculus	247	Y	Phosphorylation	PSP	NULL														
IPR000308-143	IPR000308	P62258-131	P62258	131	143	1433E_HUMAN	Homo sapiens	255	Y	Phosphorylation	PSP	NULL														
IPR000308-143	IPR000308	P62259-131	P62259	131	143	1433E_MOUSE	Mus musculus	255	Y	Phosphorylation	PSP	NULL														
IPR000308-143	IPR000308	P62260-131	P62260	131	143	1433E_RAT	Rattus norvegicus	255	Y	Phosphorylation	PSP	NULL														
IPR000308-143	IPR000308	P63101-128	P63101	128	143	1433Z_MOUSE	Mus musculus	245	Y	Phosphorylation	PSP	NULL														
IPR000308-143	IPR000308	P63102-128	P63102	128	143	1433Z_RAT	Rattus norvegicus	245	Y	Phosphorylation	PSP	NULL														
IPR000308-143	IPR000308	P63104-128	P63104	128	143	1433Z_HUMAN	Homo sapiens	245	Y	Phosphorylation	PSP	NULL														
IPR000308-143	IPR000308	Q9CQV8-130	Q9CQV8	130	143	1433B_MOUSE	Mus musculus	246	Y	Phosphorylation	PSP	NULL														
IPR000308-145	IPR000308	P35213-132	P35213	132	145	1433B_RAT	Rattus norvegicus	246	S	Phosphorylation	PSP	NULL	S	p	Ywhab	14-3-3 beta	Adaptor/scaffold	56011	3q42	rat	3136105	GDYFRYLsEVASGDN	14-3-3	molecular association, regulation	RAF1(DISRUPTS); PKCZ(DISRUPTS)	10620507
IPR000308-145	IPR000308	P31946-132	P31946	132	145	1433B_HUMAN	Homo sapiens	246	S	Phosphorylation	PSP	NULL														
IPR000308-149	IPR000308	O70456-136	O70456	136	149	1433S_MOUSE	Mus musculus	248	T	Phosphorylation	PSP	NULL														
IPR000308-149	IPR000308	P27348-134	P27348	134	149	1433T_HUMAN	Homo sapiens	245	C	S-Nitrosylation	dbSNOv2	19101475														
IPR000308-149	IPR000308	P31946-136	P31946	136	149	1433B_HUMAN	Homo sapiens	246	S	Phosphorylation	PSP	NULL														
IPR000308-149	IPR000308	P31947-136	P31947	136	149	1433S_HUMAN	Homo sapiens	248	T	Phosphorylation	PSP	NULL														
IPR000308-149	IPR000308	P61981-139	P61981	139	149	1433G_HUMAN	Homo sapiens	247	T	Phosphorylation	PSP	NULL														
IPR000308-149	IPR000308	P62259-137	P62259	137	149	1433E_MOUSE	Mus musculus	255	T	Phosphorylation	PSP	NULL														
IPR000308-149	IPR000308	P62260-137	P62260	137	149	1433E_RAT	Rattus norvegicus	255	T	Phosphorylation	PSP	NULL														
IPR000308-149	IPR000308	P68254-134	P68254	134	149	1433T_MOUSE	Mus musculus	245	C	S-Nitrosylation	dbSNOv2	19101475														
IPR000308-149	IPR000308	Q04917-139	Q04917	139	149	1433F_HUMAN	Homo sapiens	246	S	Phosphorylation	PSP	NULL														
IPR000308-149	IPR000308	Q9CQV8-136	Q9CQV8	136	149	1433B_MOUSE	Mus musculus	246	S	Phosphorylation	PSP	NULL														
IPR000308-152	IPR000308	P61981-142	P61981	142	152	1433G_HUMAN	Homo sapiens	247	K	Acetylation	PSP	NULL														
IPR000308-152	IPR000308	P61981-142	P61981	142	152	1433G_HUMAN	Homo sapiens	247	K	Ubiquitination	PSP	NULL														
IPR000308-152	IPR000308	P61982-142	P61982	142	152	1433G_MOUSE	Mus musculus	247	K	Acetylation	PSP	NULL														
IPR000308-152	IPR000308	P61982-142	P61982	142	152	1433G_MOUSE	Mus musculus	247	K	Ubiquitination	PSP	NULL														
IPR000308-152	IPR000308	P68510-142	P68510	142	152	1433F_MOUSE	Mus musculus	246	K	Ubiquitination	PSP	NULL														
IPR000308-152	IPR000308	Q04917-142	Q04917	142	152	1433F_HUMAN	Homo sapiens	246	K	Acetylation	PSP	NULL														
IPR000308-152	IPR000308	Q04917-142	Q04917	142	152	1433F_HUMAN	Homo sapiens	246	K	Ubiquitination	PSP	NULL														
IPR000308-153	IPR000308	P31946-140	P31946	140	153	1433B_HUMAN	Homo sapiens	246	K	Ubiquitination	PSP	NULL														
IPR000308-153	IPR000308	P35213-140	P35213	140	153	1433B_RAT	Rattus norvegicus	246	K	Ubiquitination	PSP	NULL														
IPR000308-153	IPR000308	P63101-138	P63101	138	153	1433Z_MOUSE	Mus musculus	245	K	Acetylation	PSP	NULL														
IPR000308-153	IPR000308	P63101-138	P63101	138	153	1433Z_MOUSE	Mus musculus	245	K	Ubiquitination	PSP	NULL														
IPR000308-153	IPR000308	P63102-138	P63102	138	153	1433Z_RAT	Rattus norvegicus	245	K	Acetylation	PSP	NULL														
IPR000308-153	IPR000308	P63102-138	P63102	138	153	1433Z_RAT	Rattus norvegicus	245	K	Ubiquitination	PSP	NULL														
IPR000308-153	IPR000308	P63104-138	P63104	138	153	1433Z_HUMAN	Homo sapiens	245	K	Acetylation	PSP	NULL														
IPR000308-153	IPR000308	P63104-138	P63104	138	153	1433Z_HUMAN	Homo sapiens	245	K	Ubiquitination	PSP	NULL														
IPR000308-153	IPR000308	P68510-143	P68510	143	153	1433F_MOUSE	Mus musculus	246	K	Ubiquitination	PSP	NULL														
IPR000308-153	IPR000308	Q04917-143	Q04917	143	153	1433F_HUMAN	Homo sapiens	246	K	Ubiquitination	PSP	NULL														
IPR000308-153	IPR000308	Q9CQV8-140	Q9CQV8	140	153	1433B_MOUSE	Mus musculus	246	K	Ubiquitination	PSP	NULL														
IPR000308-154	IPR000308	P27348-139	P27348	139	154	1433T_HUMAN	Homo sapiens	245	K	Acetylation	PSP	NULL														
IPR000308-154	IPR000308	P27348-139	P27348	139	154	1433T_HUMAN	Homo sapiens	245	K	Ubiquitination	PSP	NULL														
IPR000308-154	IPR000308	P31947-141	P31947	141	154	1433S_HUMAN	Homo sapiens	248	K	Acetylation	PSP	NULL														
IPR000308-154	IPR000308	P62258-142	P62258	142	154	1433E_HUMAN	Homo sapiens	255	K	Acetylation	PSP	NULL														
IPR000308-154	IPR000308	P62258-142	P62258	142	154	1433E_HUMAN	Homo sapiens	255	K	Ubiquitination	PSP	NULL														
IPR000308-154	IPR000308	P62259-142	P62259	142	154	1433E_MOUSE	Mus musculus	255	K	Acetylation	PSP	NULL														
IPR000308-154	IPR000308	P62259-142	P62259	142	154	1433E_MOUSE	Mus musculus	255	K	Ubiquitination	PSP	NULL														
IPR000308-154	IPR000308	P62260-142	P62260	142	154	1433E_RAT	Rattus norvegicus	255	K	Acetylation	PSP	NULL														
IPR000308-154	IPR000308	P62260-142	P62260	142	154	1433E_RAT	Rattus norvegicus	255	K	Ubiquitination	PSP	NULL														
IPR000308-154	IPR000308	P63101-139	P63101	139	154	1433Z_MOUSE	Mus musculus	245	K	Ubiquitination	PSP	NULL														
IPR000308-154	IPR000308	P63102-139	P63102	139	154	1433Z_RAT	Rattus norvegicus	245	K	Ubiquitination	PSP	NULL														
IPR000308-154	IPR000308	P63104-139	P63104	139	154	1433Z_HUMAN	Homo sapiens	245	K	Acetylation	PSP	NULL														
IPR000308-154	IPR000308	P63104-139	P63104	139	154	1433Z_HUMAN	Homo sapiens	245	K	Ubiquitination	PSP	NULL														
IPR000308-154	IPR000308	P68254-139	P68254	139	154	1433T_MOUSE	Mus musculus	245	K	Ubiquitination	PSP	NULL														
IPR000308-155	IPR000308	Q04917-145	Q04917	145	155	1433F_HUMAN	Homo sapiens	246	S	Phosphorylation	PSP	NULL														
IPR000308-156	IPR000308	P35213-143	P35213	143	156	1433B_RAT	Rattus norvegicus	246	T	Phosphorylation	PSP	NULL	T	p	Ywhab	14-3-3 beta	Adaptor/scaffold	56011	3q42	rat	3136106	SGDNKQTtVSNSQQA	14-3-3	molecular association, regulation	RAF1(DISRUPTS); PKCZ(DISRUPTS)	10620507
IPR000308-156	IPR000308	P27348-141	P27348	141	156	1433T_HUMAN	Homo sapiens	245	T	Phosphorylation	PSP	NULL														
IPR000308-156	IPR000308	P31946-143	P31946	143	156	1433B_HUMAN	Homo sapiens	246	T	Phosphorylation	HPRD 9.0	10620507														
IPR000308-159	IPR000308	P31947-146	P31947	146	159	1433S_HUMAN	Homo sapiens	248	S	Phosphorylation	PSP	NULL														
IPR000308-160	IPR000308	P27348-145	P27348	145	160	1433T_HUMAN	Homo sapiens	245	S	Phosphorylation	PSP	NULL														
IPR000308-160	IPR000308	P62258-148	P62258	148	160	1433E_HUMAN	Homo sapiens	255	S	Phosphorylation	PSP	NULL														
IPR000308-160	IPR000308	P63104-145	P63104	145	160	1433Z_HUMAN	Homo sapiens	245	S	Phosphorylation	PSP	NULL														
IPR000308-162	IPR000308	P31947-149	P31947	149	162	1433S_HUMAN	Homo sapiens	248	S	Phosphorylation	PSP	NULL														
IPR000308-162	IPR000308	P61981-152	P61981	152	162	1433G_HUMAN	Homo sapiens	247	K	Acetylation	PSP	NULL														
IPR000308-162	IPR000308	P61981-152	P61981	152	162	1433G_HUMAN	Homo sapiens	247	K	Ubiquitination	PSP	NULL														
IPR000308-162	IPR000308	P61982-152	P61982	152	162	1433G_MOUSE	Mus musculus	247	K	Ubiquitination	PSP	NULL														
IPR000308-164	IPR000308	P27348-149	P27348	149	164	1433T_HUMAN	Homo sapiens	245	Y	Phosphorylation	PSP	NULL														
IPR000308-164	IPR000308	P31946-151	P31946	151	164	1433B_HUMAN	Homo sapiens	246	Y	Phosphorylation	PSP	NULL														
IPR000308-164	IPR000308	P31947-151	P31947	151	164	1433S_HUMAN	Homo sapiens	248	Y	Phosphorylation	PSP	NULL														
IPR000308-164	IPR000308	P63104-149	P63104	149	164	1433Z_HUMAN	Homo sapiens	245	Y	Phosphorylation	PSP	NULL														
IPR000308-164	IPR000308	Q04917-154	Q04917	154	164	1433F_HUMAN	Homo sapiens	246	Y	Phosphorylation	PSP	NULL														
IPR000308-165	IPR000308	P61981-155	P61981	155	165	1433G_HUMAN	Homo sapiens	247	S	Phosphorylation	PSP	NULL														
IPR000308-165	IPR000308	P62258-153	P62258	153	165	1433E_HUMAN	Homo sapiens	255	K	Methylation	PSP	NULL														
IPR000308-165	IPR000308	P62258-153	P62258	153	165	1433E_HUMAN	Homo sapiens	255	K	Ubiquitination	PSP	NULL														
IPR000308-165	IPR000308	P62259-153	P62259	153	165	1433E_MOUSE	Mus musculus	255	K	Acetylation	PSP	NULL														

IPR000308-171	IPR000308	P27348-156	P27348	156	171	1433T_HUMAN	Homo sapiens	245	S	Phosphorylation	PSP	NULL																
IPR000308-172	IPR000308	P27348-157	P27348	157	172	1433T_HUMAN	Homo sapiens	245	K	Acetylation	PSP	NULL																
IPR000308-172	IPR000308	P27348-157	P27348	157	172	1433T_HUMAN	Homo sapiens	245	K	Ubiquitination	PSP	NULL																
IPR000308-172	IPR000308	P31946-159	P31946	159	172	1433B_HUMAN	Homo sapiens	246	K	Acetylation	PSP	NULL																
IPR000308-172	IPR000308	P31946-159	P31946	159	172	1433B_HUMAN	Homo sapiens	246	K	Ubiquitination	PSP	NULL																
IPR000308-172	IPR000308	P31947-159	P31947	159	172	1433S_HUMAN	Homo sapiens	248	K	Acetylation	PSP	NULL																
IPR000308-172	IPR000308	P35213-159	P35213	159	172	1433B_RAT	Rattus norvegicus	246	K	Acetylation	PSP	NULL																
IPR000308-172	IPR000308	P42644-162	P42644	162	172	14333_ARATH	Arabidopsis thaliana	255	T	Phosphorylation	Uniport_201509	22092075																
IPR000308-172	IPR000308	P61981-162	P61981	162	172	1433G_HUMAN	Homo sapiens	247	K	Acetylation	PSP	NULL																
IPR000308-172	IPR000308	P61981-162	P61981	162	172	1433G_HUMAN	Homo sapiens	247	K	Ubiquitination	PSP	NULL																
IPR000308-172	IPR000308	P61982-162	P61982	162	172	1433G_MOUSE	Mus musculus	247	K	Acetylation	PSP	NULL																
IPR000308-172	IPR000308	P61982-162	P61982	162	172	1433G_MOUSE	Mus musculus	247	K	Ubiquitination	PSP	NULL																
IPR000308-172	IPR000308	P63101-157	P63101	157	172	1433Z_MOUSE	Mus musculus	245	K	Acetylation	PSP	NULL																
IPR000308-172	IPR000308	P63101-157	P63101	157	172	1433Z_MOUSE	Mus musculus	245	K	Ubiquitination	PSP	NULL																
IPR000308-172	IPR000308	P63102-157	P63102	157	172	1433Z_RAT	Rattus norvegicus	245	K	Acetylation	PSP	NULL																
IPR000308-172	IPR000308	P63104-157	P63104	157	172	1433Z_HUMAN	Homo sapiens	245	K	Acetylation	PSP	NULL																
IPR000308-172	IPR000308	P63104-157	P63104	157	172	1433Z_HUMAN	Homo sapiens	245	K	Ubiquitination	PSP	NULL																
IPR000308-172	IPR000308	P68254-157	P68254	157	172	1433T_MOUSE	Mus musculus	245	K	Ubiquitination	PSP	NULL																
IPR000308-172	IPR000308	Q9CQV8-159	Q9CQV8	159	172	1433B_MOUSE	Mus musculus	246	K	Ubiquitination	PSP	NULL																
IPR000308-173	IPR000308	P27348-158	P27348	158	173	1433T_HUMAN	Homo sapiens	245	K	Acetylation	PSP	NULL																
IPR000308-173	IPR000308	P27348-158	P27348	158	173	1433T_HUMAN	Homo sapiens	245	K	Ubiquitination	PSP	NULL																
IPR000308-173	IPR000308	P31946-160	P31946	160	173	1433B_HUMAN	Homo sapiens	246	K	Ubiquitination	PSP	NULL																
IPR000308-173	IPR000308	P63101-158	P63101	158	173	1433Z_MOUSE	Mus musculus	245	K	Ubiquitination	PSP	NULL																
IPR000308-173	IPR000308	P63104-158	P63104	158	173	1433Z_HUMAN	Homo sapiens	245	K	Ubiquitination	PSP	NULL																
IPR000308-173	IPR000308	P68254-158	P68254	158	173	1433T_MOUSE	Mus musculus	245	K	Ubiquitination	PSP	NULL																
IPR000308-173	IPR000308	Q9CQV8-160	Q9CQV8	160	173	1433B_MOUSE	Mus musculus	246	K	Ubiquitination	PSP	NULL																
IPR000308-178	IPR000308	P61981-168	P61981	168	178	1433G_HUMAN	Homo sapiens	247	T	Phosphorylation	PSP	NULL																
IPR000308-178	IPR000308	Q04917-168	Q04917	168	178	1433F_HUMAN	Homo sapiens	246	T	Phosphorylation	PSP	NULL																
IPR000308-189	IPR000308	P61981-179	P61981	179	189	1433G_HUMAN	Homo sapiens	247	Y	Phosphorylation	PSP	NULL																
IPR000308-190	IPR000308	P61981-180	P61981	180	190	1433G_HUMAN	Homo sapiens	247	S	Phosphorylation	PSP	NULL																
IPR000308-193	IPR000308	P31946-180	P31946	180	193	1433B_HUMAN	Homo sapiens	246	Y	Phosphorylation	PSP	NULL																
IPR000308-193	IPR000308	P62258-181	P62258	181	193	1433E_HUMAN	Homo sapiens	255	Y	Phosphorylation	PSP	NULL																
IPR000308-193	IPR000308	P63104-178	P63104	178	193	1433Z_HUMAN	Homo sapiens	245	Y	Phosphorylation	PSP	NULL																
IPR000308-194	IPR000308	P63101-179	P63101	179	194	1433Z_MOUSE	Mus musculus	245	Y	Phosphorylation	PSP	NULL	Y	p	Ywhaz	14-3-3 zeta	Adaptor/scaffold; Motility/polarity/chemotaxis	22631	15|15 B3.1	mouse	7316700	LNFSVFYyEILNSPE	14-3-3	molecular association, regulation; phosphorylation		SHC1(INDUCES)	19218246	important for the phosphorylation of Akt and cytokine-mediated cell survival
IPR000308-199	IPR000308	O70456-186	O70456	186	199	1433S_MOUSE	Mus musculus	248	S	Phosphorylation	PSP	NULL	S	p	Sfn	14-3-3 sigma	Adaptor/scaffold	55948	4 D2.3|4 66.25 cM	mouse	456054	FHYEIANsPEEAISL	14-3-3	molecular association, regulation		Abl(DISRUPTS)	19220809	
IPR000308-199	IPR000308	P31946-186	P31946	186	199	1433B_HUMAN	Homo sapiens	246	S	Phosphorylation	PSP	NULL	S	p	YWHAB	14-3-3 beta	Adaptor/scaffold	7529	20q13.12	human	449845	FYYEILNsPEKACSL	14-3-3	molecular association, regulation		Abl iso2(DISRUPTS)	15696159	
IPR000308-199	IPR000308	P31947-186	P31947	186	199	1433S_HUMAN	Homo sapiens	248	S	Phosphorylation	PSP	NULL	S	p	SFN	14-3-3 sigma	Adaptor/scaffold	2810	1p36.11	human	456054	FHYEIANsPEEAISL	14-3-3	molecular association, regulation; phosphorylation	apoptosis, induced	CBY1(DISRUPTS); JNK1(INDUCES); BAX(DISRUPTS)	15071501; 16009721; 26147002	
IPR000308-199	IPR000308	P63104-184	P63104	184	199	1433Z_HUMAN	Homo sapiens	245	S	Phosphorylation	PSP	NULL	S	p	YWHAZ	14-3-3 zeta	Adaptor/scaffold; Motility/polarity/chemotaxis	7534	8q22.3	human	449855	FYYEILNsPEKACSL	14-3-3	protein stabilization; molecular association, regulation; protein conformation; phosphorylation		HSP20(INDUCES); JNK1(NOT_REPORTED); BAX(DISRUPTS)	15071501; 16009721; 18559254; 21081103	
IPR000308-199	IPR000308	P29361-184	P29361	184	199	1433Z_SHEEP	Ovis aries	245	S	Phosphorylation	PSP	NULL																
IPR000308-199	IPR000308	P35213-186	P35213	186	199	1433B_RAT	Rattus norvegicus	246	S	Phosphorylation	PSP	NULL																
IPR000308-199	IPR000308	P68250-186	P68250	186	199	1433B_BOVIN	Bos taurus	246	S	Phosphorylation	PhosphoELM.10011	7890696																
IPR000308-199	IPR000308	P68251-149	P68251	149	199	1433B_SHEEP	Ovis aries	193	S	Phosphorylation	PSP	NULL																
IPR000308-204	IPR000308	P61982-194	P61982	194	204	1433G_MOUSE	Mus musculus	247	C	S-Nitrosylation	dbSNOv2	21278135																
IPR000308-204	IPR000308	P62260-192	P62260	192	204	1433E_RAT	Rattus norvegicus	255	C	S-Nitrosylation	dbSNOv2	17629318																
IPR000308-204	IPR000308	P63104-189	P63104	189	204	1433Z_HUMAN	Homo sapiens	245	C	S-Nitrosylation	dbSNOv2	19483679																
IPR000308-208	IPR000308	P27348-193	P27348	193	208	1433T_HUMAN	Homo sapiens	245	K	Ubiquitination	PSP	NULL																
IPR000308-208	IPR000308	P31946-195	P31946	195	208	1433B_HUMAN	Homo sapiens	246	K	Ubiquitination	PSP	NULL																
IPR000308-208	IPR000308	P61981-198	P61981	198	208	1433G_HUMAN	Homo sapiens	247	K	Ubiquitination	PSP	NULL																
IPR000308-208	IPR000308	P62258-196	P62258	196	208	1433E_HUMAN	Homo sapiens	255	K	Ubiquitination	PSP	NULL																
IPR000308-208	IPR000308	P62259-196	P62259	196	208	1433E_MOUSE	Mus musculus	255	K	Acetylation	PSP	NULL																
IPR000308-208	IPR000308	P62259-196	P62259	196	208	1433E_MOUSE	Mus musculus	255	K	Ubiquitination	PSP	NULL																
IPR000308-208	IPR000308	P63101-193	P63101	193	208	1433Z_MOUSE	Mus musculus	245	K	Ubiquitination	PSP	NULL																
IPR000308-208	IPR000308	P63104-193	P63104	193	208	1433Z_HUMAN	Homo sapiens	245	K	Ubiquitination	PSP	NULL																
IPR000308-208	IPR000308	P68254-193	P68254	193	208	1433T_MOUSE	Mus musculus	245	K	Ubiquitination	PSP	NULL																
IPR000308-208	IPR000308	Q9CQV8-195	Q9CQV8	195	208	1433B_MOUSE	Mus musculus	246	K	Ubiquitination	PSP	NULL																
IPR000308-209	IPR000308	P61981-199	P61981	199	209	1433G_HUMAN	Homo sapiens	247	T	Phosphorylation	PSP	NULL																
IPR000308-209	IPR000308	P63104-194	P63104	194	209	1433Z_HUMAN	Homo sapiens	245	T	Phosphorylation	PSP	NULL																
IPR000308-220	IPR000308	P27348-205	P27348	205	220	1433T_HUMAN	Homo sapiens	245	T	Phosphorylation	PSP	NULL																
IPR000308-220	IPR000308	P31946-207	P31946	207	220	1433B_HUMAN	Homo sapiens	246	T	Phosphorylation	PSP	NULL																
IPR000308-220	IPR000308	P31947-207	P31947	207	220	1433S_HUMAN	Homo sapiens	248	T	Phosphorylation	PSP	NULL																
IPR000308-220	IPR000308	P61981-210	P61981	210	220	1433G_HUMAN	Homo sapiens	247	T	Phosphorylation	PSP	NULL																
IPR000308-220	IPR000308	P62258-208	P62258	208	220	1433E_HUMAN	Homo sapiens	255	T	Phosphorylation	PSP	NULL																
IPR000308-220	IPR000308	P63104-205	P63104	205	220	1433Z_HUMAN	Homo sapiens	245	T	Phosphorylation	PSP	NULL																
IPR000308-222	IPR000308	P31947-209	P31947	209	222	1433S_HUMAN	Homo sapiens	248	S	Phosphorylation	PSP	NULL																
IPR000308-222	IPR000308	P34730-212	P34730	212	222	BMH2_YEAST	Saccharomyces cerevisiae	273	S	Phosphorylation	SysPTM 1.1	18407956																
IPR000308-222	IPR000308	P62258-210	P62258	210	222	1433E_HUMAN	Homo sapiens	255	S	Phosphorylation	PSP	NULL																
IPR000308-222	IPR000308	P62259-210	P62259	210	222	1433E_MOUSE	Mus musculus	255	S	Phosphorylation	PSP	NULL																
IPR000308-222	IPR000308	P62260-210	P62260	210	222	1433E_RAT	Rattus norvegicus	255	S	Phosphorylation	PSP	NULL																
IPR000308-222	IPR000308	P63101-207	P63101	207	222	1433Z_MOUSE	Mus musculus	245	S	Phosphorylation	PSP	NULL																
IPR000308-222	IPR000308	P63102-207	P63102	207	222	1433Z_RAT	Rattus norvegicus	245	S	Phosphorylation	PSP	NULL																
IPR000308-222	IPR000308	P63104-207	P63104	207	222	1433Z_HUMAN	Homo sapiens	245	S	Phosphorylation	PSP	NULL																
IPR000308-225	IPR000308	P27348-210	P27348	210	225	1433T_HUMAN	Homo sapiens	245	S	Phosphorylation	PSP	NULL																
IPR000308-225	IPR000308	P31946-212	P31946	212	225	1433B_HUMAN	Homo sapiens	246	S	Phosphorylation	PSP	NULL																
IPR000308-225	IPR000308	P61981-215	P61981	215	225	1433G_HUMAN	Homo sapiens	247	S	Phosphorylation	PSP	NULL																

IPR000308-225	IPR000308	P62258-213	P62258	213	225	1433E_HUMAN	Homo sapiens	255	S	Phosphorylation	PSP	NULL												
IPR000308-225	IPR000308	P62259-213	P62259	213	225	1433E_MOUSE	Mus musculus	255	S	Phosphorylation	SysPTM 1.1	17203969												
IPR000308-225	IPR000308	P62260-213	P62260	213	225	1433E_RAT	Rattus norvegicus	255	S	Phosphorylation	PSP	NULL												
IPR000308-225	IPR000308	P63102-210	P63102	210	225	1433Z_RAT	Rattus norvegicus	245	S	Phosphorylation	PSP	NULL												
IPR000308-225	IPR000308	P63104-210	P63104	210	225	1433Z_HUMAN	Homo sapiens	245	S	Phosphorylation	PSP	NULL												
IPR000308-225	IPR000308	Q04917-215	Q04917	215	225	1433F_HUMAN	Homo sapiens	246	S	Phosphorylation	HPRD 9.0	20068231												
IPR000308-226	IPR000308	P27348-211	P27348	211	226	1433T_HUMAN	Homo sapiens	245	Y	Phosphorylation	PSP	NULL												
IPR000308-226	IPR000308	P31946-213	P31946	213	226	1433B_HUMAN	Homo sapiens	246	Y	Phosphorylation	PSP	NULL												
IPR000308-226	IPR000308	P31947-213	P31947	213	226	1433S_HUMAN	Homo sapiens	248	Y	Phosphorylation	HPRD 9.0	20068231												
IPR000308-226	IPR000308	P61981-216	P61981	216	226	1433G_HUMAN	Homo sapiens	247	Y	Phosphorylation	PSP	NULL												
IPR000308-226	IPR000308	P62258-214	P62258	214	226	1433E_HUMAN	Homo sapiens	255	Y	Phosphorylation	PSP	NULL												
IPR000308-226	IPR000308	P62259-214	P62259	214	226	1433E_MOUSE	Mus musculus	255	Y	Phosphorylation	PSP	NULL												
IPR000308-226	IPR000308	P63101-211	P63101	211	226	1433Z_MOUSE	Mus musculus	245	Y	Phosphorylation	PSP	NULL												
IPR000308-226	IPR000308	P63104-211	P63104	211	226	1433Z_HUMAN	Homo sapiens	245	Y	Phosphorylation	PSP	NULL												
IPR000308-226	IPR000308	Q04917-216	Q04917	216	226	1433F_HUMAN	Homo sapiens	246	Y	Phosphorylation	PSP	NULL												
IPR000308-227	IPR000308	P27348-212	P27348	212	227	1433T_HUMAN	Homo sapiens	245	K	Methylation	PSP	NULL												
IPR000308-227	IPR000308	P63104-212	P63104	212	227	1433Z_HUMAN	Homo sapiens	245	K	Methylation	PSP	NULL												
IPR000308-229	IPR000308	O70456-216	O70456	216	229	1433S_MOUSE	Mus musculus	248	S	Phosphorylation	PSP	NULL												
IPR000308-229	IPR000308	P27348-214	P27348	214	229	1433T_HUMAN	Homo sapiens	245	S	Phosphorylation	PSP	NULL												
IPR000308-229	IPR000308	P31946-216	P31946	216	229	1433B_HUMAN	Homo sapiens	246	S	Phosphorylation	PSP	NULL												
IPR000308-229	IPR000308	P31947-216	P31947	216	229	1433S_HUMAN	Homo sapiens	248	S	Phosphorylation	PSP	NULL												
IPR000308-229	IPR000308	P61981-219	P61981	219	229	1433G_HUMAN	Homo sapiens	247	S	Phosphorylation	PSP	NULL												
IPR000308-229	IPR000308	P62258-217	P62258	217	229	1433E_HUMAN	Homo sapiens	255	S	Phosphorylation	PSP	NULL												
IPR000308-229	IPR000308	P63104-214	P63104	214	229	1433Z_HUMAN	Homo sapiens	245	S	Phosphorylation	PSP	NULL												
IPR000308-229	IPR000308	Q04917-219	Q04917	219	229	1433F_HUMAN	Homo sapiens	246	S	Phosphorylation	PSP	NULL												
IPR000308-230	IPR000308	P27348-215	P27348	215	230	1433T_HUMAN	Homo sapiens	245	T	Phosphorylation	PSP	NULL												
IPR000308-230	IPR000308	P31946-217	P31946	217	230	1433B_HUMAN	Homo sapiens	246	T	Phosphorylation	PSP	NULL												
IPR000308-230	IPR000308	P31947-217	P31947	217	230	1433S_HUMAN	Homo sapiens	248	T	Phosphorylation	PSP	NULL												
IPR000308-230	IPR000308	P61981-220	P61981	220	230	1433G_HUMAN	Homo sapiens	247	T	Phosphorylation	PSP	NULL												
IPR000308-230	IPR000308	P62258-218	P62258	218	230	1433E_HUMAN	Homo sapiens	255	T	Phosphorylation	PSP	NULL												
IPR000308-230	IPR000308	P63104-215	P63104	215	230	1433Z_HUMAN	Homo sapiens	245	T	Phosphorylation	PSP	NULL												
IPR000308-230	IPR000308	Q04917-220	Q04917	220	230	1433F_HUMAN	Homo sapiens	246	T	Phosphorylation	PSP	NULL												
IPR000308-241	IPR000308	P31946-228	P31946	228	241	1433B_HUMAN	Homo sapiens	246	T	Phosphorylation	PSP	NULL												
IPR000308-241	IPR000308	P63101-226	P63101	226	241	1433Z_MOUSE	Mus musculus	245	T	Phosphorylation	PSP	NULL												
IPR000308-244	IPR000308	P27348-229	P27348	229	244	1433T_HUMAN	Homo sapiens	245	T	Phosphorylation	PSP	NULL												
IPR000308-244	IPR000308	P31946-231	P31946	231	244	1433B_HUMAN	Homo sapiens	246	T	Phosphorylation	PSP	NULL												
IPR000308-244	IPR000308	P61981-234	P61981	234	244	1433G_HUMAN	Homo sapiens	247	T	Phosphorylation	PSP	NULL												
IPR000308-244	IPR000308	P62258-232	P62258	232	244	1433E_HUMAN	Homo sapiens	255	T	Phosphorylation	PSP	NULL												
IPR000308-244	IPR000308	P63101-229	P63101	229	244	1433Z_MOUSE	Mus musculus	245	T	Phosphorylation	PSP	NULL												
IPR000308-244	IPR000308	P63104-229	P63104	229	244	1433Z_HUMAN	Homo sapiens	245	T	Phosphorylation	PSP	NULL												
IPR000308-244	IPR000308	P68254-229	P68254	229	244	1433T_MOUSE	Mus musculus	245	T	Phosphorylation	PSP	NULL												
IPR000308-245	IPR000308	P27348-230	P27348	230	245	1433T_HUMAN	Homo sapiens	245	S	Phosphorylation	PSP	NULL												
IPR000308-245	IPR000308	P31946-232	P31946	232	245	1433B_HUMAN	Homo sapiens	246	S	Phosphorylation	PSP	NULL												
IPR000308-245	IPR000308	P42657-234	P42657	234	245	RAD25_SCHPO	Schizosaccharomyces pombe	270	S	Phosphorylation	SysPTM 1.1	18257517												
IPR000308-245	IPR000308	P48347-234	P48347	234	245	14310_ARATH	Arabidopsis thaliana	254	S	Phosphorylation	SysPTM 1.1	18463617												
IPR000308-245	IPR000308	P61981-235	P61981	235	245	1433G_HUMAN	Homo sapiens	247	S	Phosphorylation	PSP	NULL												
IPR000308-245	IPR000308	P61982-235	P61982	235	245	1433G_MOUSE	Mus musculus	247	S	Phosphorylation	PSP	NULL												
IPR000308-245	IPR000308	P62258-233	P62258	233	245	1433E_HUMAN	Homo sapiens	255	S	Phosphorylation	PSP	NULL												
IPR000308-245	IPR000308	P62259-233	P62259	233	245	1433E_MOUSE	Mus musculus	255	S	Phosphorylation	PSP	NULL												
IPR000308-245	IPR000308	P63101-230	P63101	230	245	1433Z_MOUSE	Mus musculus	245	S	Phosphorylation	PSP	NULL												
IPR000308-245	IPR000308	P63104-230	P63104	230	245	1433Z_HUMAN	Homo sapiens	245	S	Phosphorylation	PSP	NULL												
IPR000308-245	IPR000308	P68254-230	P68254	230	245	1433T_MOUSE	Mus musculus	245	S	Phosphorylation	PSP	NULL												
IPR000308-247	IPR000308	P63104-232	P63104	232	247	1433Z_HUMAN	Homo sapiens	245	T	Phosphorylation	PSP	NULL	T	p	YWHAZ	14-3-3 zeta	Adaptor/scaffold; Motility/polarity/chemotaxis	7534	8q22.3	human	449846	LTLWTSDtQGDEAEA	molecular association, regulation; protein conformation; activity, inhibited	14-3-3 zeta(DISRUPTS); RAF1(DISRUPTS); Tau iso3(DISRUPTS); HSP20(DISRUPTS)	14613942; 15119949; 18559254; 21081103; 9360956
IPR000308-247	IPR000308	P27348-232	P27348	232	247	1433T_HUMAN	Homo sapiens	245	S	Phosphorylation	PSP	NULL												
IPR000308-247	IPR000308	P29361-232	P29361	232	247	1433Z_SHEEP	Ovis aries	245	T	Phosphorylation	PhosphoELM.10011	9360956												
IPR000308-247	IPR000308	P63101-232	P63101	232	247	1433Z_MOUSE	Mus musculus	245	T	Phosphorylation	PSP	NULL												
IPR000308-247	IPR000308	P68254-232	P68254	232	247	1433T_MOUSE	Mus musculus	245	S	Phosphorylation	PSP	NULL												
IPR000308-248	IPR000308	P42644-238	P42644	238	248	14333_ARATH	Arabidopsis thaliana	255	T	Phosphorylation	SysPTM 1.1	18463617												
IPR000308-248	IPR000308	Q96299-239	Q96299	239	248	14339_ARATH	Arabidopsis thaliana	263	S	Phosphorylation	Uniport_201509	19245862												
IPR000308-251	IPR000308	P46077-248	P46077	248	251	14334_ARATH	Arabidopsis thaliana	267	S	Phosphorylation	SysPTM 1.1	18463617												
IPR000308-252	IPR000308	P42657-241	P42657	241	252	RAD25_SCHPO	Schizosaccharomyces pombe	270	S	Phosphorylation	SysPTM 1.1	18257517												
IPR000308-256	IPR000308	P62258-244	P62258	244	256	1433E_HUMAN	Homo sapiens	255	K	Ubiquitination	PSP	NULL												
IPR000308-256	IPR000308	P62259-244	P62259	244	256	1433E_MOUSE	Mus musculus	255	K	Ubiquitination	PSP	NULL												
IPR000308-279	IPR000308	P42657-253	P42657	253	279	RAD25_SCHPO	Schizosaccharomyces pombe	270	S	Phosphorylation	SysPTM 1.1	18257517												
IPR000308-282	IPR000308	P42645-267	P42645	267	282	14335_ARATH	Arabidopsis thaliana	268	S	Phosphorylation	Uniport_201509	19376835												
IPR000308-283	IPR000308	P31947-248	P31947	248	283	1433S_HUMAN	Homo sapiens	248	S	Phosphorylation	PSP	NULL												
IPR000308-283	IPR000308	P42643-267	P42643	267	283	14331_ARATH	Arabidopsis thaliana	267	S	Phosphorylation	Uniport_201509	19376835												
IPR000308-296	IPR000308	P92177-262	P92177	262	296	1433E_DROME	Drosophila melanogaster	262	S	Phosphorylation	SysPTM 1.1	18327897												
