# Supplementary material for: The dynamic and stress-adaptive signaling hub of 14-3-3: emerging mechanisms of regulation and context-dependent protein–protein interactions
Source: Oncogene. 2018 Jun 18;37(42):5587–604. doi: 10.1038/s41388-018-0348-3 (PMC6193947; doi:10.1038/s41388-018-0348-3)
Supplement: Supplementary file 4 — Supplemental Table S1. Tab4 [file 41388_2018_348_MOESM4_ESM.pdf]

MAP	SAPH-ire FPx Score (rel to Family max)	P63104 Native Position	P63104 Resi	Family	Functional UID-NP	Functional UID	Functional NP	AP	Entry name	OID	Length	Resi of Functional UID-NP	Native PTM	Database	Reference	PROTEIN_PSPfxn	SITE_+/-7_AA_PSPfxn	DOMAIN_PSPfxn	ON_FUNCTION_PSPfxn	ON_PROCESS_PSPfxn	ON_PROT_INTERACT_PSPfxn	PMIDs_PSPfxn	NOTES_PSPfxn
IPR000308-59	82.71092193	P63104-49	K	IPR000308	P61982-50	P61982	50	59	1433G_MOUSE	Mus musculus	247	K	Acetylation	PSP	NULL	14-3-3 gamma	NLLSVAYkNVVGARR	14-3-3	molecular association, regulation		14-3-3 gamma(INDUCES); STAR(INDUCES)	25086053	
IPR000308-59	82.71092193	P63104-49	K	IPR000308	P61982-50	P61982	50	59	1433G_MOUSE	Mus musculus	247	K	Ubiquitination	PSP	NULL	14-3-3 gamma	NLLSVAYkNVVGARR	14-3-3	molecular association, regulation		14-3-3 gamma(INDUCES); STAR(INDUCES)	25086053	
IPR000308-59	82.71092193	P63104-49	K	IPR000308	P62258-50	P62258	50	59	1433E_HUMAN	Homo sapiens	255	K	Acetylation	PSP	NULL	14-3-3 epsilon	NLLSVAYkNVIGARR	14-3-3	molecular association, regulation		SFRS6(DISRUPTS); PLEKHQ1(DISRUPTS); SFRS4(DISRUPTS); DOK3(DISRUPTS); TSC2(DISRUPTS); ARAF(DISRUPTS); LARP(DISRUPTS); SRm300(DISRUPTS); SF2(DISRUPTS); TSC22D1(DISRUPTS); RAF1(DISRUPTS); GAB2(DISRUPTS); VASP(DISRUPTS); HDAC4(DISRUPTS); TRA2B(DISRUPTS); GBF1(DISRUPTS)	19608861	
IPR000308-59	82.71092193	P63104-49	K	IPR000308	P62258-50	P62258	50	59	1433E_HUMAN	Homo sapiens	255	K	Ubiquitination	PSP	NULL	14-3-3 epsilon	NLLSVAYkNVIGARR	14-3-3	molecular association, regulation		SFRS6(DISRUPTS); PLEKHQ1(DISRUPTS); SFRS4(DISRUPTS); DOK3(DISRUPTS); TSC2(DISRUPTS); ARAF(DISRUPTS); LARP(DISRUPTS); SRm300(DISRUPTS); SF2(DISRUPTS); TSC22D1(DISRUPTS); RAF1(DISRUPTS); GAB2(DISRUPTS); VASP(DISRUPTS); HDAC4(DISRUPTS); TRA2B(DISRUPTS); GBF1(DISRUPTS)	19608861	
IPR000308-135	68.6655591	P63104-120	K	IPR000308	P62258-123	P62258	123	135	1433E_HUMAN	Homo sapiens	255	K	Acetylation	PSP	NULL	14-3-3 epsilon	ESKVFYYkMKGDYHR	14-3-3	molecular association, regulation		SFRS6(DISRUPTS); PLEKHQ1(DISRUPTS); SFRS4(DISRUPTS); DOK3(DISRUPTS); TSC2(DISRUPTS); ARAF(DISRUPTS); LARP(DISRUPTS); SRm300(DISRUPTS); SF2(DISRUPTS); TSC22D1(DISRUPTS); RAF1(DISRUPTS); GAB2(DISRUPTS); VASP(DISRUPTS); HDAC4(DISRUPTS); TRA2B(DISRUPTS); GBF1(DISRUPTS)	19608861	
IPR000308-135	68.6655591	P63104-120	K	IPR000308	P62258-123	P62258	123	135	1433E_HUMAN	Homo sapiens	255	K	Ubiquitination	PSP	NULL	14-3-3 epsilon	ESKVFYYkMKGDYHR	14-3-3	molecular association, regulation		SFRS6(DISRUPTS); PLEKHQ1(DISRUPTS); SFRS4(DISRUPTS); DOK3(DISRUPTS); TSC2(DISRUPTS); ARAF(DISRUPTS); LARP(DISRUPTS); SRm300(DISRUPTS); SF2(DISRUPTS); TSC22D1(DISRUPTS); RAF1(DISRUPTS); GAB2(DISRUPTS); VASP(DISRUPTS); HDAC4(DISRUPTS); TRA2B(DISRUPTS); GBF1(DISRUPTS)	19608861	
IPR000308-130	51.57366248	P63104-115	K	IPR000308	P62258-118	P62258	118	130	1433E_HUMAN	Homo sapiens	255	K	Acetylation	PSP	NULL	14-3-3 epsilon	AANTGESkVFYYKMK	14-3-3	molecular association, regulation		SFRS6(DISRUPTS); PLEKHQ1(DISRUPTS); SFRS4(DISRUPTS); DOK3(DISRUPTS); TSC2(DISRUPTS); ARAF(DISRUPTS); LARP(DISRUPTS); SRm300(DISRUPTS); SF2(DISRUPTS); TSC22D1(DISRUPTS); RAF1(DISRUPTS); GAB2(DISRUPTS); VASP(DISRUPTS); HDAC4(DISRUPTS); TRA2B(DISRUPTS); GBF1(DISRUPTS)	19608861	
IPR000308-130	51.57366248	P63104-115	K	IPR000308	P62258-118	P62258	118	130	1433E_HUMAN	Homo sapiens	255	K	Ubiquitination	PSP	NULL	14-3-3 epsilon	AANTGESkVFYYKMK	14-3-3	molecular association, regulation		SFRS6(DISRUPTS); PLEKHQ1(DISRUPTS); SFRS4(DISRUPTS); DOK3(DISRUPTS); TSC2(DISRUPTS); ARAF(DISRUPTS); LARP(DISRUPTS); SRm300(DISRUPTS); SF2(DISRUPTS); TSC22D1(DISRUPTS); RAF1(DISRUPTS); GAB2(DISRUPTS); VASP(DISRUPTS); HDAC4(DISRUPTS); TRA2B(DISRUPTS); GBF1(DISRUPTS)	19608861	
IPR000308-68	51.14959927	P63104-58	S	IPR000308	P35213-60	P35213	60	68	1433B_RAT	Rattus norvegicus	246	S	Phosphorylation	PSP	NULL	14-3-3 beta	VVGARRSsWRVISSI	14-3-3	intracellular localization	transcription, altered		12619878	Double mutant inhibited a MEF2 reporter and promotes nuclear localization of HDAC4/GFP.
IPR000308-68	51.14959927	P63104-58	S	IPR000308	P61981-59	P61981	59	68	1433G_HUMAN	Homo sapiens	247	S	Phosphorylation	PSP	NULL	14-3-3 gamma	VVGARRSsWRVISSI	14-3-3	molecular association, regulation; intracellular localization		TNRC6A(INDUCES)	21118956	localizes to P-bodies
IPR000308-68	51.14959927	P63104-58	S	IPR000308	P61982-59	P61982	59	68	1433G_MOUSE	Mus musculus	247	S	Phosphorylation	PSP	NULL	14-3-3 gamma	VVGARRSsWRVISSI	14-3-3	molecular association, regulation		14-3-3 gamma(INDUCES); STAR(INDUCES)	25086053	
IPR000308-68	51.14959927	P63104-58	S	IPR000308	P63101-58	P63101	58	68	1433Z_MOUSE	Mus musculus	245	S	Phosphorylation	PSP	NULL	14-3-3 zeta	VVGARRSsWRVVSSI	14-3-3	molecular association, regulation	apoptosis, induced	14-3-3 zeta(DISRUPTS)	12865427; 20813501	
IPR000308-68	51.14959927	P63104-58	S	IPR000308	P63104-58	P63104	58	68	1433Z_HUMAN	Homo sapiens	245	S	Phosphorylation	PSP	NULL	14-3-3 zeta	VVGARRSsWRVVSSI	14-3-3	protein degradation; molecular association, regulation	apoptosis, altered	14-3-3 zeta(DISRUPTS); 14-3-3 zeta(INDUCES); Tau iso3(DISRUPTS); ASK1(DISRUPTS); 14-3-3 epsilon(DISRUPTS); HSP20(DISRUPTS); p53(DISRUPTS)	11956222; 16376338; 18559254; 19451227; 21081103	
IPR000308-73	50.43670447	P63104-63	S	IPR000308	P35213-65	P35213	65	73	1433B_RAT	Rattus norvegicus	246	S	Phosphorylation	PSP	NULL	14-3-3 beta	RSSWRVIsSIEQKTE	14-3-3	intracellular localization	transcription, altered		12619878	Double mutant inhibited a MEF2 reporter and promotes nuclear localization of HDAC4/GFP.
IPR000308-199	36.85595479	P63104-184	S	IPR000308	O70456-186	O70456	186	199	1433S_MOUSE	Mus musculus	248	S	Phosphorylation	PSP	NULL	14-3-3 sigma	FHYEIANsPEEAISL	14-3-3	molecular association, regulation		Abl(DISRUPTS)	19220809	
IPR000308-199	36.85595479	P63104-184	S	IPR000308	P31946-186	P31946	186	199	1433B_HUMAN	Homo sapiens	246	S	Phosphorylation	PSP	NULL	14-3-3 beta	FYYEILNsPEKACSL	14-3-3	molecular association, regulation		Abl iso2(DISRUPTS)	15696159	
IPR000308-199	36.85595479	P63104-184	S	IPR000308	P31947-186	P31947	186	199	1433S_HUMAN	Homo sapiens	248	S	Phosphorylation	PSP	NULL	14-3-3 sigma	FHYEIANsPEEAISL	14-3-3	molecular association, regulation; phosphorylation	apoptosis, induced	CBY1(DISRUPTS); JNK1(INDUCES); BAX(DISRUPTS)	15071501; 16009721; 26147002	
IPR000308-199	36.85595479	P63104-184	S	IPR000308	P63104-184	P63104	184	199	1433Z_HUMAN	Homo sapiens	245	S	Phosphorylation	PSP	NULL	14-3-3 zeta	FYYEILNsPEKACSL	14-3-3	protein stabilization; molecular association, regulation; protein conformation; phosphorylation		HSP20(INDUCES); JNK1(NOT_REPORTED); BAX(DISRUPTS)	15071501; 16009721; 18559254; 21081103	
IPR000308-247	14.61626821	P63104-232	T	IPR000308	P63104-232	P63104	232	247	1433Z_HUMAN	Homo sapiens	245	T	Phosphorylation	PSP	NULL	14-3-3 zeta	LTLWTSDtQGDEAEA		molecular association, regulation; protein conformation; activity, inhibited		14-3-3 zeta(DISRUPTS); RAF1(DISRUPTS); Tau iso3(DISRUPTS); HSP20(DISRUPTS)	14613942; 15119949; 18559254; 21081103; 9360956	
IPR000308-85	4.721782642		A	IPR000308	P31947-74	P31947	74	85	1433S_HUMAN	Homo sapiens	248	S	Phosphorylation	PSP	NULL	14-3-3 sigma	QKSNEEGsEEKGPEV	14-3-3		carcinogenesis, induced; transcription, induced		23741479	
IPR000308-79	2.638549157		T	IPR000308	P31947-69	P31947	69	79	1433S_HUMAN	Homo sapiens	248	S	Phosphorylation	PSP	NULL	14-3-3 sigma	LSSIEQKsNEEGSEE	14-3-3		carcinogenesis, induced; transcription, induced		23741479	
IPR000308-156	1.780563559		I	IPR000308	P35213-143	P35213	143	156	1433B_RAT	Rattus norvegicus	246	T	Phosphorylation	PSP	NULL	14-3-3 beta	SGDNKQTtVSNSQQA	14-3-3	molecular association, regulation		RAF1(DISRUPTS); PKCZ(DISRUPTS)	10620507	
IPR000308-145	1.697803042		A	IPR000308	P35213-132	P35213	132	145	1433B_RAT	Rattus norvegicus	246	S	Phosphorylation	PSP	NULL	14-3-3 beta	GDYFRYLsEVASGDN	14-3-3	molecular association, regulation		RAF1(DISRUPTS); PKCZ(DISRUPTS)	10620507	
IPR000308-194	0.885332368		Y	IPR000308	P63101-179	P63101	179	194	1433Z_MOUSE	Mus musculus	245	Y	Phosphorylation	PSP	NULL	14-3-3 zeta	LNFSVFYyEILNSPE	14-3-3	molecular association, regulation; phosphorylation		SHC1(INDUCES)	19218246	important for the phosphorylation of Akt and cytokine-mediated cell survival
