# Supplementary material for: The dynamic and stress-adaptive signaling hub of 14-3-3: emerging mechanisms of regulation and context-dependent protein–protein interactions
Source: Oncogene. 2018 Jun 18;37(42):5587–604. doi: 10.1038/s41388-018-0348-3 (PMC6193947; doi:10.1038/s41388-018-0348-3)
Supplement: Supplementary file 5 — Supplemental Table S2 [file 41388_2018_348_MOESM5_ESM.pdf]

Table 1. Validated stress-induced 14-3-3 interacting proteins

Condition	Interacting protein	14-3-3-binding phosphosite(s)	14-3-3 isoform(s)	Effect of 14-3-3 binding	Kinase	Phosphatase	Reference(s)
	AS160	T649 (Mouse), T642 (Human)	ζ	allows Glut4 trafficking to the plasma membrane	AKT	PP1A	Ramm et al. 2006, Howlett et al. 2007, Dubois et al. 2009, Mortenson et al. 2015, Sharma et al. 2016
	Bad	S136, S112	ζ	sequesters Bad away from Bcl-xL and Bcl-2 to allow Bax/Bak activation and MOMP	AKT, PKA, ERK, p90Rsk, Raf	Calcineurin, PP2A, PP1	Zha et al. 1996, Datta et al. 2000, Lizcano et al. 2000, Danial et al. 2003, Springer et al. 2000, Chiang et al. 2000, Ayllon et al. 2001
	Bim(EL)	S87	ζ	regulates BimEL apoptotic function	AKT	unkown	Qi et al. 2006
	Caspase-2	S135 (Xenopus); S139, S164 (Human)	ζ	prevents apoptotic signals from activating pro-apoptotic Bcl-2 family members upstream of MOMI	CAMKII	PP1	Nutt et al. 2005, Nutt et al. 2009, Kalabova et al. 2017
Insulin/ glucose	FOXO	FOXO3: T32, T253	ζ	cytoplasmic sequestration of FOXO to prevent transcriptional activation of target gene	AKT	PP2A	Singh et al. 2010, Brunet et al. 1999, Yan et al. 200
	GARNL1	T735	not specified	RalA-dependent Glut 4 plasma membrane translocatior	AKT	unknown	Chen et al. 2014
	IRS2	S573, S1137/1138	not specified	stabilizes IRS2 to propagate insulin signalin	PKA	unknown	Neukamm et al. 2013, Neukamm et al. 2012
	Myosin 1c	S701	β	requried for Glut4 transport to the cell surfac	CAMKII	unknown	Holman et al. 2008, Yip et al. 2008, Munnich et al. 201
	p85 (PI3K subunit)	S83	ζ	plasma membrane localization of PI3K to receive insulin signalin	AKT	unkown	Neal et al. 2012
	PRAS40	T246	not specified	sequestration	AKT	unknown	Kovacina et al. 2003
	TBC1D1	S237, T596	not specified	promtes Glut4 trafficking	AKT, AMPK	unknown	Chen et al. 2008, Pehmoller et al. 200
	TSC2	S939, S981, S1254, T1462	ζ	sequestration to prevent inhibition of mTORC1	AKT, MK2	unkown	Nellist et al. 2002, Cai et al. 2006, Fettweis et al. 201
	ATG9	S761	ζ	autophagy induction	ULK1, AMPK	unknown	Weerasekara et al. 2014
hypoxia	HDAC4	S246, S467, S632	ζ	sequesters HDAC4 in the cytoplasm and promotes HDAC4 dependent deacetylation of HIF1α	CAMKII, C-TAK1, EMK, hPar-1, MARK2, SIK1, Chk1	PP2A	Wang et al. 2000, McKinsey et al. 2000, Linseman et al. 2003, Chang et al. 2005, Dequiedt et al. 2006, Berdeaux et al. 2007, Kim et al. 2007, Illi et al. 2008, Yang et al. 2008, Geng et al. 2011 and Tang et al. 2016
	HIF1α	unknown	ζ	promotes portal vein tumor thrombus formation and metastasis of haptocellular carcinoma	unknown	unknown	Tang et al. 2016
	MDMX	S367	γ	allows activation of p53 and cell cylce arrest	Chk1, Chk2	unknown	Chen et al. 2005, Lee et al. 2012
	neuroglobin	S17, S50	not specified	increases neuroglobin-catalyzed nitrite to NO reductior	ERK1/2	unknown	Jayaraman et al. 2011
	REDD1	S137	not specified	allows inhibition of mTORC1	unknown	unknown	DeYoung et al. 2008
	ULK1	S555	ζ	induction of autophagy	AMPK	PP2A	Bach et al. 2011, Egan et al. 2011, Wong et al. 201
	CAF-1	unknown	β, ζ	unknown	DNA-PK	unknown	Hoek et al. 2011
	Cdc25A	S178, T507	β, ε	polyubiquitylation & degradation of Cdc25A	Chk1, Chk2	unknown	Conklin et al. 1995, Furnari et al. 1997, Sanchez et al. 1997, Chen et al. 2003
	Cdc25B	S323, S151, S230	ζ, ε, β, η	inhibits Cdc25B to prevent premature progression of the cell cycle	Chk1, MK2	unknown	Mils et al. 2000, Giles et al. 2003, Conklin et al. 1995, Furnari et al. 1997, Sanchez et al. 1997, Manke et al. 2005
	Cdc25C	S216	ζ, ε, γ	inhibits Cdc25C to prevent premature progression of the cell cycle	Chk1, MK2, C-TAK1, Chk2, pERK1/2	PP1	Peng et al. 1997, Morris et al. 2000, Dalal et al. 2004, Furnari et al. 1997, Sanchez et al. 1997, Manke et al. 2005, Peng et al. 1998, Brown et al. 1999, Eymin et al. 2006, Margolis et al. 2003
	Chk1	S317, S345, S296	ζ, β, γ		Hus1	PP2A	Sanchez et al. 1997, Chen et al. 1999, Kasahara et al. 2010, Jiang et al. 2003
	DP-2	S42, S53, S56, S58, S59, and/or S64	ε	inhibits apoptosis; facilitates cell cylce progression; promotes transcriptional activation of target gene	unknown	unknown	Milton et al. 2006
	E2F1	S31	τ	inhibits E2F1 ubiquitination; E2F1 stabilization; promotes apoptosi	ATM, ATR	unknown	Wang et al. 2004
	Exo1	S746	β, γ, ε, ζ, η, τ	inhibits binding to PAR and PCNA, promotes fork progressior	ATR, Chk1	unknown	Engels et al. 2011, Andersen et al. 201
	ING1	S199	η,β, ε, τ, γ, σ, ζ	cytoplasmic sequestration, prevents UV-induced transcriptional upregulation of target genes, reduces apoptos	unknown	unknown	Gong et al. 2006
DNA damage	Lats2	not S408, may be phospho-independent	γ, β, η, ζ	Lats2 phosphorylates 14-3-3 at S59 in response to UV irradiation to move 14-3-3 to the P-body to facilitate P-body formatior	unknown (or N/A)	unknown or N/A	Okada et al. 2011
	MDMX	S367, S342	ε, γ, τ, β, ζ, η	alters localization of MDMX, degredation of MDMX and activation of p53 in response to DNA damage	Chk1, Chk2, (AMPK in glucose deprivation)	unknown	Okamoto et al. 2005, Pereg et al. 2006, LeBron et al. 2006, Jin et al. 2006 [He et al. 2014 for AMPK in response to glucose deprivation]
	p53	S378, S366, T387	ε, γ, τ	increase p53 tetramerization and DNA binding	PKC	unknown	Waterman et al. 1998; Rajagopalan 2008; Baudier et al. 1992, Stavridi et al. 2001
	PPM1B	unknown	ε	regulates the PPM1B deactivating dephosphorylation of TAK1	unknown	unknown	Zuo et al. 2010
	RALT	S251	τ, γ, ζ, β	stabilizes RALT protein and slows down RALT trafficking to endosomes; prevents nuclear localization of RALT thereby antagonizing activation o the DNA damage response and apoptosis	Chk1	unknown	Liu et al. 2012, Takeda et al. 2013, Li et al. 2017
	RBM7	S136, S204	ε, ζ	prevents RBM7 binding to PROMPTs and their subsequent degradatio	MK2	unknown	Blasius et al. 2014
	TAK1	T184, T187, S192, S268	ε	proposed to initially activate and then inhibit TAK1 activation of NFκB translocation and transcriptional modulation	TPL2; RIPK3	PPM1B	Zuo et al. 2010, Tang et al. 2013, Santoro et al. 2017, Xiao et al. 2014; Morioka et al. 2014, Hanada et al. 2001
	TAZ	S89	not specified	sequesters TAZ in the cytosol; inhibits TAZ transcriptional coactivator functio	LATS1/2	PP1A	Kanai et al. 2000, Lei et al. 2008, Liu et al. 2011
	YAP	S127	not specified	promotes YAP localization to cytoplasm, preventing YAP's transcriptional coactivator function, and therefore suppresses the induction o apoptosis following DNA damage by cisplatin	LATS1/2	PTPN14	Vassilev et al. 2001, Basu et al. 2003, Zhao et al. 2007, Liu et al. 2013, Huang et al. 2013, Michaloglou et al. 2013
	ASK1	S967	ζ, ε, θ	inhibits ASK1 kinase activity	IKK	calcineurin, PP2A	Zhang et al. 1999, Goldman et al. 2004, Liu et al. 2006, Hsu et al. 2007, Hsu et al. 2008, Zhou et al. 2009, Cockrell et al. 2010, Seong et al. 2010, Puckett et al. 2013, Petrvalska et al. 2016
oxidative stress	FOXO3	T32, S253	ζ	sequestration in cytosol to prevent activation of cell death gene	AKT	PP2A	Brunet et al. 1999, Singh et al. 201
	RIP3	unknown	ζ, ε	allows inhibition of mTORC1 to promote cell death following 5-aminoleculinic acid-based photodynamic therap	unknown	unknown	Fettweis et al. 2017
	SelW	N/A	β, γ	Redox regulation of 14-3-3 proteins via exposed Cys on 14-3-3, disrupts interaction of 14-3-3 with ASK1	N/A	N/A	Dikiy et al. 2007, Aachmann et al. 2007, Jeon et al. 2013, Jeon et al. 2016
	SOK-1	phospho-independent?	ζ	SOK-1 phosphorylates 14-3-3ζ at S58 to trigger dissociation of 1433z-ASK1	N/A	N/A	Preisinger et al. 2004, Zhou et al. 200
