# Supplementary material for: The dynamic and stress-adaptive signaling hub of 14-3-3: emerging mechanisms of regulation and context-dependent protein–protein interactions
Source: Oncogene. 2018 Jun 18;37(42):5587–604. doi: 10.1038/s41388-018-0348-3 (PMC6193947; doi:10.1038/s41388-018-0348-3)
Supplement: Supplementary file 6 — Supplemental Table Legends [file 41388_2018_348_MOESM6_ESM.docx]

**Table S1. IPR000308 SAPH-ire and supporting data.**

Tab 1: Key for SAPH-ire FPx output table.

Tab 2: IPR000308 SAPH-ire FPx output table.

Tab 3: IPR000308 All family PTMs.

Tab 4: IPR000308 Known Functional PTMs and references.

**Table S2. Supplementary information on context-dependent 14-3-3 interactions.**
